# Control of Selectivity in Palladium-Catalyzed Oxidative Carbocyclization/Borylation of Allenynes**

**DOI:** 10.1002/anie.201301167

**Published:** 2013-05-17

**Authors:** Youqian Deng, Teresa Bartholomeyzik, Jan-E Bäckvall

**Affiliations:** Department of Organic Chemistry, Arrhenius Laboratory, Stockholm UniversitySE-106 91 Stockholm (Sweden) E-mail: jeb@organ.su.se

**Keywords:** allenes, boronates, cyclization, oxidation, palladium

Organoboronates are convenient and versatile reagents owing to their comparatively low toxicity, high functional group compatibility, and good stability.^1^ Moreover, these compounds can easily be oxidized to alcohols^2^ or used to construct new C–C bonds by Suzuki–Miyaura cross-couplings.^3^ Because of the broad applications of these boronates, many borylation methods have been developed.^4^–^7^ Amongst the routes reported for C–B bond formation the most common are Miyaura borylation,^4^ hydroboration,^5^ and the reaction of lithium or magnesium organometallic compounds with borate esters.^1^ In addition, recent developments in transition-metal-catalyzed C–H borylation reactions have also provided efficient access to boronates.^6^

Furthermore, by combining the borylation with a C–C bond-forming cyclization, complex molecules suitable for various further functionalizations could be obtained in one step.^8^–^11^, 13b,d Such borylating carbocyclizations have been successfully developed by the group of Cárdenas.^9^–^11^ Starting from unsaturated compounds, such as enynes,^9^ enediynes,^10^ enallenes,^11^ and allenynes,^11^ homoallylic or allylic boronates were prepared under palladium(0) catalysis. For instance, the non-oxidative borylating carbocyclization of allenynes **1** in the presence of bis(pinacolato)diboron (B_2_pin_2_) yielded two isomers (**2** and **3**), where borylation occurred at the allene (Scheme 1 a).^11^

**Scheme 1:**
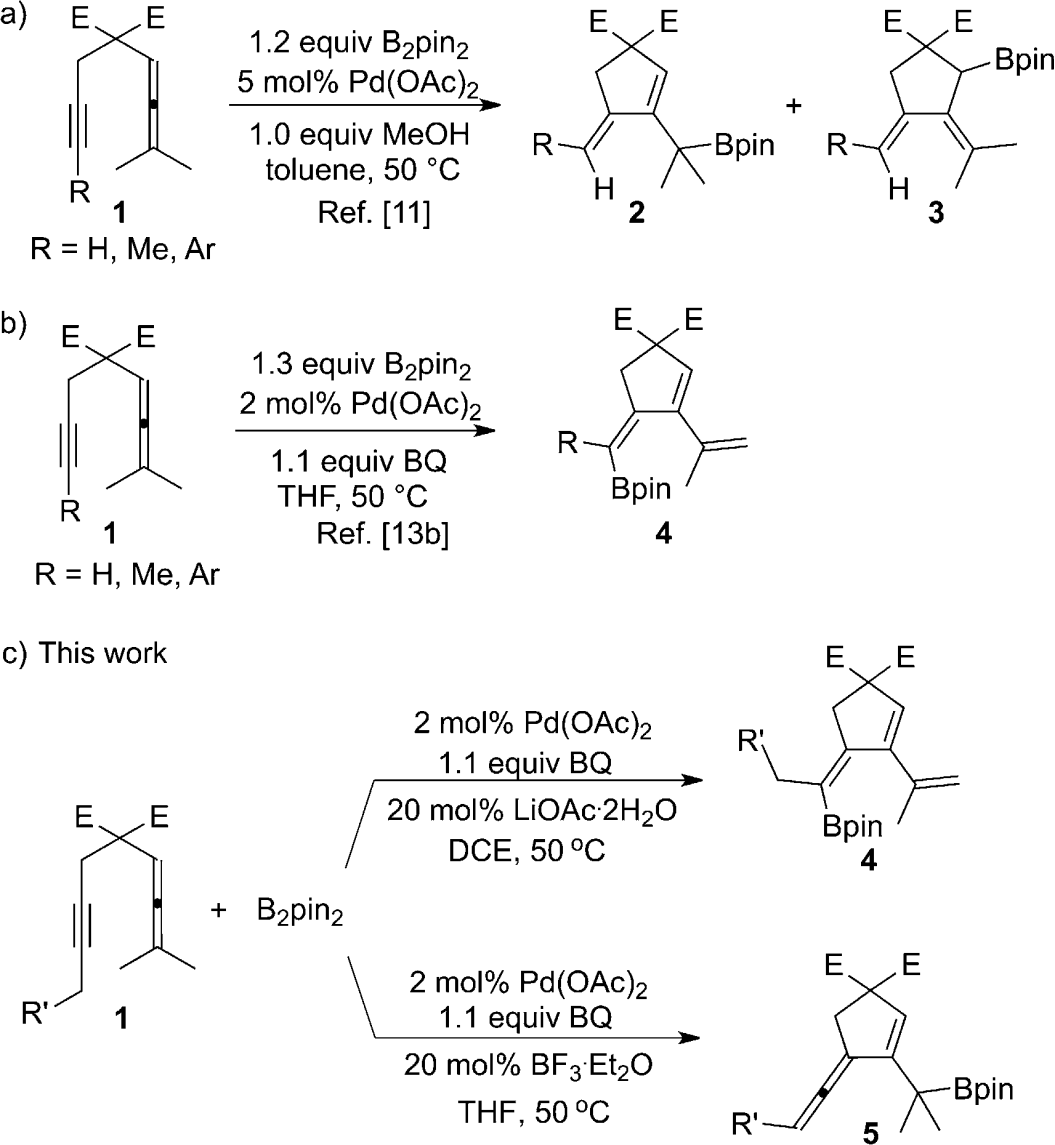
Palladium-catalyzed borylating carbocyclizations of allenynes: a) under non-oxidative conditions;^11^ b) under oxidative conditions;13b c) under selective oxidative conditions. E=CO_2_Me.

In ongoing investigations our research group has been studying oxidative Pd^II^-catalyzed carbocyclizations of various unsaturated molecules.^12^–^15^ Recently we accomplished the carbocyclization/arylation of allenynes with arylboronic acids.13b Also, some preliminary results regarding carbocyclization/borylation were obtained with differently substituted 1,5-allenynes (**1**; R=H, Me, Ar), which only gave borylated triene products **4** (Scheme 1 b).13b However, under carbocyclization/arylation conditions alkyl-substituted allenynes afforded two different constitutional isomers (arylated trienes and arylated vinylallenes) in a ratio determined by the substitution on the starting allenyne.13b The aim of the present study was to develop a carbocyclization/borylation that can be directed towards either a borylated triene or a borylated vinyllallene by control of the reaction conditions (Scheme 1 c). We now report a highly selective oxidative carbocyclization/borylation of allenynes **1** with B_2_pin_2_ under Pd^II^ catalysis with *p*-benzoquinone (BQ) as the oxidant. The use of LiOAc⋅2 H_2_O in 1,2-dichloroethane (DCE) or BF_3_⋅Et_2_O in THF addressed the issue of selectivity, to give either borylated trienes **4** or borylated vinylallenes **5**, respectively.

We first studied the reaction of ethyl-substituted allenyne **1 a** with B_2_pin_2_ under the original carbocyclization/borylation conditions (Scheme 1 b).13b The use of a catalytic amount of palladium acetate (2 mol %) and stoichiometric amounts of BQ (1.1 equiv) in THF at 50 °C led to an isomeric mixture of borylated triene **4 a** and borylated vinylallene **5 a** in 28 % and 14 % yield, respectively (Table 1, entry 1). Analyzing the effect of different solvents showed that a higher selectivity for **4 a** was obtained when DCE was used as the solvent (in Table 1, entry 4 vs. entries 1–3). Furthermore, upon the addition of catalytic amounts (20 mol %) of a basic salt, such as Na_2_CO_3_, NaOAc, or LiOAc⋅2 H_2_O, formation of triene **4 a** was favored (Table 1, entries 5–7). Boronate **4 a** was obtained in high selectivity in 73 % yield with LiOAc⋅2 H_2_O as the base additive and with DCE as the solvent (Table 1, entry 7; defined as Method A). An increase of the amount of LiOAc⋅2 H_2_O to 50 mol % gave no additional improvement in selectivity or yield (Table 1, entry 8).

**Table 1:** Solvent and additive effect in the selective formation of triene 4 a or vinylallene 5 a.[a]

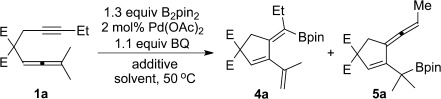					

Entry	Solvent	Additive (20 mol %)	Time [h]	Yield of4 a/5 a [%][b]	4 a/5 a
1	THF	–	15	28:14	2:1
2	cyclohexane	–	15	33:34	1:1
3	CH_2_Cl_2_	–	15	39:35	1:1
4	DCE	–	15	61:13	5:1
5	DCE	Na_2_CO_3_	15	70:7	10:1
6	DCE	NaOAc	15	67:6	11:1
7	DCE	LiOAc⋅2H_2_O	15	73:7	10:1
8[c]	DCE	LiOAc⋅2H_2_O	15	71:10	7:1
9	THF	HOAc	20	19:16	ca. 1:1
10	THF	*p*-TSA	20	0	–
11	THF	BF_3_⋅Et_2_O	20	3:78	1:26
12[d]	THF	–	20	5:60	1:12
13	THF	Et_3_B	20	8:24	1:3

[a] Unless otherwise noted, **1 a**, B_2_pin_2_ (1.3 equiv), Pd(OAc)_2_ (2 mol %), BQ (1.1 equiv), and indicated additive (20 mol %) were dissolved in the indicated solvent (5 mL mmol^−1^) and stirred at 50 °C in a sealed tube.

[b] Yield was determined by ^1^H NMR spectroscopy using anisole as internal standard.

[c] 50 mol % of LiOAc⋅2H_2_O was added.

[d] 2 mol % of [Pd(CH_3_CN)_4_][(BF_4_)_2_] was used in place of Pd(OAc)_2_. E=CO_2_Me.

The finding that the addition of a basic salt substantially enhanced the selective formation of alkenyl boronate **4** encouraged us to study the effect of acidic reaction conditions. To our surprise, the addition of a Brønsted acid, such as HOAc, generated an approximately 1:1 mixture of **4 a** and **5 a** in moderate yields (Table 1, entry 9) and the use of *p*-toluenesulfonic acid (*p*-TSA) even did not afford any borylation products (Table 1, entry 10). However, the use of a Lewis acid, BF_3_⋅Et_2_O, resulted in a high selectivity for **5** and afforded products **4 a** and **5 a** in 3 % and 78 % yield, respectively (Table 1, entry 11; defined as Method B). Notably when the cationic palladium catalyst [Pd(CH_3_CN)_4_][(BF_4_)_2_] was used the same trend in selectivity was seen but a lower yield was obtained (Table 1, entry 12 vs. entry 11).^16^ The structurally similar Lewis acid BEt_3_ was also tried and moderate selectivity for **5 a** over **4 a** was seen with low yields of products (Table 1, entry 13).

With the optimized conditions for the selective formation of borylated triene **4 a** established, we applied them to differently substituted allenynes (Table 2). The allenynes bearing a methyl group on the alkyne moiety (**1 b** and **1 c**) afforded the borylated trienes as the sole products (Table 2, entries 2 and 3). For substrates with a longer alkyl group (**1 d** and **1 f**) on the alkyne moiety, the competing allene formation took place to a notable extent (Table 2, entries 4 and 6), but the corresponding triene products **4 d** and **4 f**/**4 f**′ could be isolated in good to moderate yields. In those cases where the substrates are unsymmetrically substituted at the allene moiety (**1 c** and **1 e**) a comparatively high selectivity for the triene products was observed (Table 2, entries 3 and 5). However, a mixture of borylated triene products was obtained, with a preference for formation of the products with the more substituted double bond. Products **4 c** and **4 e** were obtained as a mixture of *Z*/*E* isomers in a ratio of 3.3:1 and 3.5:1, respectively. The allenyne **1 f** with a cyclohexylidene group on the allene cyclized to give a mixture of isomers **4 f** and **4 f**′, where the formation of **4 f**′ could be explained by a Pd-catalyzed isomerization of **4 f** (Table 2, entry 6; for the detailed mechanism, please see the Supporting Information). Moreover, the reaction of allenyne **1 g**, having two benzyl ether groups on the linker part X, also gave borylated triene product **4 g** selectively in 57 % yield (Table 2, entry 7), thus proving that the malonate group of linker X is not necessary for a successful transformation.

**Table 2:** Selective carbocyclization of allenynes 1 yielding borylated trienes 4[a]

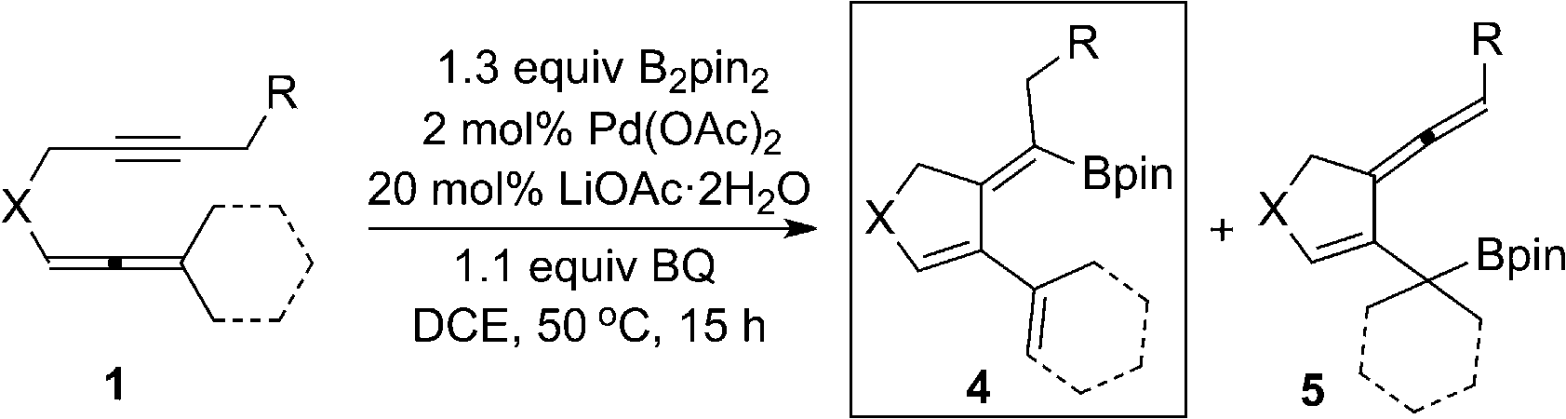				

Entry	Allenyne	Product	4/5[b]	Yield of4[%],[c] ratio[b]
1	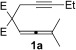	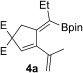	10:1	73
2	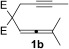	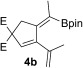	99:1	92
3	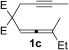	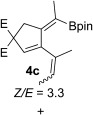	99:1	55 **4 c**/**4 c**′=2.4:1
		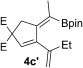		
4	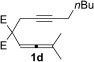	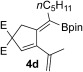	9:1 >11:1	81 92[d]
5	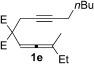	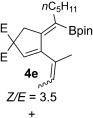	18:1	65 **4 e**/**4 e**′=2.3:1
		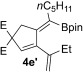		
6	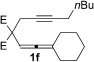	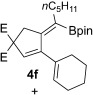	5:1	48 **4 f**/**4 f**′=1.4:1
		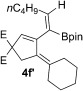			
7	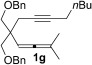	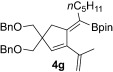	>20:1	57

[a] Unless otherwise noted, **1** (0.1–0.2 mmol), B_2_pin_2_ (1.3 equiv), Pd(OAc)_2_ (2 mol %), BQ (1.1 equiv), and LiOAc⋅2 H_2_O (20 mol %) were dissolved in DCE (5 mL mmol^−1^) and stirred at 50 °C for 15 h.

[b] The ratio was determined by ^1^H NMR analysis of the reaction mixture.

[c] Yield of the isolated product.

[d] 1 mmol of **1 d** was used. E=CO_2_Me.

The optimized reaction conditions for the selective formation of borylated vinylallene **5** (20 mol % of BF_3_⋅Et_2_O, Method B) were applied to various allenynes (Table 3). Allenynes **1 a**–**1 f** were transformed into vinylallenic boronates **5 a**–**5 f**; for most cases the yield was between 70 % and 80 % and the formation of the corresponding triene isomers **4 a**–**4 f** was efficiently suppressed. Even the methyl-substituted substrate **1 b**, which intrinsically favors formation of triene **4 b**,13b displayed opposite selectivity under these reaction conditions, that is, favoring vinylallene formation (Table 3, entry 2). The reaction of allenyne **1 g** under the standard conditions of Table 3 was sluggish and did not give the desired product **5 g**, probably because of the incompatibility between the benzyl ether group and BF_3_⋅Et_2_O. However, by switching the palladium catalyst to [Pd(CH_3_CN)_4_][(BF_4_)_2_] and in the absence of BF_3_⋅Et_2_O, product **5 g** was obtained in 37 % yield (entry 7).

**Table 3:** Selective carbocyclization of allenynes 1 yielding borylated vinylallenes 5[a]

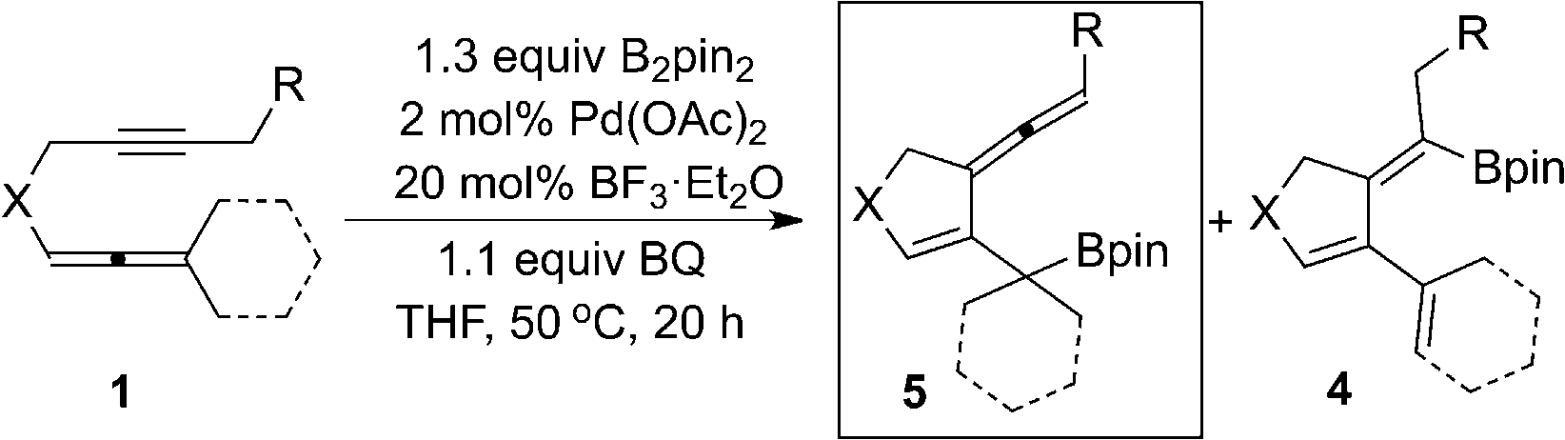				

Entry	Allenyne	Product	5/4[b]	Yield of 5 [%][c]
1	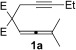	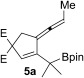	>20:1	77
2	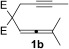	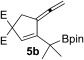	>20:1	73
3	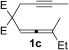	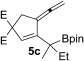	>20:1	56
4	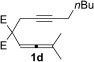	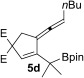	>20:1	79 87[d]
5	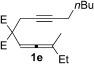	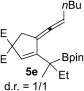	20:1	77
6	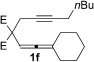	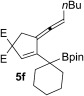	>20:1	70
7[e]	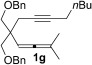	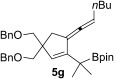	>20:1	37

[a] Unless otherwise noted, **1** (0.1–0.2 mmol), B_2_pin_2_ (1.3 equiv), Pd(OAc)_2_ (2 mol %), BQ (1.1 equiv), and BF_3_⋅Et_2_O (20 mol %) were dissolved in THF (5 mL mmol^−1^) and stirred at 50 °C for 20 h.

[b] Ratio determined by ^1^H NMR analysis of the crude reaction mixture.

[c] Yield of the isolated product.

[d] 1 mmol of **1 d** was used.

[e] 2 mol % of [Pd(CH_3_CN)_4_][(BF_4_)_2_] was used in place of Pd(OAc)_2_ and BF_3_⋅Et_2_O. E=CO_2_Me.

To gain further insights into the mechanism of the oxidative carbocyclization/borylation, kinetic deuterium isotope effects were studied (Scheme 2). An intermolecular competition experiment using **1 d** and its hexadeuterated derivative [D_6_]-**1 d** under the conditions for selective triene formation for 1 h provided a large intermolecular KIE value of 6.7^17^ (Scheme 2 a). This result indicates that the allylic C–H bond cleavage involved has to occur prior to any irreversible step of the reaction, for example, the carbocyclization step.^18^ On the other hand, when a 1:1 mixture of **1 d** and [D_2_]-**1 d** was subjected to the conditions for selective vinylallene formation for 1 h the ratio between **5 d** and [D_1_]-**5 d** was 2.4, from which the KIE was determined to 2.7^19^ (Scheme 2 b).^17^ The intrinsic KIE from intramolecular competition for vinylallene formation was determined to 5.3^17^ by the use of [D_1_]-**1 d** as the allenyne substrate (Scheme 2 c). The results in Scheme 2 b and 2 c indicate that the propargylic C–H bond cleavage does not fully determine the selectivity between **5 d** and [D_1_]-**5 d** in the competitive experiment (Scheme 2 b).^18^

**Scheme 2:**
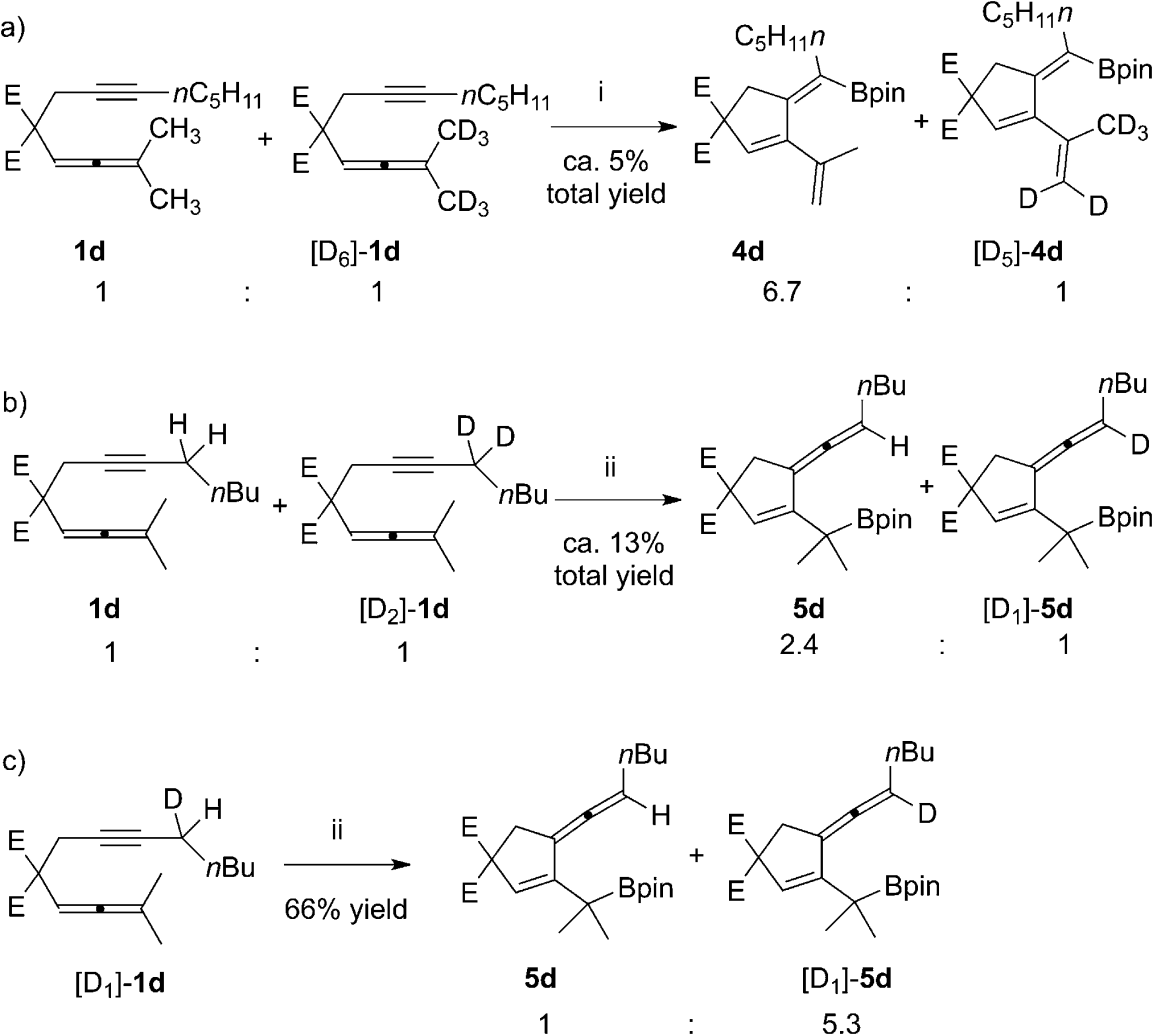
Kinetic isotope effect study. Reaction conditions: i) B_2_pin_2_ (1.3 equiv), Pd(OAc)_2_ (2 mol %), BQ (1.1 equiv), LiOAc⋅2H_2_O (20 mol %), DCE, 50 °C, 1 h. ii) B_2_pin_2_ (1.3 equiv), Pd(OAc)_2_ (2 mol %), BQ (1.1 equiv), BF_3_⋅Et_2_O (20 mol %), THF, 50 °C, 1 h for b) and 20 h for c). E=CO_2_Me.

Three control experiments with allenyne **1 d** and the corresponding deuterium-labeled allenynes [D_2_]-**1 d** and [D_6_]-**1 d** were conducted under palladium catalysis in the absence of any additional basic or acidic additive and using DCE as the solvent (Scheme 3). Under these conditions the reaction of **1 d** gave a mixture of **4 d** and **5 d** in a ratio of 3.8:1 (Scheme 3 a). When substrate [D_2_]-**1 d** was employed (Scheme 3 b) under the same reaction conditions, the ratio increased to 12.3:1.^20^ Allenyne [D_6_]-**1 d** showed the opposite selectivity, with [D_5_]-**4 d** and [D_6_]-**5 d** being formed in a ratio of 1:5.1^20^ (Scheme 3 c).

**Scheme 3:**
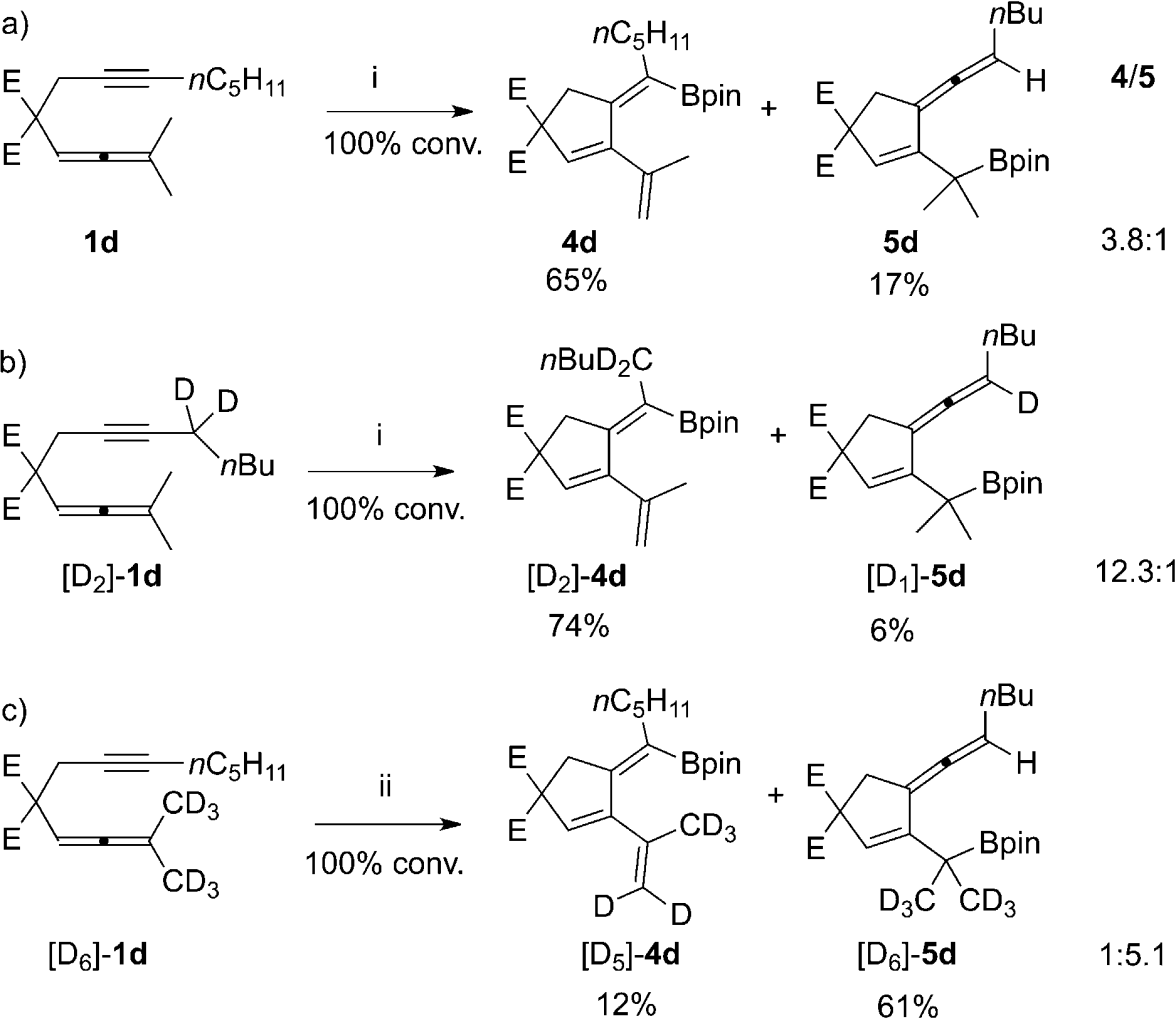
Effect of isotope substitution on product distribution. Reaction conditions: i) B_2_pin_2_ (1.3 equiv), Pd(OAc)_2_ (2 mol %), BQ (1.1 equiv), DCE, 50 °C, 15 h. ii) B_2_pin_2_ (1.3 equiv), Pd(OAc)_2_ (5 mol %), BQ (1.1 equiv), DCE, 50 °C, 15 h. E=CO_2_Me.

The results in Scheme 2 and Scheme 3 indicate that competing allylic and propargylic C–H bond cleavage occurs in **1**, and this determines the ratio of boronates **4** and **5** (Scheme 4). The allene attack on Pd^II^ complex **A** through allylic C–H bond cleavage^12^, 13a–d would give **B** and subsequent alkyne insertion would generate intermediate **C**. Transmetalation of **C** with B_2_pin_2_ and reductive elimination would form product **4**. The competing alkyne attack through propargylic C–H bond cleavage in **A** would produce allenylpalladium intermediate **D**. Intramolecular vinylpalladation of the allene moiety would generate (π-allyl)palladium intermediate **E**. Transmetalation with B_2_pin_2_ and subsequent reductive elimination would give **5**. The mechanism in Scheme 4 is supported by the kinetic isotope effects and the experiments with deuterium-labeled compounds (Scheme 2 and Scheme 3). The lower kinetic isotope effect observed for the competitive experiment in Scheme 2 b compared to the intramolecular experiment in Scheme 2 c may reflect that **1** and **A** are not in full equilibrium under the conditions for formation of **5**. In the path for formation of **5** it is likely that BF_3_⋅Et_2_O creates a cationic palladium species, which interacts better with the acetylene compared to the allene in **A**.^21^

**Scheme 4:**
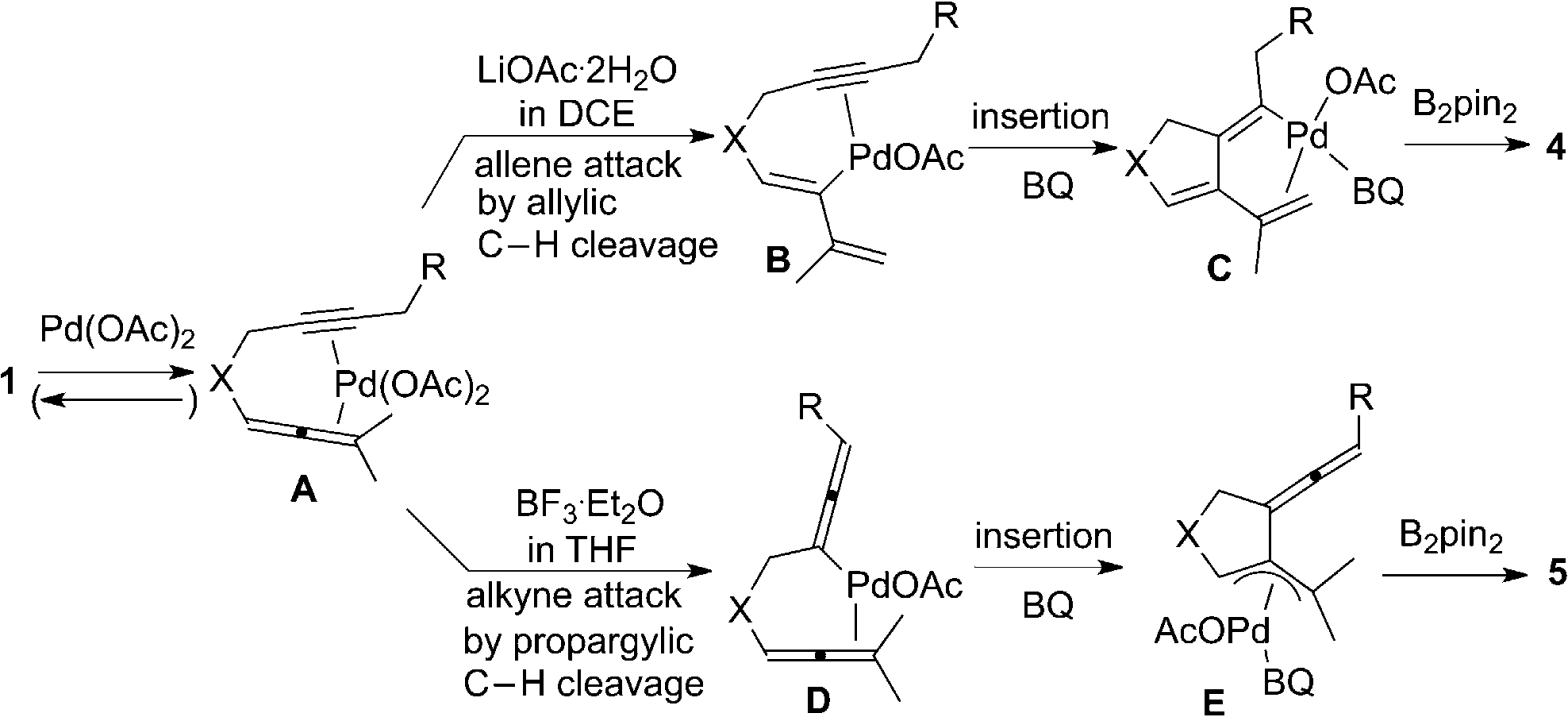
Proposed mechanism for palladium-catalyzed oxidative selective carbocyclization/borylation of allenyne **1**.

In summary, we have developed an unprecedented selective Pd^II^-catalyzed carbocyclization/borylation of allenynes under oxidative conditions. By controlling the reaction conditions the reaction can be directed to either the triene **4** or the vinylallene **5**. On the basis of the results of deuterium-labeling experiments, we propose that the reactions of allenynes proceed through competing allylic and propargylic C–H bond cleavage pathways to give borylated trienes and borylated vinylallenes, respectively.

## Experimental Section

Typical experimental procedure for palladium-catalyzed oxidative borylating carbocyclization of allenyne **1** to boronate **4**: **1 a** (26.0 mg, 0.10 mmol) and 0.5 mL of DCE were added to a mixture of B_2_pin_2_ (33.1 mg, 0.13 mmol), BQ (12.2 mg, 0.11 mmol), Pd(OAc)_2_ (0.5 mg, 0.002 mmol), and LiOAc⋅2H_2_O (1.8 mg, 0.02 mmol) at RT. The reaction was stirred at 50 °C for 15 h. After the reaction was complete, as monitored by TLC, evaporation and column chromatography on silica gel (pentane/ethyl acetate=10:1) afforded **4 a** (27.9 mg, 73 %) as a liquid; ^1^H NMR (500 MHz, CDCl_3_): *δ*=5.93 (s, 1 H), 5.02–5.00 (m, 2 H), 3.72 (s, 6 H), 3.20 (s, 2 H), 2.19 (q, *J*=7.5 Hz, 2 H), 1.95 (s, 3 H), 1.26 (s, 12 H), 1.02 ppm (t, *J*=7.5 Hz, 3 H); ^13^C NMR (125 MHz, CDCl_3_): *δ*=171.1, 151.1, 147.9, 139.8, 129.1, 116.6, 83.3, 63.0, 52.9, 37.5, 27.0, 25.2, 23.8, 13.4 ppm; HRMS (ESI): calc. for C_21_H_31_BNaO_6_ [*M*+Na]^+^: 413.2110; found: 413.2113.

Typical experimental procedure for palladium-catalyzed oxidative borylating carbocyclization of allenyne **1** to boronate **5**: **1 a** (52.7 mg, 0.20 mmol) and 1.0 mL of THF were added to a mixture of B_2_pin_2_ (66.2 mg, 0.26 mmol), BQ (24.0 mg, 0.22 mmol), Pd(OAc)_2_ (1.0 mg, 0.004 mmol), and BF_3_⋅Et_2_O (6 μL, 0.04 mmol) at RT. The reaction was stirred at 50 °C for 20 h. After the reaction was complete, as monitored by TLC, evaporation and column chromatography on silica gel (pentane/ethyl acetate=10/1) afforded **5 a** (59.6 mg, 77 %) as a liquid; ^1^H NMR (400 MHz, CDCl_3_): *δ*=5.55 (d, *J*=1.6 Hz, 1 H), 5.34–5.23 (m, 1 H), 3.716 (s, 3 H), 3.715 (s, 3 H), 3.19–3.17 (m, 2 H), 1.68 (d, *J*=7.2 Hz, 3 H), 1.18 (s, 12 H), 1.17 (s, 3 H), 1.13 ppm (s, 3 H); ^13^C NMR (100 MHz, CDCl_3_): *δ*=199.1, 171.5, 171.3, 153.8, 122.9, 107.1, 91.2, 83.1, 63.5, 52.7, 36.5, 25.0, 24.7, 24.5, 23.9, 23.8, 14.8 ppm; HRMS (ESI): calcd for C_21_H_31_BNaO_6_ [*M*+Na]^+^: 413.2110; found: 413.2103.

## Supplementary material

As a service to our authors and readers, this journal provides supporting information supplied by the authors. Such materials are peer reviewed and may be re-organized for online delivery, but are not copy-edited or typeset. Technical support issues arising from supporting information (other than missing files) should be addressed to the authors.
