# Supplementary material for: Control of Selectivity in Palladium-Catalyzed Oxidative Carbocyclization/Borylation of Allenynes
Source: Angew Chem Int Ed Engl. 2013 May 17;52(24):6283–7. doi: 10.1002/anie.201301167 (PMC3734627; doi:10.1002/anie.201301167)
Supplement: Supplementary file 1 [file anie0052-6283-SD1.pdf]

Angewandte Chemie
Eine Zeitschrift der Gesellschaft Deutscher Chemiker

Supporting Information

© Wiley-VCH 2013

69451 Weinheim, Germany

Control of Selectivity in Palladium-Catalyzed Oxidative Carbocyclization/Borylation of Allenynes**

*Youqian Deng, Teresa Bartholomeyzik, and Jan-E. Bäckvall**

anie_201301167_sm_miscellaneous_information.pdf

Contents

General remarks	S2
Preparation of allenynes	S3
General procedure for selective formation of borylated trienes **4**	S3
General procedure for selective formation of borylated vinylallenes **5**	S8
Explanation for the formation of **4f'** from **4f**	S12
Application of product **5d**	S13
Kinetic isotope effect experiments	S14
Alternative mechanism	S21
References	S21
Copies of spectra	S22

Experimental Section

General remarks

Unless otherwise noted, all reagents were used as received from the commercial suppliers. B_2pin_2 was commercially available from Combi-Blocks. $Pd(OAc)_2$ was obtained from Pressure Chemicals and used without further purification. Palladium-catalyzed cyclizations were performed without any efforts to exclude moisture. Dry solvents (Et_2O, THF) were obtained from a VAC Solvent Purifier. Reactions were monitored using thin-layer chromatography (SiO_2). TLC plates were visualized with UV light (254 nm) or $KMnO_4$ stain. Flash chromatography was carried out with 60Å (particle size 35-70 μm) normal flash silica gel. NMR spectra were recorded at 400 MHz (^{1}H) or 500 MHz (^{1}H) and at 100 MHz (^{13}C) or 125 MHz (^{13}C), respectively. Chemical shifts (δ) are reported in ppm, using the residual solvent peak in $CDCl_3$ (δ_H = 7.26 and δ_C = 77.0 ppm) as internal standard, and coupling constants (*J*) are given in Hz. HRMS were recorded using ESI-TOF techniques.

General procedure for preparation of starting materials

All bromoallenes were prepared according to the procedure published by Landor[1a] with minor modifications.[1b]

Dimethyl propargylmalonate is commercially available from Aldrich. Other alkyl substituted dimethyl propargylmalonates were prepared as described in the literature.[2]

Allenynes **1a-g** were prepared as previously described.[3]

General procedure for oxidative carbocyclization/borylation for the formation of trienes 4

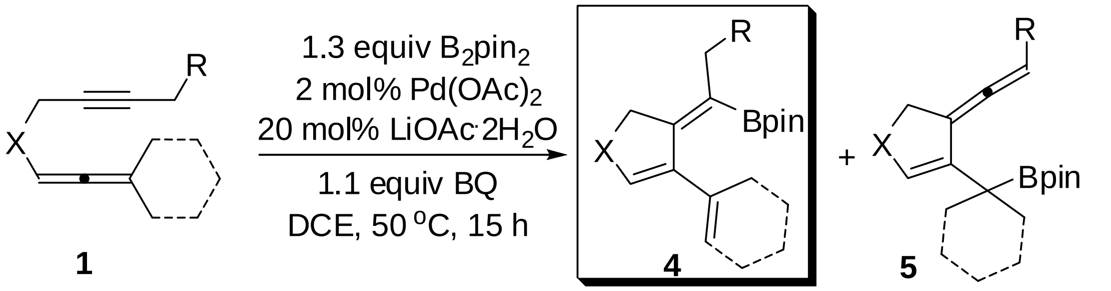

*Representative procedure A for the synthesis of **4**. (E)-dimethyl 3-(prop-1-en-2-yl)-4-(1-(4,4,5,5-tetramethyl-1,3,2-dioxaborolan-2-yl)-propylidene)cyclopent-2-ene-1,1-dicarboxylate (**4a**)*

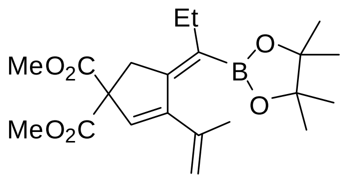

To a mixture of B_2pin_2 (33.1 mg, 0.13 mmol), BQ (12.2 mg, 0.11 mmol), $Pd(OAc)_2$ (0.5 mg, 0.002 mmol), and $LiOAc·2H_2O$ (1.8 mg, 0.02 mmol) were added **1a** (26.0 mg, 0.10 mmol) and 0.5 mL of DCE at rt. The reaction was stirred at 50 °C for 15 h. After the reaction was complete as monitored by TLC, evaporation and column chromatography on silica gel (pentane/ethyl acetate = 10/1) afforded **4a** (27.9 mg,

73%) as a liquid; ^{1}H NMR (500 MHz, $CDCl_3$): δ 5.93 (s, 1H), 5.02-5.00 (m, 2H), 3.72 (s, 6H), 3.20 (s, 2H), 2.19 (q, J = 7.5 Hz, 2H), 1.95 (s, 3H), 1.26 (s, 12H), 1.02 (t, J = 7.5 Hz, 3H); ^{13}C NMR (125 MHz, $CDCl_3$): δ 171.1, 151.1, 147.9, 139.8, 129.1, 116.6, 83.3, 63.0, 52.9, 37.5, 27.0, 25.2, 23.8, 13.4; HRMS (ESI): calc. for $C_{21}H_{31}BNaO_6$ $[M+Na]^+$: 413.2110; found: 413.2113.

*(E)-dimethyl 3-(prop-1-en-2-yl)-4-(1-(4,4,5,5-tetramethyl-1,3,2-dioxaborolan-2-yl)-ethylidene)cyclopent-2-ene-1,1-dicarboxylate (**4b**).*[3b]

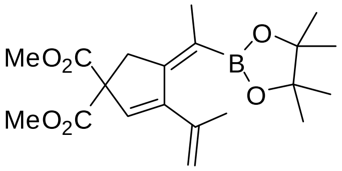

92% isolated yield, solid. ^{1}H NMR (400 MHz, $CDCl_3$): δ 5.91 (s, 1H), 5.01-4.96 (m, 2H), 3.72 (s, 6H), 3.16 (s, 2H), 1.95 (s, 3H), 1.78 (s, 3H), 1.24 (s, 12H).

*Compound **4c** and **4c'** were obtained as an inseparable mixture.*

*(E)-dimethyl 3-((Z)-but-2-en-2-yl)-4-(1-(4,4,5,5-tetramethyl-1,3,2-dioxaborolan-2-yl)-ethylidene)cyclopent-2-ene-1,1-dicarboxylate (Z-**4c**)*

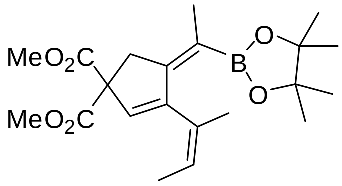

Liquid; ^{1}H NMR (500 MHz, $CDCl_3$): δ 5.81 (d, J = 0.5 Hz, 1H), 5.37 (qt, J_1 = 6.5 Hz, J_2 = 1.5 Hz, 1H), 3.71 (s, 6H), 3.14 (s, 2H), 1.84 (d, J = 1.5 Hz, 3H), 1.76 (s, 3H), 1.45 (dd, J_1 = 6.5 Hz, J_2 = 1.5 Hz, 3H), 1.24 (s, 12H); HRMS (ESI): calc. for $C_{21}H_{31}BNaO_6$ $[M+Na]^+$: 413.2110; found: 413.2116.

*(E)-dimethyl 3-(but-1-en-2-yl)-4-(1-(4,4,5,5-tetramethyl-1,3,2-dioxaborolan-2-yl)-ethylidene)cyclopent-2-ene-1,1-dicarboxylate (**4c'**)*

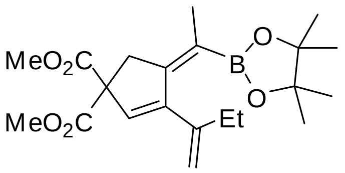

Liquid; ^{1}H NMR (500 MHz, $CDCl_3$): δ 5.89 (s, 1H), 4.990 (s, 1H), 4.989 (s, 1H), 3.71 (s, 6H), 3.16 (s, 2H), 2.26 (qt, J_1 = 7.5 Hz, J_2 = 1.5 Hz, 2H), 1.76 (s, 3H), 1.02 (t, J = 7.5 Hz, 3H); HRMS (ESI): calc. for $C_{21}H_{31}BNaO_6$ $[M+Na]^+$: 413.2110; found: 413.2116.

*(E)-dimethyl 3-(prop-1-en-2-yl)-4-(1-(4,4,5,5-tetramethyl-1,3,2-dioxaborolan-2-yl)-hexylidene)cyclopent-2-ene-1,1-dicarboxylate (**4d**)*

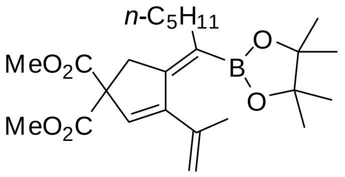

81% isolated yield, liquid. ^{1}H NMR (400 MHz, $CDCl_3$): δ 5.92 (s, 1H), 5.01-4.99 (m, 2H), 3.72 (s, 6H), 3.19 (s, 2H), 2.18-2.11 (m, 2H), 1.95 (s, 3H), 1.42-1.35 (m, 2H), 1.34-1.26 (m, 4H), 1.25 (s, 12H), 0.88 (t, J = 6.8 Hz, 3H); ^{13}C NMR (100 MHz, $CDCl_3$): δ 171.1, 151.1, 148.0, 139.8, 129.0, 116.5, 83.3, 63.0, 52.8, 37.7, 34.1, 32.0, 28.6, 25.2, 25.0, 23.8, 22.5, 14.0; HRMS (ESI): calc. for $C_{24}H_{37}BNaO_6$ $[M+Na]^+$: 455.2580; found: 455.2577.

*[D_2]-(E)-dimethyl 3-(prop-1-en-2-yl)-4-(1-(4,4,5,5-tetramethyl-1,3,2-dioxaborolan-2-yl) hexylidene)cyclopent-2-ene-1,1-dicarboxylate ([D_2]-**4d**)*

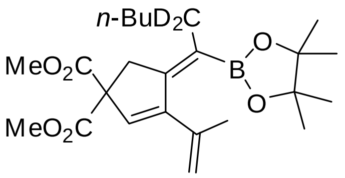

84% isolated yield (Scheme 3b), liquid. ^{1}H NMR (400 MHz, $CDCl_3$): δ 5.91 (s, 1H), 5.00-4.98 (m, 2H), 3.70 (s, 6H), 3.18 (s, 2H), 1.93 (t, J = 1.2 Hz, 3H), 1.40-1.34 (m, 2H), 1.33-1.25 (m, 4H), 1.23 (s, 12H), 0.87 (t, J = 7.2 Hz, 3H); ^{13}C NMR (100 MHz, $CDCl_3$): δ 171.0, 151.1, 148.0, 139.8, 129.0, 116.5, 83.2, 62.9, 52.8, 37.7, 31.9, 28.4,

25.2, 24.9, 23.7, 22.5, 13.9; HRMS (ESI): calc. for $C_{24}H_{35}BD_2NaO_6$ $[M+Na]^+$: 457.2705; found: 457.2706.

*Compound **4e** and **4e'** were obtained as an inseparable mixture.*

*(E)-dimethyl 3-((Z)-but-2-en-2-yl)-4-(1-(4,4,5,5-tetramethyl-1,3,2-dioxaborolan-2-yl)-hexylidene)cyclopent-2-ene-1,1-dicarboxylate (Z-**4e**)*

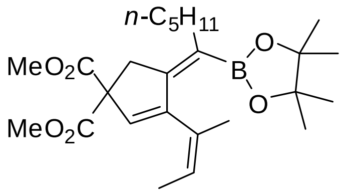

Liquid; ^{1}H NMR (500 MHz, $CDCl_3$): δ 5.82 (s, 1H), 5.37 (qq, J_1 = 6.5 Hz, J_2 = 1.5 Hz, 1H), 3.72 (s, 6H), 3.18 (s, 2H), 2.16-2.10 (m, 2H), 1.86 (t, J= 6.5 Hz, 3H), 1.45 (dq, J_1 = 6.5 Hz, J_2 = 1.5 Hz, 3H), 1.42-1.36 (m, 2H), 1.34-1.26 (m, 4H), 1.25 (s, 12H), 0.88 (t, J = 6.8 Hz, 3H); HRMS (ESI): calc. for $C_{25}H_{39}BNaO_6$ $[M+Na]^+$: 469.2736; found: 469.2735.

*(E)-dimethyl 3-cyclohexenyl-4-(1-(4,4,5,5-tetramethyl-1,3,2-dioxaborolan-2-yl)-hexylidene) cyclopent-2-ene-1,1-dicarboxylate (**4f**)*

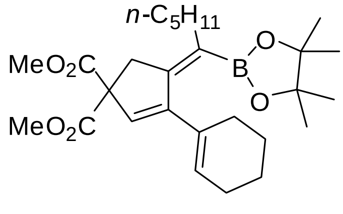

28% isolated yield, liquid. ^{1}H NMR (500 MHz, $CDCl_3$): δ 5.91 (s, 1H), 5.77-5.72 (m, 1H), 3.72 (s, 6H), 3.19 (s, 2H), 2.22-2.14 (m, 4H), 2.11-2.04 (m, 2H), 1.70-1.64 (m, 2H), 1.63-1.55 (m, 2H), 1.44-1.36 (m, 2H), 1.34-1.26 (m, 4H), 1.25 (s, 12H), 0.89 (t, J = 7.0 Hz, 3H); ^{13}C NMR (125 MHz, $CDCl_3$): δ 171.2, 151.7, 147.6, 133.6, 128.0, 126.3, 82.9, 62.5, 52.8, 38.6, 34.3, 32.1, 28.8, 28.2, 25.3, 25.2, 22.5, 22.4, 21.8, 14.0; HRMS (ESI): calc. for $C_{27}H_{41}BNaO_6$ $[M+Na]^+$: 495.2893; found: 495.2899.

*(Z)-Dimethyl 4-cyclohexylidene-3-(1-(4,4,5,5-tetramethyl-1,3,2-dioxaborolan-2-yl)-hex-1-enyl)cyclopent-2-ene-1,1-dicarboxylate (**4f'**)* (less polar)

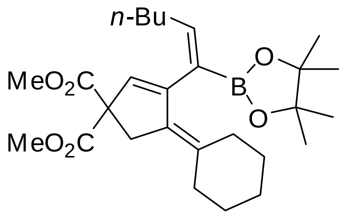

20% isolated yield, liquid. ^{1}H NMR (500 MHz, $CDCl_3$): δ 5.75 (s, 1H), 5.28-5.22 (m, 1H), 3.73 (s, 6H), 3.10 (s, 2H), 2.21-2.16 (m, 2H), 2.15-2.08 (m, 2H), 2.06-1.97 (m, 2H), 1.68-1.61 (m, 2H), 1.60-1.56 (m, 2H), 1.38-1.32 (m, 2H), 1.31-1.22 (m, 4H), 1.13 (s, 12H), 0.88 (t, J = 7.0 Hz, 3H); ^{13}C NMR (125 MHz, $CDCl_3$): δ 171.4, 152.5, 143.7, 142.9, 125.9, 121.3, 82.8, 63.8, 52.7, 35.1, 31.8, 31.7, 29.5, 29.0, 27.4, 24.6, 22.6, 22.5, 22.1, 14.0; HRMS (ESI): calc. for $C_{27}H_{41}BNaO_6$ $[M+Na]^+$: 495.2893; found: 495.2902.

*(E)-2-(1-(4,4-Bis(benzyloxymethyl)-2-(prop-1-en-2-yl)cyclopent-2-enylidene)hexyl)-4,4,5,5-tetramethyl-1,3,2-dioxaborolane (**4g**)*

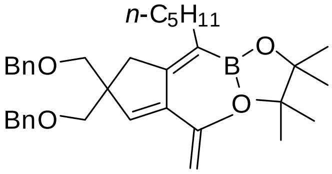

57% isolated yield, liquid. ^{1}H NMR (500 MHz, $CDCl_3$): δ 7.35-7.24 (m, 10H), 5.88 (s, 1H), 4.99 (s, 1H), 4.97 (s, 1H), 4.53 (s, 4H), 3.47 (s, 4H), 2.49 (s, 2H), 2.14 (t, J = 8.0 Hz, 2H), 1.95 (s, 3H), 1.42-1.35 (m, 2H), 1.34-1.28 (m, 4H), 1.28 (s, 12H), 0.90 (t, J = 7.0 Hz, 3H); ^{13}C NMR (125 MHz, $CDCl_3$): δ 150.9, 149.4, 141.1, 138.9, 136.1, 128.2, 127.4, 127.3, 115.4, 83.1, 73.4, 73.2, 51.4, 37.6, 33.9, 32.1, 29.0, 25.2, 24.0, 22.6, 14.1; HRMS (ESI): calc. for $C_{36}H_{49}BNaO_4$ $[M+Na]^+$: 579.3622; found: 579.3630.

General procedure for oxidative carbocyclization/borylation for the formation of vinylallenes 5

Representative procedure B for the synthesis of ***5****. Dimethyl 4-(prop-1-enylidene)-3-(2-(4,4,5,5-tetramethyl-1,3,2-dioxaborolan-2-yl)-propan-2-yl)cyclopent-2-ene-1,1-dicarboxylate (****5a****)*

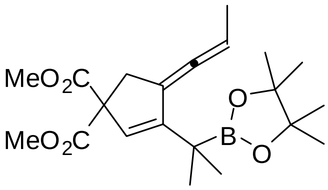

To a mixture of B_2pin_2 (66.2 mg, 0.26 mmol), BQ (24.0 mg, 0.22 mmol), $Pd(OAc)_2$ (1.0 mg, 0.004 mmol), and $BF_3{\cdot}Et_2O$ (6 μL, 0.04 mmol) were added **1a** (52.7 mg, 0.20 mmol) and 1.0 mL of THF at rt. The reaction was stirred at 50 °C for 20 h. After the reaction was complete as monitored by TLC, evaporation and column chromatography on silica gel (pentane/ethyl acetate = 10/1) afforded **5a** (59.6 mg, 77%) as a liquid; ^{1}H NMR (400 MHz, $CDCl_3$): δ 5.55 (d, J = 1.6 Hz, 1H), 5.34-5.23 (m, 1H), 3.716 (s, 3H), 3.715 (s, 3H), 3.19-3.17 (m, 2H), 1.68 (d, J = 7.2 Hz, 3H), 1.18 (s, 12H), 1.17 (s, 3H), 1.13 (s, 3H); ^{13}C NMR (100 MHz, $CDCl_3$): δ 199.1, 171.5, 171.3, 153.8, 122.9, 107.1, 91.2, 83.1, 63.5, 52.7, 36.5, 25.0, 24.7, 24.5, 23.9, 23.8, 14.8; HRMS (ESI): calc. for $C_{21}H_{31}BNaO_6$ $[M+Na]^+$: 413.2106; found: 413.2103.

*Dimethyl 3-(2'-(4,4,5,5-tetramethyl-1,3,2-dioxaborolan-2-yl)propan-2'-yl)-4-vinylidenecyclopent-2-ene-1,1-dicarboxylate (****5b****)*

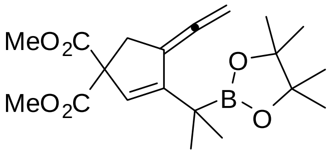

73% isolated yield, liquid. ^{1}H NMR (500 MHz, $CDCl_3$): δ 5.57 (t, J = 1.5 Hz, 1H), 5.02 (t, J = 4.0 Hz, 1H), 5.01 (t, J = 4.0 Hz, 1H), 3.74 (s, 6H), 3.25 (t, J = 4.0 Hz, 2H), 1.20 (s, 12H), 1.19 (s, 6H); ^{13}C NMR (125 MHz, $CDCl_3$): δ 203.1, 171.2, 153.2, 123.0, 107.4, 83.2, 81.0, 63.7, 52.8, 36.4, 24.6, 23.9; HRMS (ESI): calc. for $C_{20}H_{29}BNaO_6$ $[M+Na]^+$: 399.1949; found: 399.1952.

*Dimethyl 3-(2-(4,4,5,5-tetramethyl-1,3,2-dioxaborolan-2-yl)butan-2-yl)-4-vinylidene-cyclopent-2-ene-1,1-dicarboxylate (**5c**)*

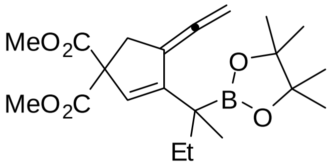

56% isolated yield, liquid. ^{1}H NMR (400 MHz, $CDCl_3$): δ 5.58 (s, 1H), 5.04-4.94 (m, 2H), 3.73 (s, 3H), 3.71 (s, 3H), 3.25 (dt, J_1 = 16.0 Hz, J_2 = 4.0 Hz, 1H), 3.18 (dt, J_1 = 16.0 Hz, J_2 = 4.0 Hz, 1H), 1.83-1.73 (m, 1H), 1.64-1.54 (m, 1H), 1.192 (s, 6H), 1.188 (s, 6H), 1.14 (s, 3H), 0.74 (t, J = 7.2 Hz, 3H); ^{13}C NMR (100 MHz, $CDCl_3$): δ 203.0, 171.3, 171.1, 151.1, 124.8, 107.2, 83.1, 80.8, 63.7, 52.7, 36.5, 28.1, 24.7, 24.6, 19.6, 8.6; HRMS (ESI): calc. for $C_{21}H_{31}BNaO_6$ $[M+Na]^+$: 413.2106; found: 413.2112.

*Dimethyl 4-(hex-1-enylidene)-3-(2-(4,4,5,5-tetramethyl-1,3,2-dioxaborolan-2-yl)-propan-2-yl)cyclopent-2-ene-1,1-dicarboxylate (**5d**)*

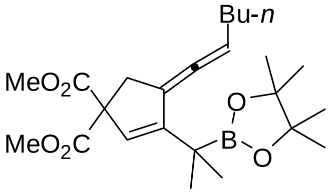

79% isolated yield, liquid. ^{1}H NMR (400 MHz, $CDCl_3$): δ 5.55 (d, J = 1.6 Hz, 1H), 5.32-5.25 (m, 1H), 3.71 (s, 6H), 3.21 (dd, J_1 = 16.0 Hz, J_2 = 3.6 Hz, 1H), 3.14 (dd, J_1 = 16.0 Hz, J_2 = 3.6 Hz, 1H), 2.09-2.01 (m, 2H), 1.42-1.28 (m, 4H), 1.18 (s, 9H), 1.17 (s, 6H), 1.13 (s, 3H), 0.88 (t, J = 7.2 Hz, 3H); ^{13}C NMR (100 MHz, $CDCl_3$): δ 198.0, 171.5, 171.3, 153.9, 122.9, 107.5, 96.6, 83.1, 63.5, 52.7, 36.8, 31.1, 29.2, 24.7, 24.5, 24.0, 23.8, 22.2, 13.8; HRMS (ESI): calc. for $C_{24}H_{37}BNaO_6$ $[M+Na]^+$: 455.2580; found: 455.2582.

*[D_6]-Dimethyl 4-(hex-1-enylidene)-3-(2-(4,4,5,5-tetramethyl-1,3,2-dioxaborolan-2-yl)-propan-2-yl)cyclopent-2-ene-1,1-dicarboxylate ([D_6]-**5d**)*

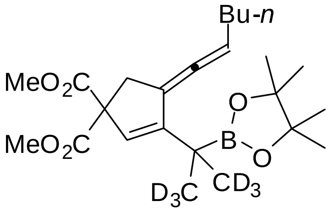

54% isolated yield (Scheme 3c), liquid. ^{1}H NMR (500 MHz, $CDCl_3$): δ 5.56 (s, 1H), 5.32-5.26 (m, 1H), 3.73 (s, 6H), 3.23 (dd, J_1 = 16.0 Hz, J_2 = 3.5 Hz, 1H), 3.15 (dd, J_1 = 16.0 Hz, J_2 = 3.5 Hz, 1H), 2.09-2.01 (m, 2H), 1.42-1.28 (m, 4H), 1.19 (s, 6H), 1.18 (s, 6H), 0.89 (t, J = 7.5 Hz, 3H); ^{13}C NMR (125 MHz, $CDCl_3$): δ 198.0, 171.6, 171.3, 153.9, 122.8, 107.5, 96.6, 83.1, 63.5, 52.7, 36.9, 31.1, 29.3, 24.8, 24.5, 22.2, 13.9; HRMS (ESI): calc. for $C_{24}H_{31}BD_6NaO_6$ $[M+Na]^+$: 461.2956; found: 461.2951.

*Dimethyl 4-(hex-1-enylidene)-3-(2-(4,4,5,5-tetramethyl-1,3,2-dioxaborolan-2-yl)-butan-2-yl)cyclopent-2-ene-1,1-dicarboxylate (**5e**)*

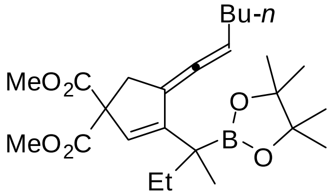

dr ≈ 1/1

77% isolated yield, liquid. ^{1}H NMR (400 MHz, $CDCl_3$): δ 5.61-5.55 (m, 1H), 5.32-5.22 (m, 1H), 3.72-3.68 (m, 6H), 3.25-3.06 (m, 2H), 2.09-1.96 (m, 2H), 1.88-1.76 (m, 0.5H), 1.72-1.62 (m, 1H), 1.51-1.41 (m, 0.5H), 1.40-1.41 (m, 4H), 1.22-1.10 (m, 15H), 0.91-0.82 (m, 3H), 0.81 (t, J = 7.2 Hz, 1.5H), 0.66 (t, J = 7.2 Hz, 1.5H); ^{13}C NMR (100 MHz, $CDCl_3$): δ 198.0, 197.9, 171.5, 171.4, 171.3, 171.2, 152.2, 151.4, 125.0, 124.4, 107.5, 107.2, 96.4, 96.1, 83.1, 83.0, 63.5, 63.4, 52.6, 37.0, 36.9, 31.1, 30.8, 29.2, 29.1, 29.0, 27.4, 25.0, 24.9, 24.7, 24.6, 24.4, 22.2, 20.2, 19.4, 13.8, 9.5, 8.0; HRMS (ESI): calc. for $C_{25}H_{39}BNaO_6$ $[M+Na]^+$: 469.2732; found: 469.2728.

*Dimethyl 4-(hex-1-enylidene)-3-(1-(4,4,5,5-tetramethyl-1,3,2-dioxaborolan-2-yl)-cyclohexyl)cyclopent-2-ene-1,1-dicarboxylate (**5f**)*

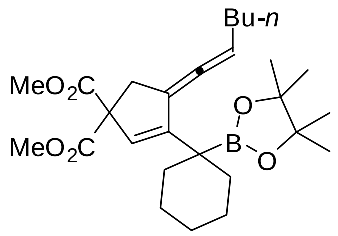

70% isolated yield, liquid. ^1H NMR (400 MHz, $CDCl_3$): δ 5.59 (d, *J* = 1.2 Hz, 1H), 5.30-5.22 (m, 1H), 3.714 (s, 3H), 3.705 (s, 3H), 3.16 (dd, J_1 = 16.0 Hz, J_2 = 3.6 Hz, 1H), 3.09 (dd, J_1 = 16.0 Hz, J_2 = 3.6 Hz, 1H), 2.19-2.01 (m, 4H), 1.70-1.61 (m, 1H), 1.60-1.45 (m, 3H), 1.44-1.25 (m, 8H), 1.19 (s, 6H), 1.18 (s, 6H), 0.89 (t, *J* = 7.2 Hz, 3H); ^{13}C NMR (100 MHz, $CDCl_3$): δ 198.5, 171.5, 171.2, 151.8, 124.2, 106.6, 95.8, 83.1, 63.3, 52.6, 37.2, 32.8, 32.6, 31.0, 29.1, 26.4, 25.0, 24.71, 24.66, 24.5, 24.4, 22.1, 13.9; HRMS (ESI): calc. for $C_{27}H_{41}BNaO_6$ $[M+Na]^+$: 495.2888; found: 495.2877.

*2-(2-(3,3-Bis(benzyloxymethyl)-5-(hex-1-enylidene)cyclopent-1-enyl)propan-2-yl)-4,4,5,5-tetramethyl-1,3,2-dioxaborolane (**5g**)*

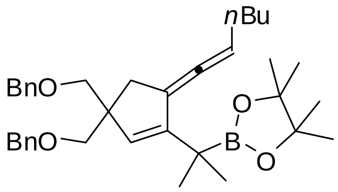

37% isolated yield, liquid. ^1H NMR (500 MHz, $CDCl_3$): δ 7.35-7.24 (m, 10H), 5.51 (d, *J* = 1.5 Hz, 1H), 5.25-5.20 (m, 1H), 4.55 (s, 4H), 3.53-3.42 (m, 4H), 2.54-2.42 (m, 2H), 2.09-2.01 (m, 2H), 1.44-1.30 (m, 4H), 1.21 (s, 6H), 1.20 (s, 6H), 1.18 (s, 3H), 1.14 (s, 3H), 0.91 (t, *J* = 7.0 Hz, 3H); ^{13}C NMR (125 MHz, $CDCl_3$): δ 198.3, 151.0, 139.1, 139.0, 129.2, 128.2, 127.34, 127.29, 127.21, 127.19, 108.9, 95.2, 82.9, 73.8, 73.7, 73.2, 52.0, 37.0, 31.3, 29.5, 24.8, 24.5, 24.1, 24.0, 22.2, 13.9; HRMS (ESI): calc. for $C_{36}H_{49}BNaO_4$ $[M+Na]^+$: 579.3622; found: 579.3623.

Explanation for the formation of 4f' from 4f.

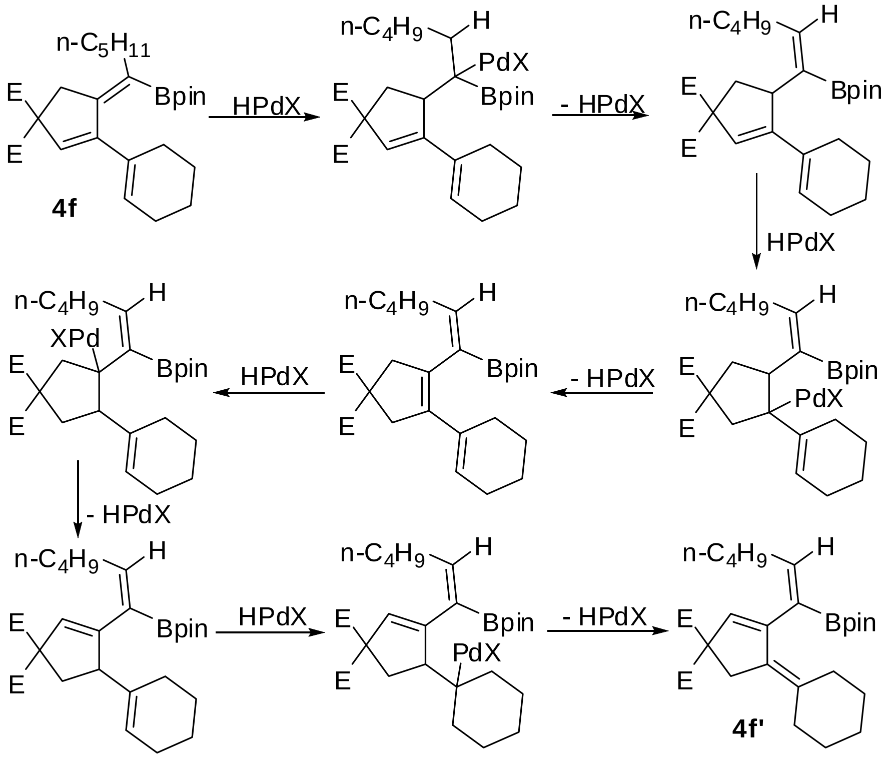

Scheme S1

Application:[3a]

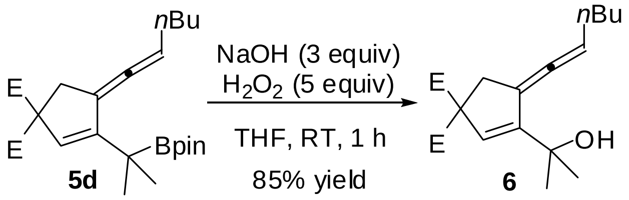

*Dimethyl 4-(hex-1-enylidene)-3-(2-hydroxypropan-2-yl)cyclopent-2-ene-1,1-dicarboxylate (**6**)*[3c]

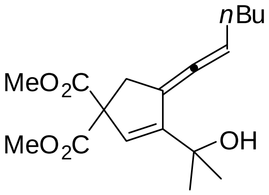

To a solution of **5d** (367.7 mg, 0.85 mmol) in THF (17 mL) were added H_2O_2 (0.48 mL, 30% in H_2O, 4.25 mmol), NaOH aqueous solution (0.85 mL, 3M, 2.55 mmol) sequentially at rt for 1 h. When the reaction was complete as monitored by TLC, the mixture was diluted with Et_2O (20 mL) and H_2O (10 mL). The organic layer was separated and the aqueous layer was extracted with diethyl ether (2 x 20 mL). The combined organic layers were dried over Na_2SO_4. Concentration and column chromatography on silica gel (pentane/ethyl acetate = 3:1) afforded **6** (232.1 mg, 85%): liquid; ^1H NMR (500 MHz, $CDCl_3$): 5.84 (s, 1H), 5.50-5.41 (m, 1H), 3.75 (s, 3H), 3.74 (s, 3H), 3.30-3.20 (m, 2H), 2.14 (s, 1H), 2.13-2.02 (m, 2H), 1.43 (s, 6H), 1.42-1.30 (m, 4H), 0.90 (t, J = 7.0 Hz, 3H).

Kinetic Isotope Effect (KIE) Experiments

1. Determination of Intermolecular Competition KIE of the triene formation.

To a mixture of B_2pin_2 (66.0 mg, 0.26 mmol), BQ (24.0 mg, 0.22 mmol), $Pd(OAc)_2$ (0.9 mg, 0.004 mmol), and $LiOAc{\cdot}2H_2O$ (4.1 mg, 0.04 mmol) were added **1a** (30.6 mg, 0.10 mmol), 0.5 mL of DCE, [D_2]-**1a** (30.8 mg, 0.10 mmol), and 0.5 mL of DCE sequentially in a vial at rt. Then the reaction was stirred at 50 °C for 1 h, quenched with Et_2O (5 mL), and concentrated under reduced pressure. The yields of **4d** and [D_5]-**4d** were analyzed by ^{1}H NMR measurement using anisole as the internal standard (11 μL, 0.1 mmol) and the ratio of **4d**/[D_5]-**4d** was analyzed by HRMS and determined by comparing the abundance of the corresponding [M+Na] and [M+5+Na] peaks (assuming **4d** and [D_5]-**4d** have similar ionization pattern).

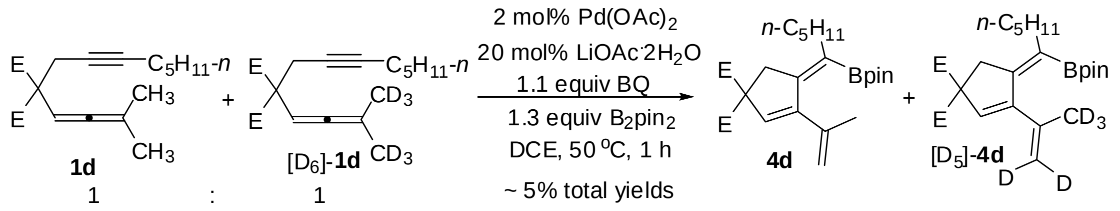

Table S1.

	1st	2nd	average
Abundance of **4d**	173744	102314	
Abundance of [D_5]-**4d**	27420	14671	
Abundance of **1d** in the end of the reaction	625356	441152	
Abundance of [D_6]-**1d** in the end of the reaction	624172	456367	
The ratio (**1d**/[D_6]-**1d**) in the end of the reaction	1.00	0.97	
KIE	6.34	6.97	6.66

Display Report

Analysis Info

Analysis Name	E:\Data2\Youqian\dyq-4-178000001.d	Acquisition Date	2012-12-03 20:54:04
Method	Tune_low_pos.m	Operator	Carin Larsson
Sample Name	dyq-4-178	Instrument / Ser#	micrOTOF 125
Comment			

Acquisition Parameter

Source Type	ESI	Ion Polarity	Positive	Set Nebulizer	0.4 Bar
Focus	Not active			Set Dry Heater	180 °C
Scan Begin	50 m/z	Set Capillary	4000 V	Set Dry Gas	4.0 l/min
Scan End	2000 m/z	Set End Plate Offset	-500 V	Set Divert Valve	Source

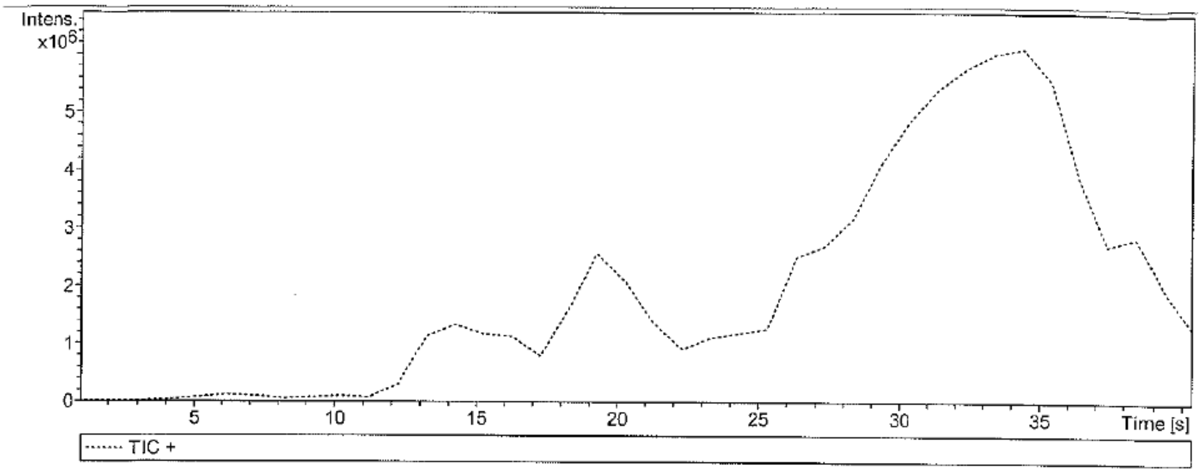

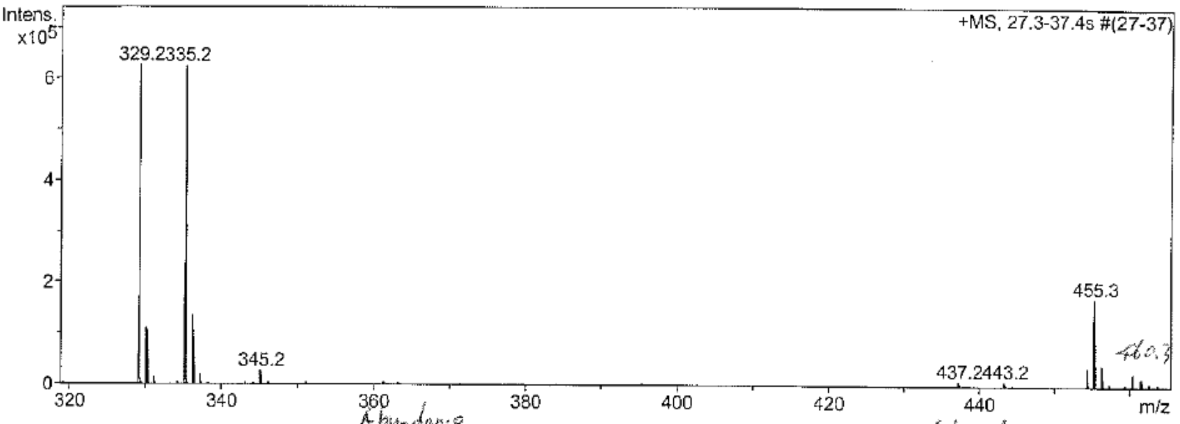

Abundance | Abundance

For mw 329 (1d) = 625356 / mw 335 [D6]-1d = 624172 = 1/1

For mw = 455.3 (4d) = 173744 / Mw = 460 [D5]-4d = 17420 = 6.3/1

2. Determination of Intermolecular Competition KIE of the vinylallene formation.

To a mixture of B_2pin_2 (66.0 mg, 0.26 mmol), BQ (24.0 mg, 0.22 mmol), $Pd(OAc)_2$ (0.9 mg, 0.004 mmol), and $BF_3{\cdot}Et_2O$ (6 μL, 0.04 mmol) were added **1a** (30.6 mg, 0.10 mmol), 0.5 mL of THF, [D_2]-**1a** (30.8 mg, 0.10 mmol), and 0.5 mL of THF sequentially in a vial at rt. Then the reaction was stirred at 50 °C for 1 h, quenched with Et_2O (5 mL), and concentrated under reduced pressure. The yields and the ratio of **5d** and [D_1]-**5d** were analyzed by ^{1}H NMR measurement using anisole as the internal standard (11 μL, 0.1 mmol).

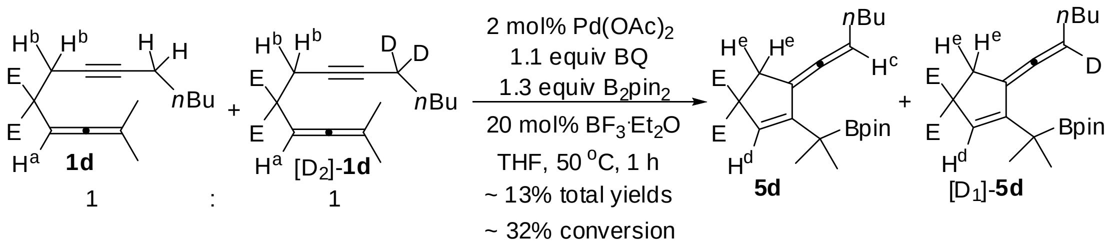

Table S2.

	1st	2nd	3rd	4th	average
Yield of **5d** (%)	7.4	9.6	9.9	9.8	
Total yields of **5d**+[D_1]-**5d** (%)	10.5	15.0	12.9	13.6	13.0
Yield of [D_1]-**5d** (%) (from calculation)	3.1	5.4	3.0	3.8	
Conversion (%)	26	37	33	30	31.5
Ratio between **5d**/[D_1]-**5d**	2.39	1.78	3.30	2.58	2.43
The estimated ratio ([D_2]-**1d**/**1d**) in the end of the reaction	1.12	1.14	1.23	1.09	1.15
KIE	2.53	1.90	3.66	2.81	2.73

Since the ratio of the starting material continuously changes during the reaction, the isotope effect is obtained after a slight correction of this factor. Taking the experiment 1 for example, the ratio (**1d**/[D_2]-**1d**) in the end of the reaction was estimated to be 1:1.12. The yield of (**5d** + [D_1]-**5d**) was 10.5% at a 26% conversion. Here, we assume the 15.5% (26%-10.5%) of the starting materials were converted to by-products without any isotope effect. Then the remaining **1d** should be 50%-(15.5%/2)-7.4% = 34.8% and the remaining [D_2]-**1a** should be 50%-(15.5%/2)-3.1% = 39.1%, respectively. Therefore, the isotope effect calculated from the product ratio and the

change of the starting material ratio is approximately 2.53 (namely, 2.39*[(1+1.12)/2]).

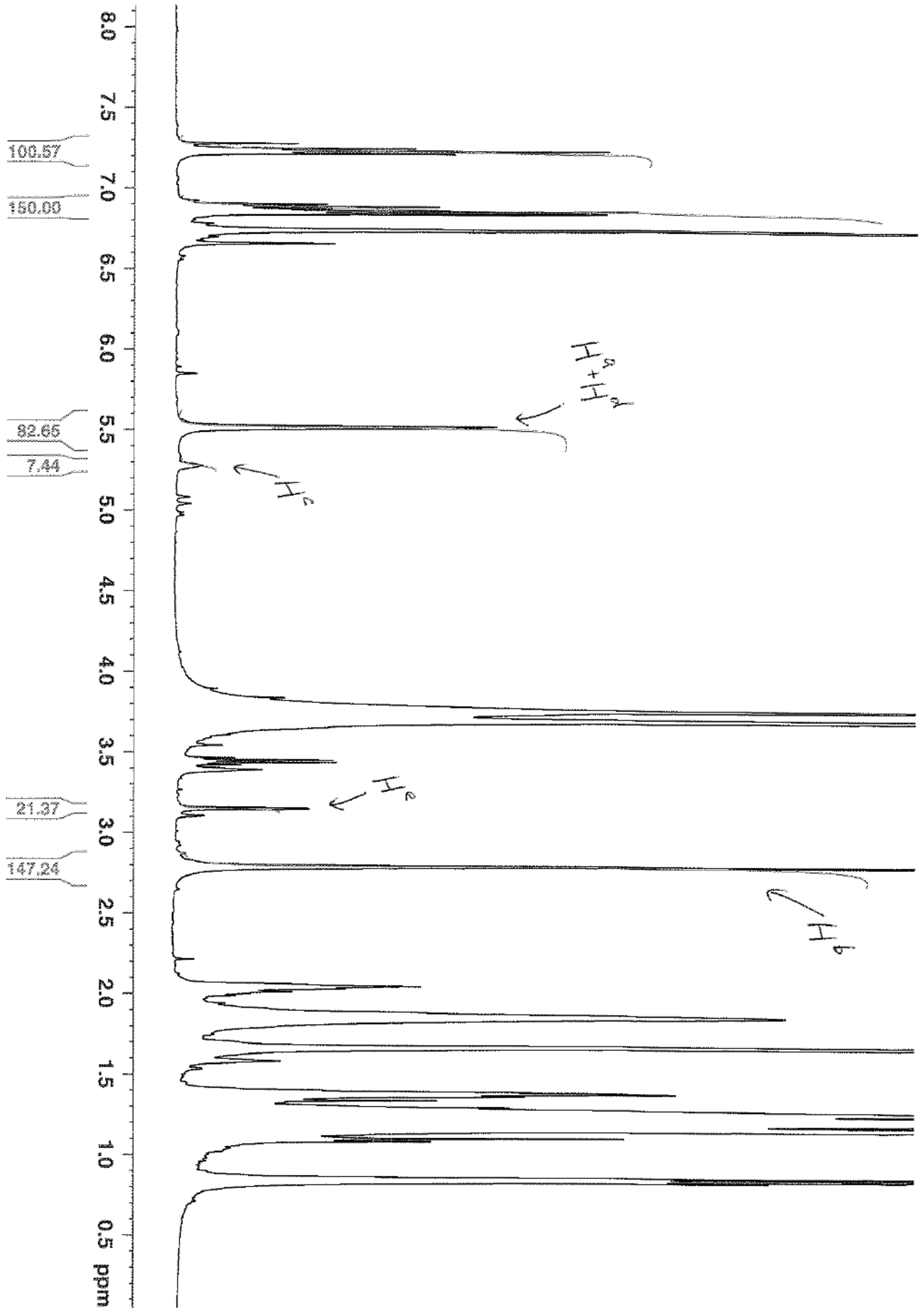

3. Determination of Intramolecular KIE of the vinylallene formation.

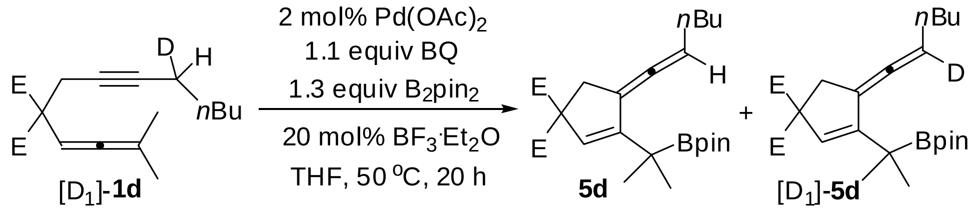

To a mixture of B_2pin_2 (66.0 mg, 0.26 mmol), BQ (24.0 mg, 0.22 mmol), $Pd(OAc)_2$ (0.9 mg, 0.004 mmol), and $BF_3{\cdot}Et_2O$ (6 μL, 0.04 mmol) were added [D_1]-**1d** (61.4 mg, 0.20 mmol) and 1.0 mL of THF sequentially in a vial at rt. Then the reaction was stirred at 50 °C for 20 h, quenched with Et_2O (5 mL), and concentrated under reduced pressure. The yields and the ratio of **5d** and [D_1]-**5d** were analyzed by ^{1}H NMR measurement using anisole as the internal standard (22 μL, 0.2 mmol).

Table S3.

	1st	2nd	3rd	average
Yield of **5d** (%)	12	11	9	
Yield of [D_1]-**5d** (%)	54	55	57	
Total yields (%)	66	66	66	66
KIE	4.50	5.00	6.33	5.28

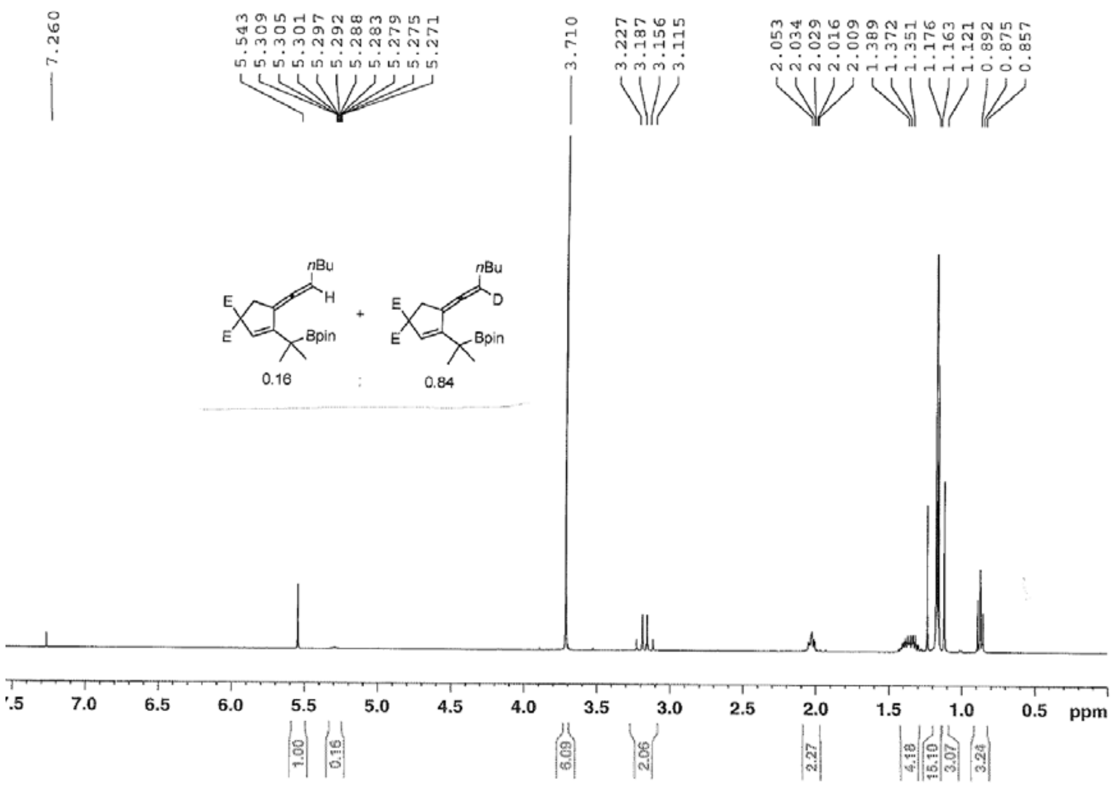

4. Effect of isotope substitution at the alkyne part on product distribution.

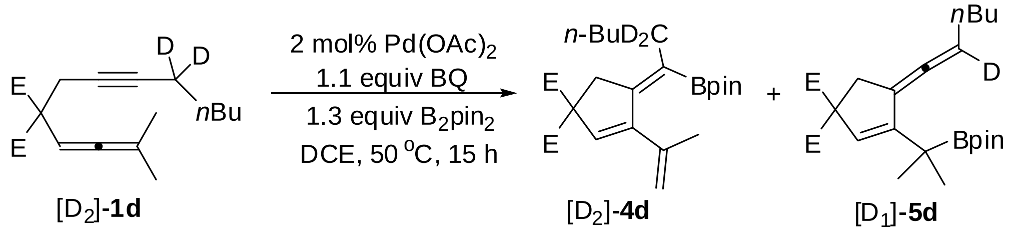

To a mixture of B_2pin_2 (33.0 mg, 0.13 mmol), BQ (12.0 mg, 0.11 mmol), and $Pd(OAc)_2$ (0.5 mg, 0.002 mmol) were added [D_2]-**1d** (30.8 mg, 0.10 mmol) and 0.5 mL of DCE sequentially in a vial at rt. Then the reaction was stirred at 50 ºC for 15 h, quenched with Et_2O (2.5 mL), and concentrated under reduced pressure. The yields and the ratio of [D_2]-**4d** and [D_1]-**5d** were analyzed by ^{1}H NMR measurement using anisole as the internal standard (11 μL, 0.1 mmol).

Table S4.

	1st	2nd	average
Yield of [D_2]-**4d** (%)	73	75	74
Yield of [D_1]-**5d** (%)	6	6	6
Ratio [D_2]-**4d**/[D_1]-**5d**			12.3/1

5. Effect of isotope substitution at allene part on product distribution.

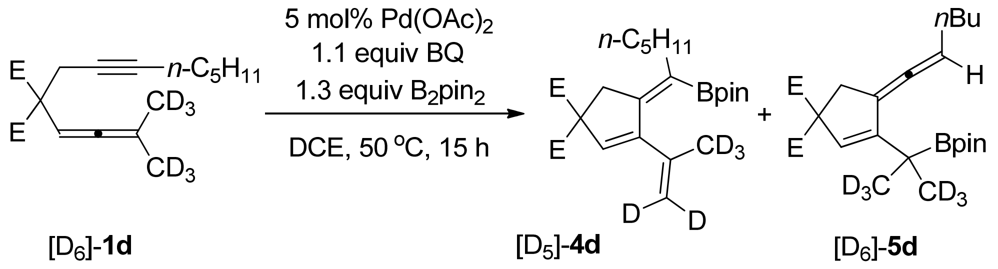

To a mixture of B_2pin_2 (33.0 mg, 0.13 mmol), BQ (12.0 mg, 0.11 mmol), and $Pd(OAc)_2$ (1.2 mg, 0.005 mmol) were added [D_6]-**1d** (31.2 mg, 0.10 mmol) and 0.5 mL of DCE sequentially in a vial at rt. Then the reaction was stirred at 50 ºC for 15 h, quenched with Et_2O (2.5 mL), and concentrated under reduced pressure. The yields and the ratio of [D_5]-**4d** and [D_6]-**5d** were analyzed by ^{1}H NMR measurement using anisole as the internal standard (11 μL, 0.1 mmol).

The product ratio for this experiment was obtained from the integration of the overlapping CH_2 signals at ca. 3.20 ppm. To enhance accuracy of the integration, the ratio was determined as the average of two methods of integrating the signal, which led to [D_5]-**4d**/[D_6]-**5d** is 1/5.0, and the ^{1}H NMR yield is 12% and 60%, respectively.

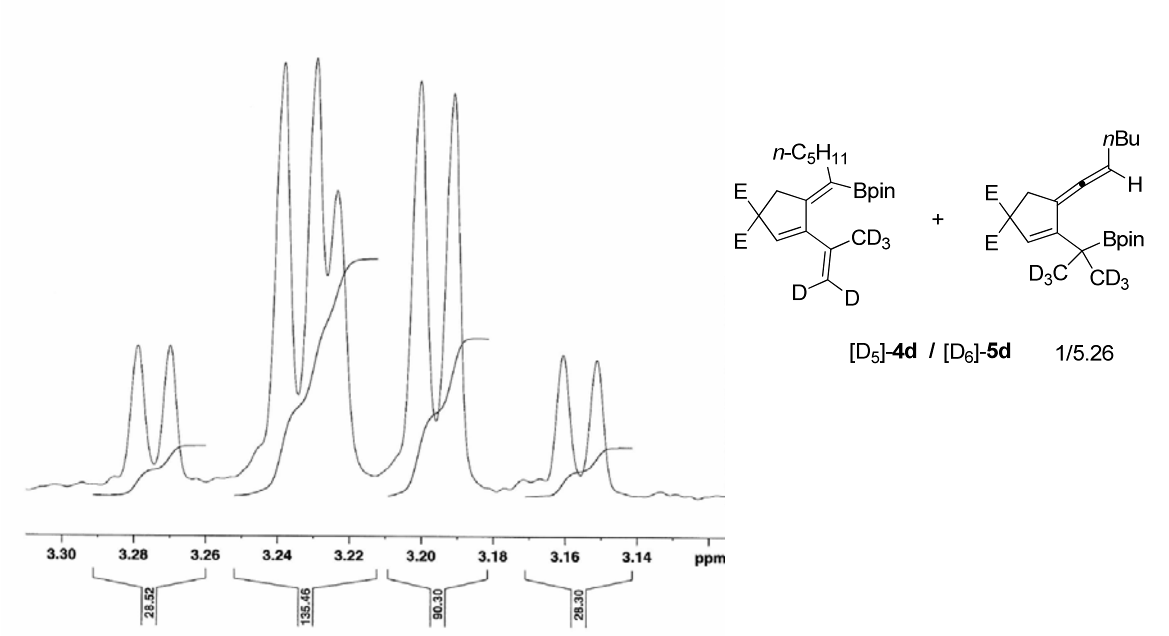

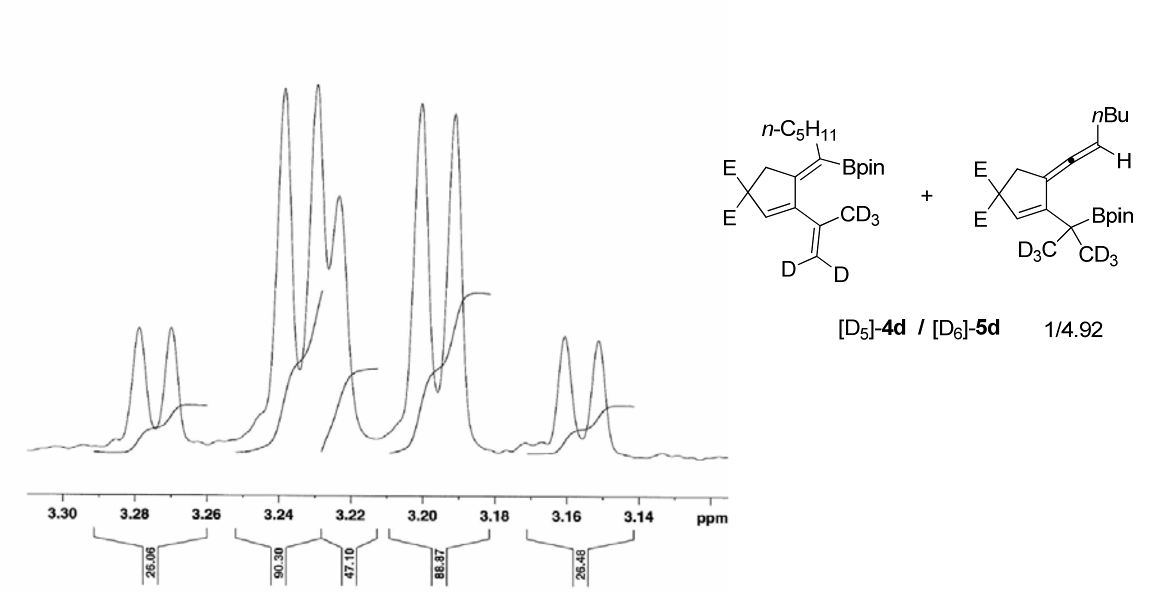

Figure S1.

Alternative mechanism to that proposed in Scheme 5 for oxidative carbocyclization/borylation of allenyne 1

A mechanism involving a pallada(IV)cyclopentene intermediate **F** is also possible, which would generate intermediates **C** and **E** selectively via β-H elimination and subsequent loss of HOAc leading to products **4** and **5**, respectively.

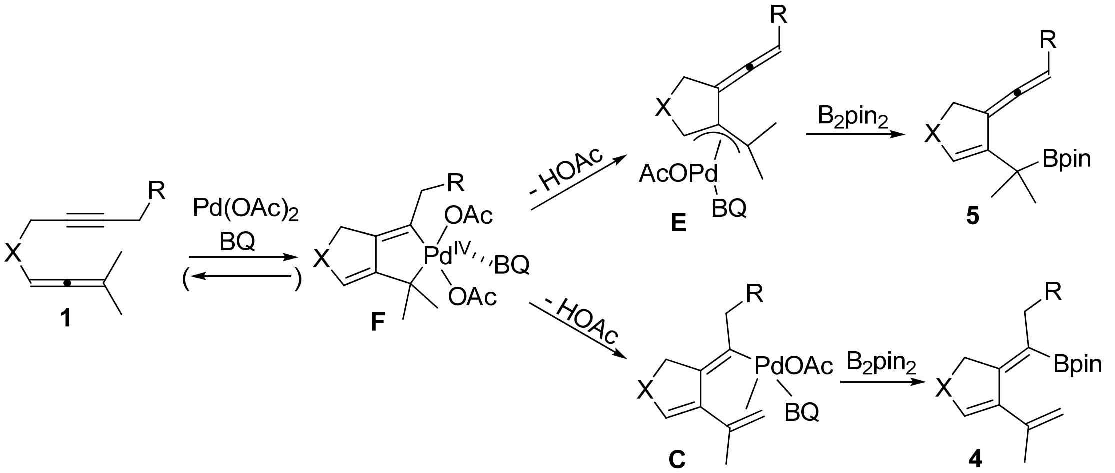

Scheme S2. Proposed alternative mechanism for palladium-catalyzed oxidative selective carbocyclization/borylation of allenynes **1**.

References:

1. a) D. K. Black, S. R. Landor, A. N. Patel, P. F. Whiter, *Tetrahedron Lett*, **1963**, *4*, 483; b) A. K. Å. Persson, E. V. Johnston, J.-E. Bäckvall, *Org. Lett.* **2009**, *11*, 3814.
2. C. Sperger, L. H. S. Strand, A. Fiksdahl, *Tetrahedron* **2010**, *66*, 7749.
3. a) V. Pardo-Rodríguez, J. Marco-Martínez, E. Buñuel, D. J. Cárdenas, *Org. Lett.* **2009**, *11*, 4548; b) Y. Deng, T. Bartholomeyzik, A. K. Å. Persson, J. Sun, J.-E. Bäckvall, *Angew. Chem.* **2012**, *124*, 2757; *Angew. Chem. Int. Ed.* **2012**, *51*, 2703; c) Y. Deng, J.-E. Bäckvall, *Angew. Chem. Int. Ed.* **2013**, DOI: 10.1002/anie.201208718.

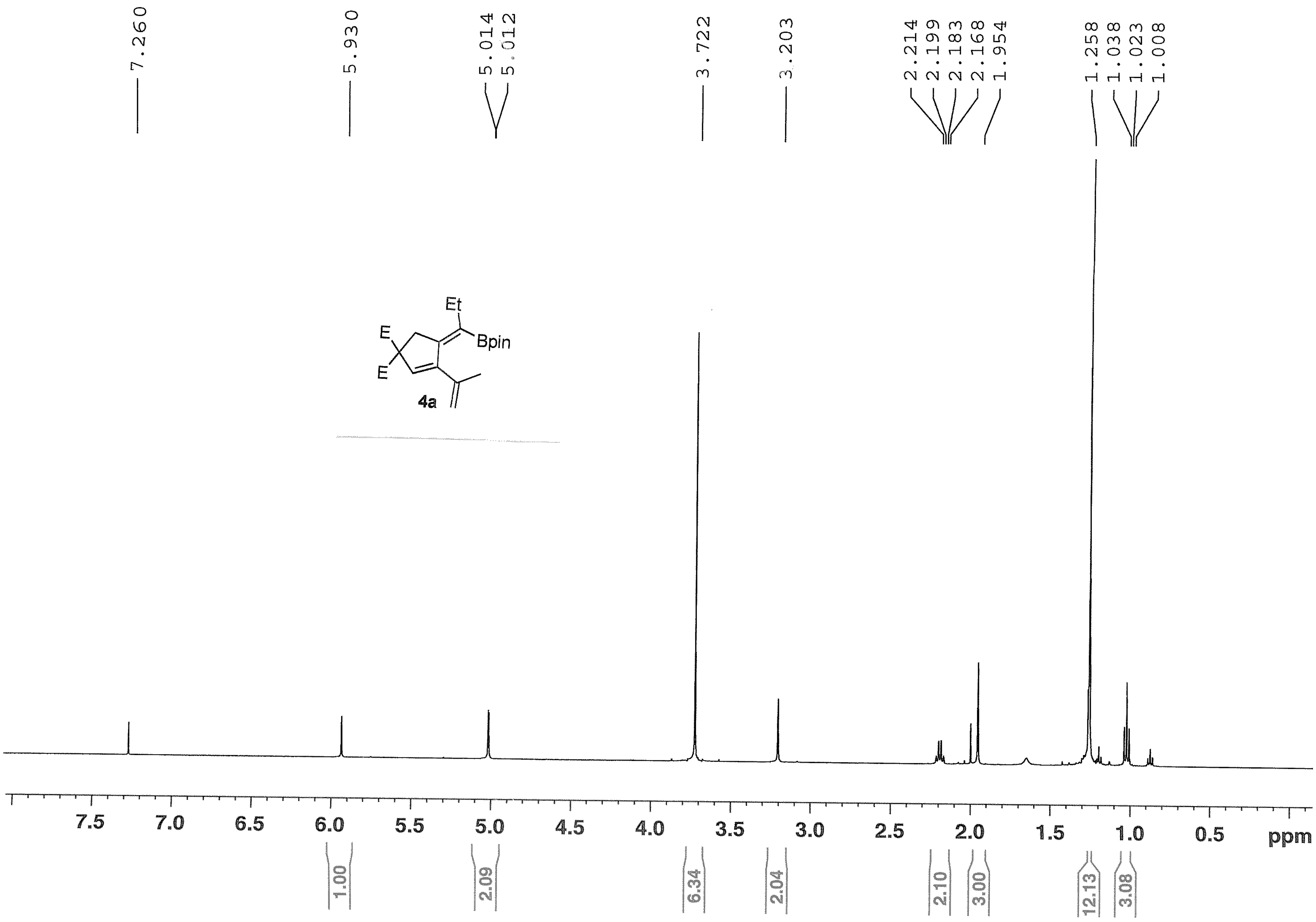
7.260
5.930
5.014
5.012
3.722
3.203
2.214
2.199
2.183
2.168
1.954
1.258
1.038
1.023
1.008
E
E
Et
Bpin
4a
7.5
7.0
6.5
6.0
5.5
5.0
4.5
4.0
3.5
3.0
2.5
2.0
1.5
1.0
0.5
ppm
1.00
2.09
6.34
2.04
2.10
3.00
12.13
3.08

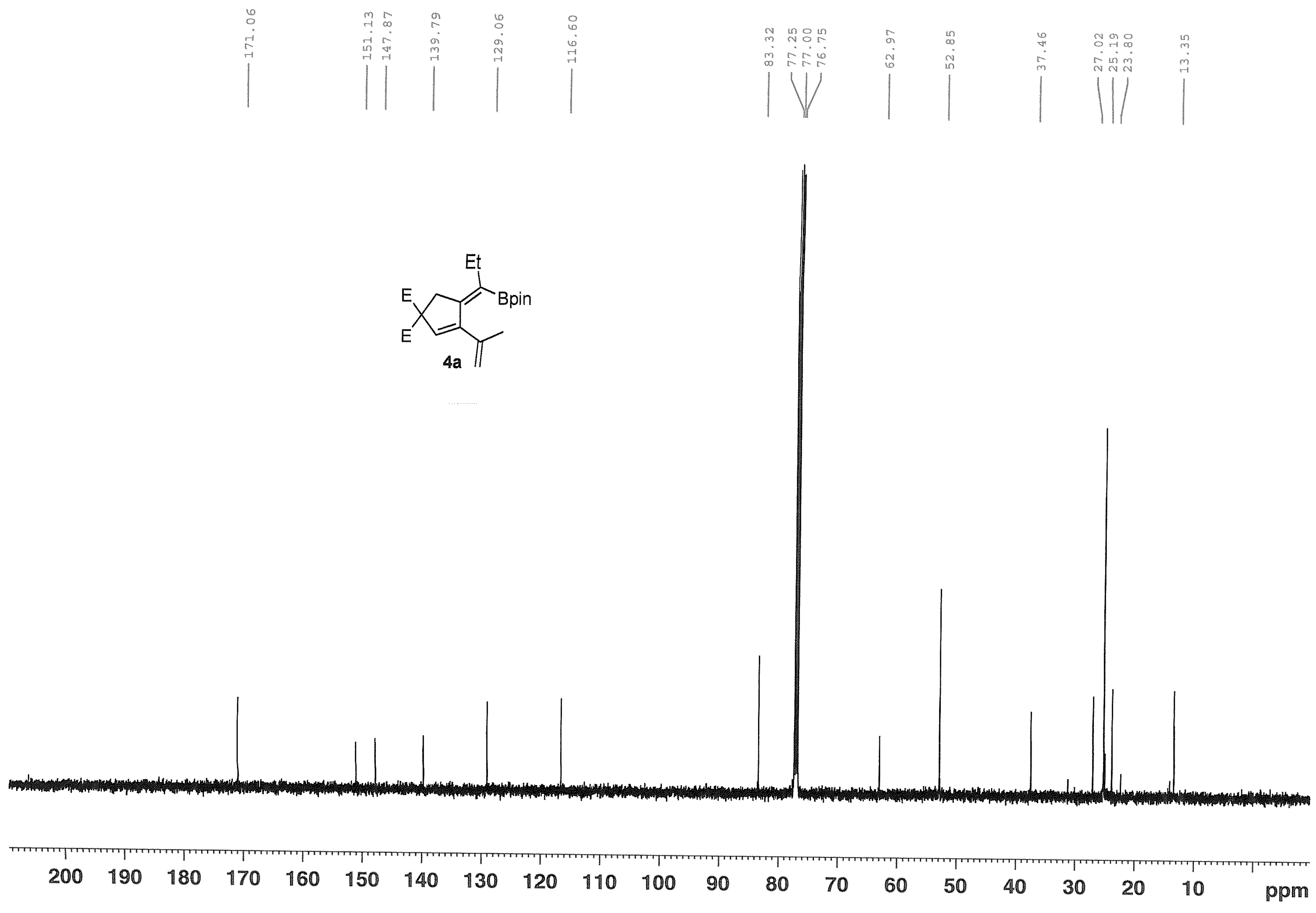
171.06
151.13
147.87
139.79
129.06
116.60
83.32
77.25
77.00
76.75
62.97
52.85
37.46
27.02
25.19
23.80
13.35
Et
Bpin
E
E
4a
200
190
180
170
160
150
140
130
120
110
100
90
80
70
60
50
40
30
20
10
ppm

Mass Spectrum SmartFormula Report

Analysis Info

Analysis Name	E:\Data2\Youqian\dyq-4-155000001.d	Acquisition Date	2012-11-13 14:42:51
Method	Tune_wide_pos.m	Operator	Carin Larsson
Sample Name	dyq-4-155	Instrument / Ser#	micrOTOF 125
Comment			

Acquisition Parameter

Source Type	ESI	Ion Polarity	Positive	Set Nebulizer	0.3 Bar
Focus	Not active			Set Dry Heater	180 °C
Scan Begin	50 m/z	Set Capillary	4500 V	Set Dry Gas	4.0 l/min
Scan End	3000 m/z	Set End Plate Offset	-500 V	Set Divert Valve	Source

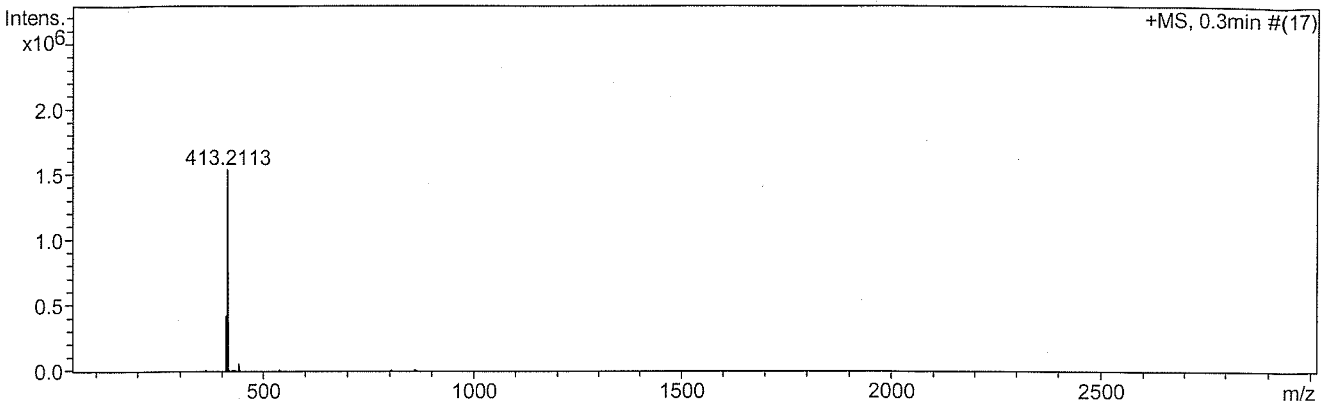

Meas. m/z	#	Formula	Score	m/z	err [mDa]	err [ppm]	mSigma	rdb	e⁻ Conf	N-Rule
413.2113	1	C 21 H 31 B Na O 6	100.00	413.2110	-0.3	-0.8	37.7	6.5	even	ok

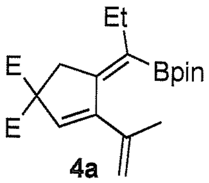

4a

dyq-4-160noe-re 16 1 C:\Bruker\TOPSPIN

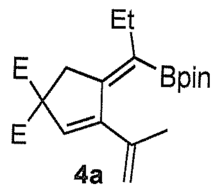

4a

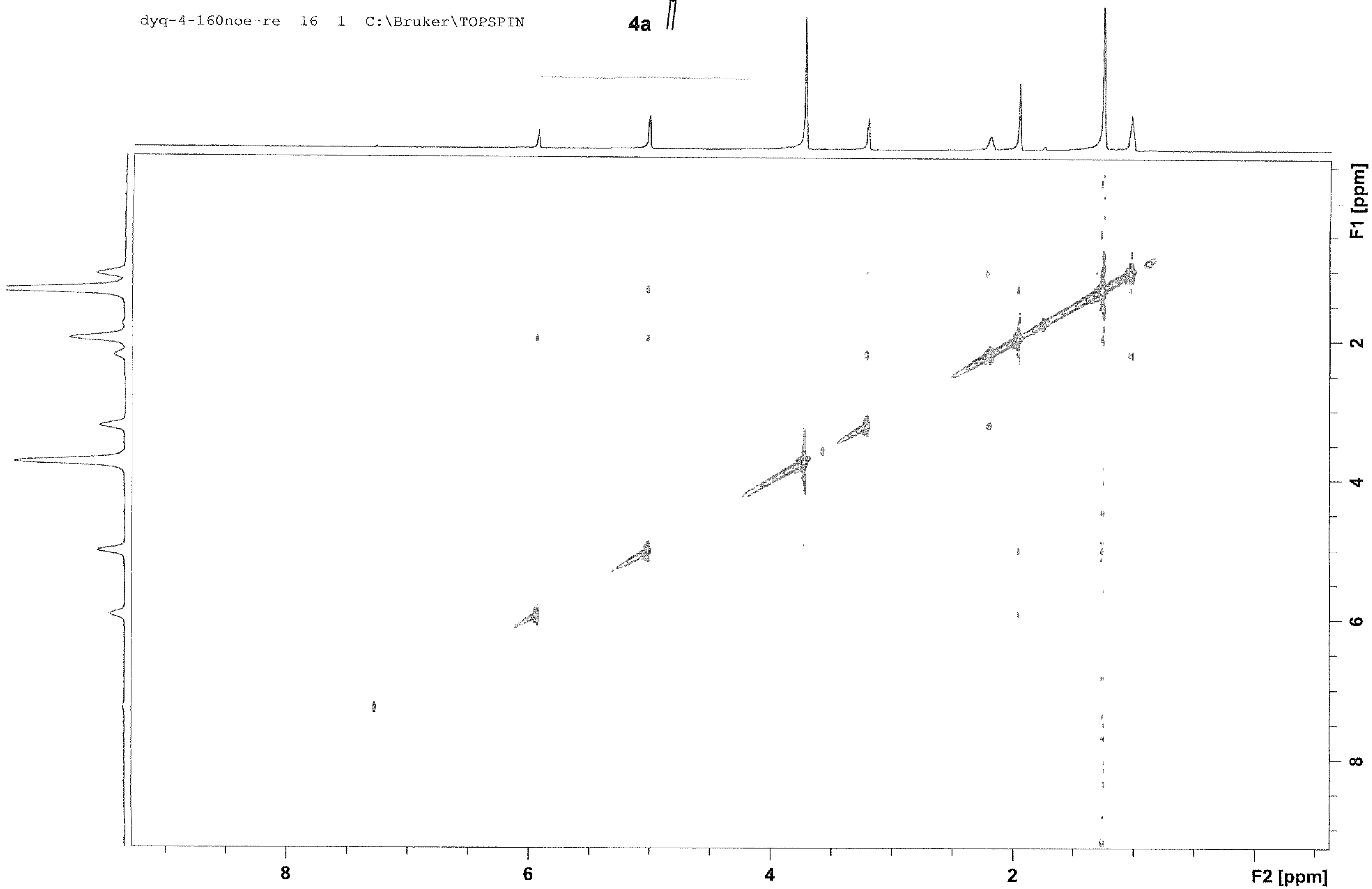

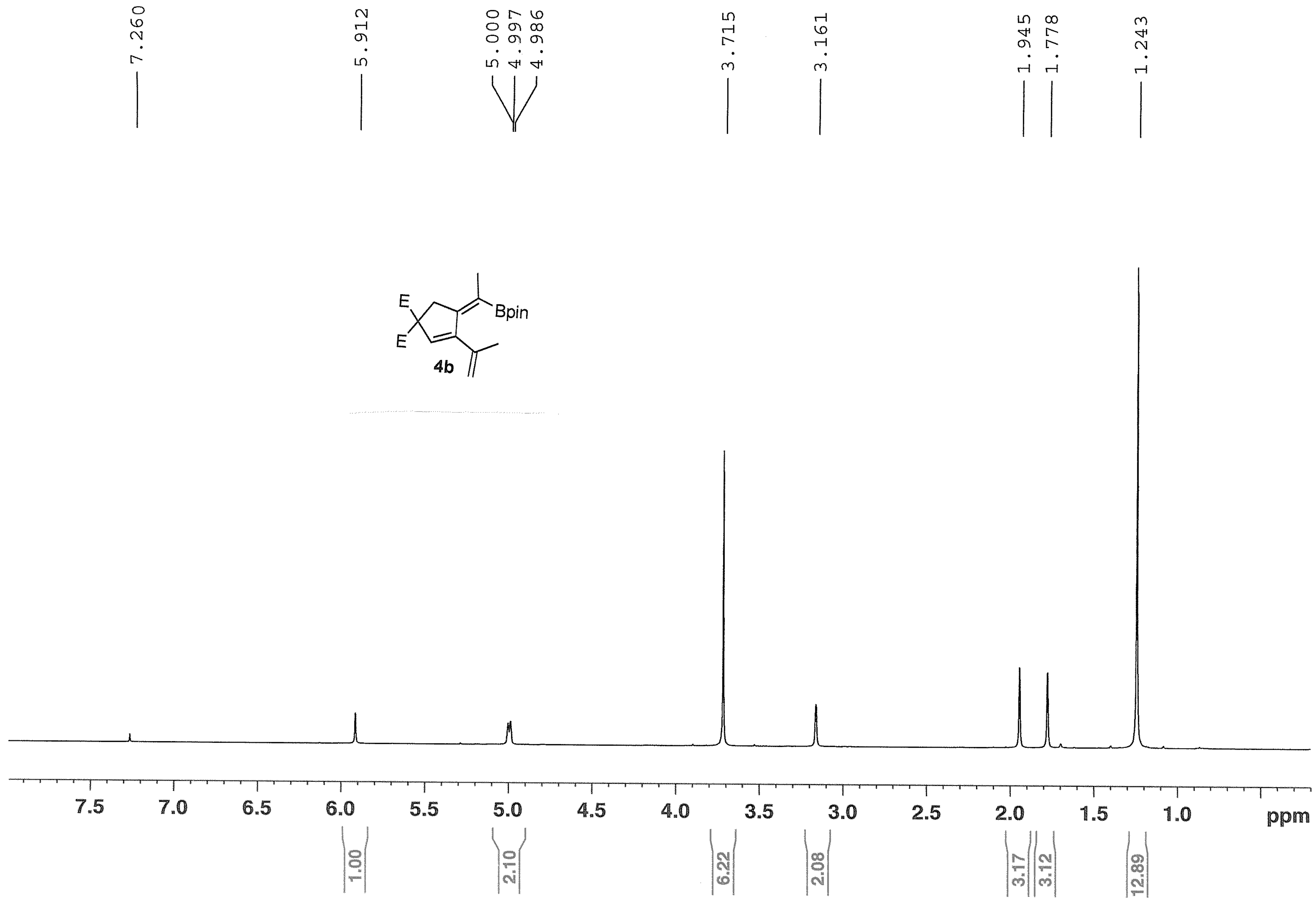
7.260
5.912
5.000
4.997
4.986
3.715
3.161
1.945
1.778
1.243
E
E
Bpin
4b
7.5
7.0
6.5
6.0
5.5
5.0
4.5
4.0
3.5
3.0
2.5
2.0
1.5
1.0
ppm
1.00
2.10
6.22
2.08
3.17
3.12
12.89

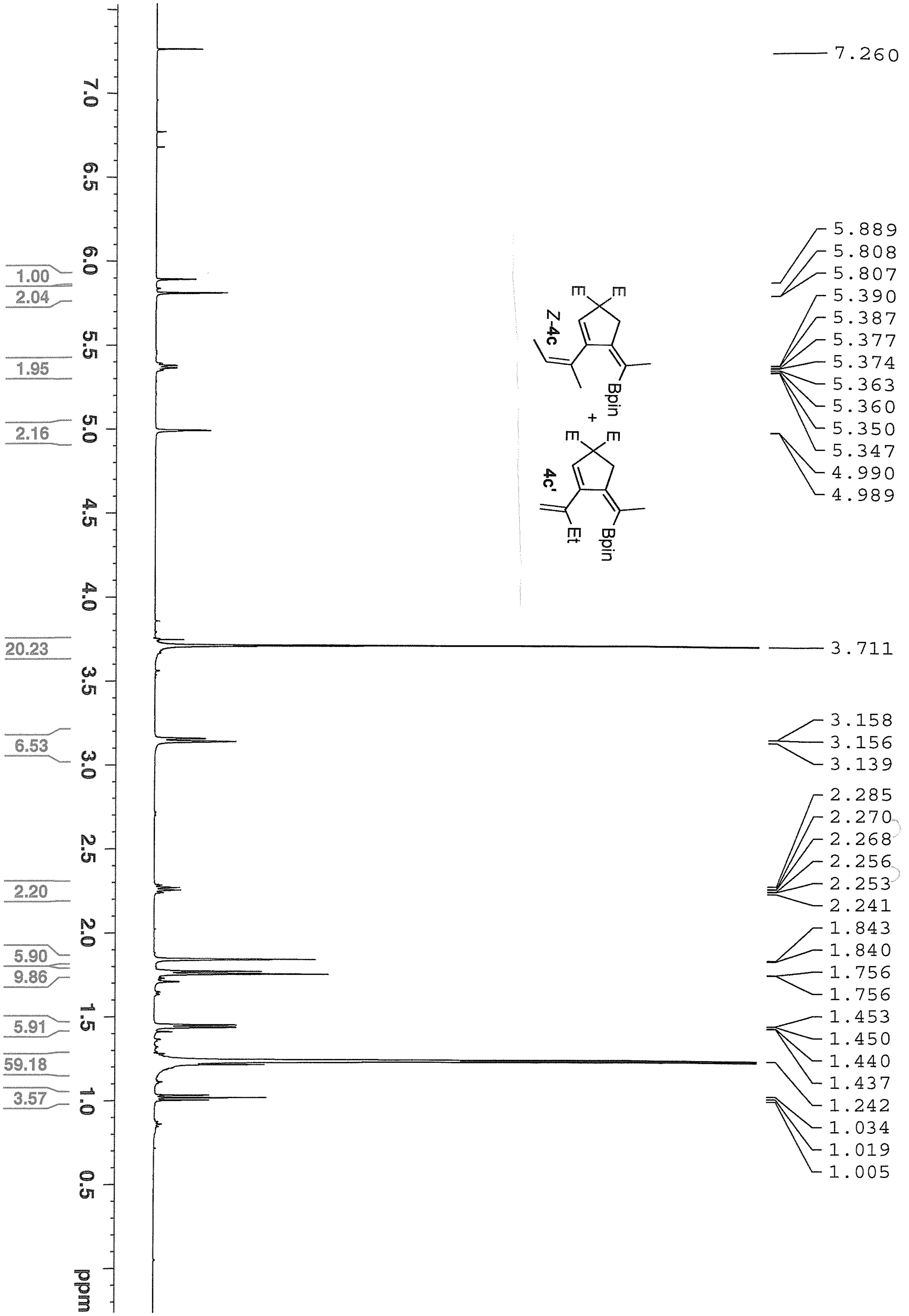

7.260
5.889
5.808
5.807
5.390
5.387
5.377
5.374
5.363
5.360
5.350
5.347
4.990
4.989
3.711
3.158
3.156
3.139
2.285
2.270
2.268
2.256
2.253
2.241
1.843
1.840
1.756
1.756
1.453
1.450
1.440
1.437
1.242
1.034
1.019
1.005
1.00
2.04
1.95
2.16
20.23
6.53
2.20
5.90
9.86
5.91
59.18
3.57
7.0
6.5
6.0
5.5
5.0
4.5
4.0
3.5
3.0
2.5
2.0
1.5
1.0
0.5
ppm
E
E
Bpin
Z-4c
+
4c'
Et

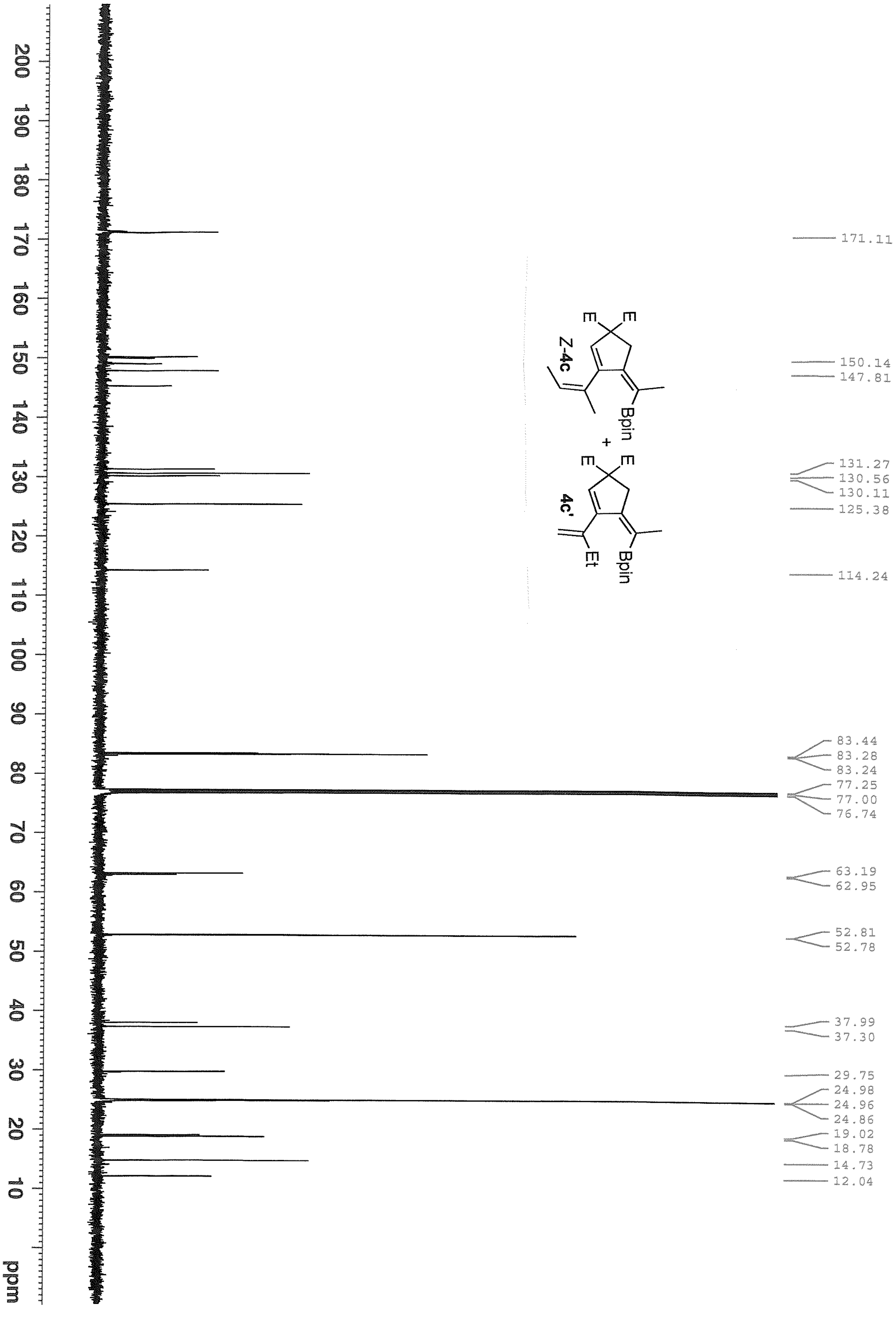
Z-4c
4c'
E
Bpin
Et
+
171.11
150.14
147.81
131.27
130.56
130.11
125.38
114.24
83.44
83.28
83.24
77.25
77.00
76.74
63.19
62.95
52.81
52.78
37.99
37.30
29.75
24.98
24.96
24.86
19.02
18.78
14.73
12.04
200 190 180 170 160 150 140 130 120 110 100 90 80 70 60 50 40 30 20 10
ppm

Mass Spectrum SmartFormula Report

Analysis Info

Analysis Name	E:\Data2\Youqian\Teb-c-45000001.d	Acquisition Date	2012-11-16 18:00:56
Method	Tune_wide_pos.m	Operator	Carin Larsson
Sample Name	Teb-c-45	Instrument / Ser#	micrOTOF 125
Comment			

Acquisition Parameter

Source Type	ESI	Ion Polarity	Positive	Set Nebulizer	0.3 Bar
Focus	Not active			Set Dry Heater	180 °C
Scan Begin	50 m/z	Set Capillary	4500 V	Set Dry Gas	4.0 l/min
Scan End	3000 m/z	Set End Plate Offset	-500 V	Set Divert Valve	Source

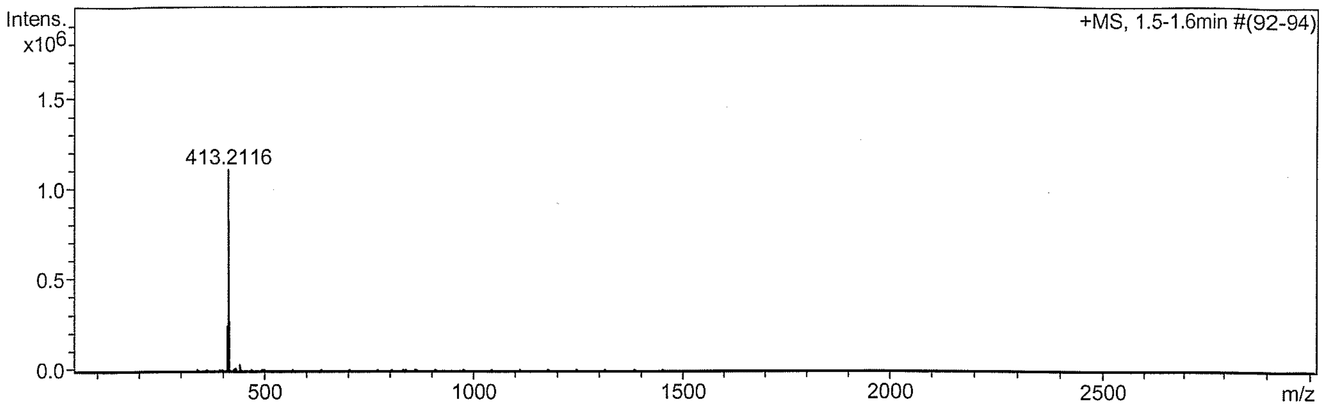

Formula	Meas. m/z	m/z	err [ppm]	Mean err [ppm]
C 21 H 31 B Na O 6	413.2116	413.2110	-1.6	-1.0

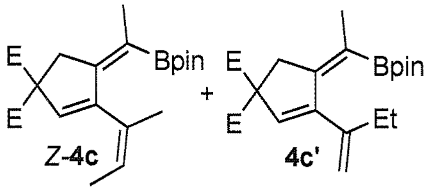

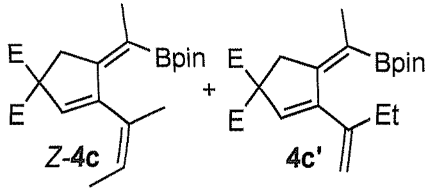

Teb-c-45-1-noe 16 1 C:\Bruker\TOPSPIN dyq

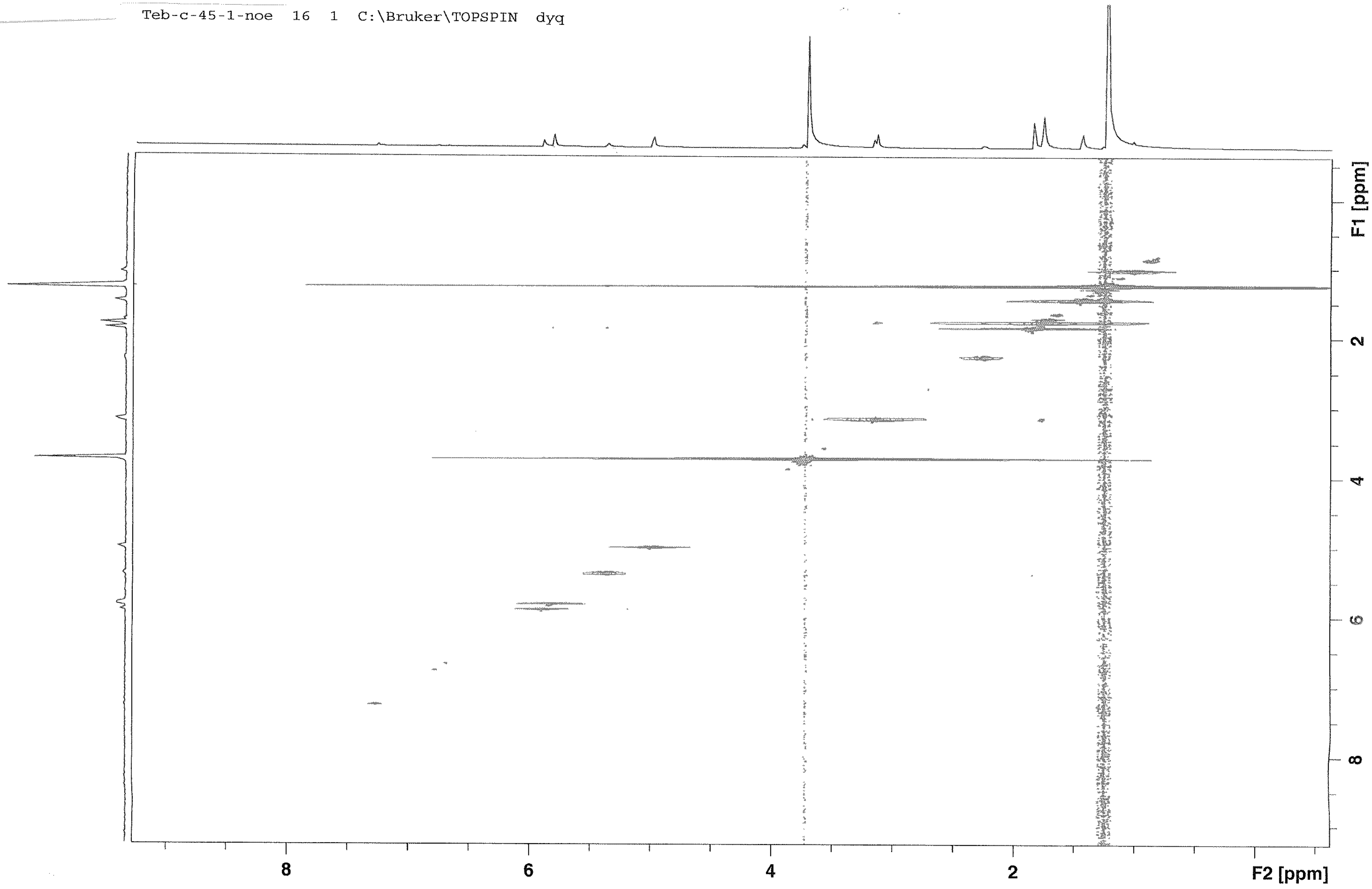

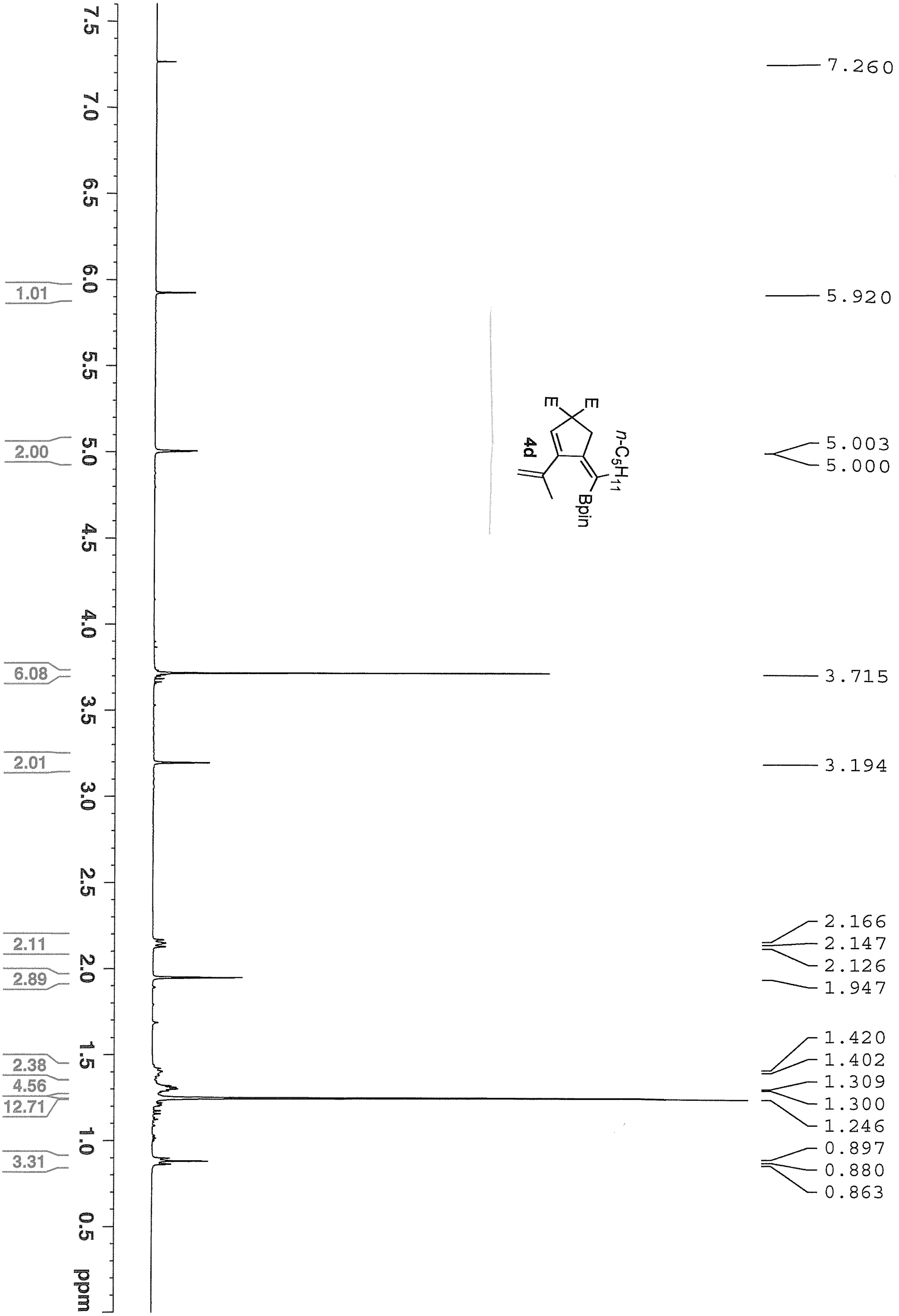

7.260
5.920
5.003
5.000
3.715
3.194
2.166
2.147
2.126
1.947
1.420
1.402
1.309
1.300
1.246
0.897
0.880
0.863
1.01
2.00
6.08
2.01
2.11
2.89
2.38
4.56
12.71
3.31
7.5
7.0
6.5
6.0
5.5
5.0
4.5
4.0
3.5
3.0
2.5
2.0
1.5
1.0
0.5
ppm
E
E
n-C5H11
Bpin
4d

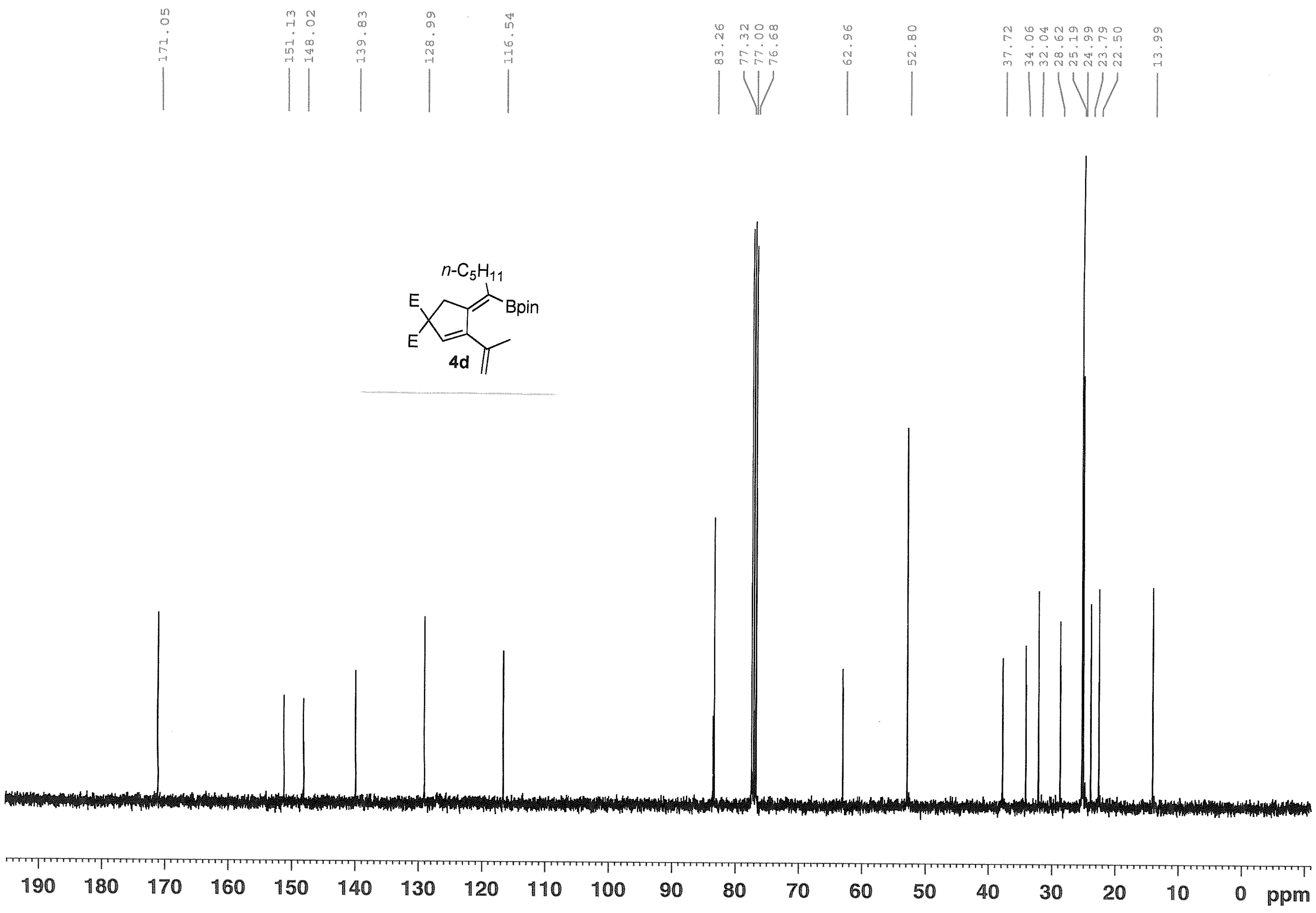
171.05
151.13
148.02
139.83
128.99
116.54
83.26
77.32
77.00
76.68
62.96
52.80
37.72
34.06
32.04
28.62
25.19
24.99
23.79
22.50
13.99
n-C5H11
E
E
Bpin
4d
190
180
170
160
150
140
130
120
110
100
90
80
70
60
50
40
30
20
10
0
ppm

Mass Spectrum SmartFormula Report

Analysis Info

Analysis Name	E:\Data2\Youqian\dyq-4-158-1000003.d	Acquisition Date	2012-11-13 19:03:54
Method	Tune_wide_pos.m	Operator	Carin Larsson
Sample Name	dyq-4-158-1	Instrument / Ser#	micrOTOF 125
Comment			

Acquisition Parameter

Source Type	ESI	Ion Polarity	Positive	Set Nebulizer	0.3 Bar
Focus	Not active			Set Dry Heater	180 °C
Scan Begin	50 m/z	Set Capillary	4500 V	Set Dry Gas	4.0 l/min
Scan End	3000 m/z	Set End Plate Offset	-500 V	Set Divert Valve	Source

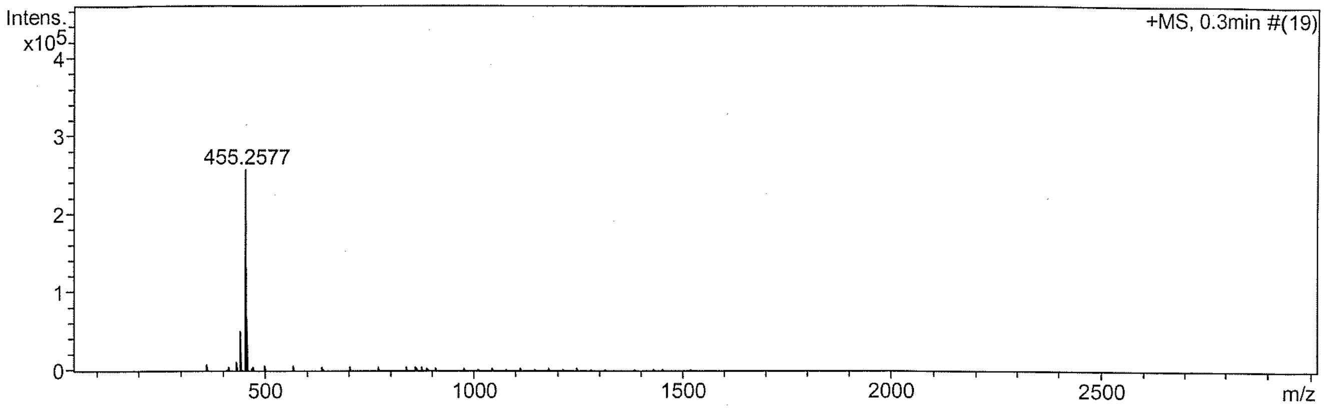

Meas. m/z	#	Formula	Score	m/z	err [mDa]	err [ppm]	mSigma	rdb	e⁻ Conf	N-Rule
455.2577	1	C 24 H 37 B Na O 6	100.00	455.2580	0.2	0.5	14.2	6.5	even	ok

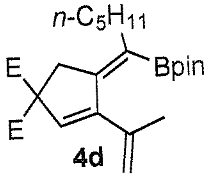

4d

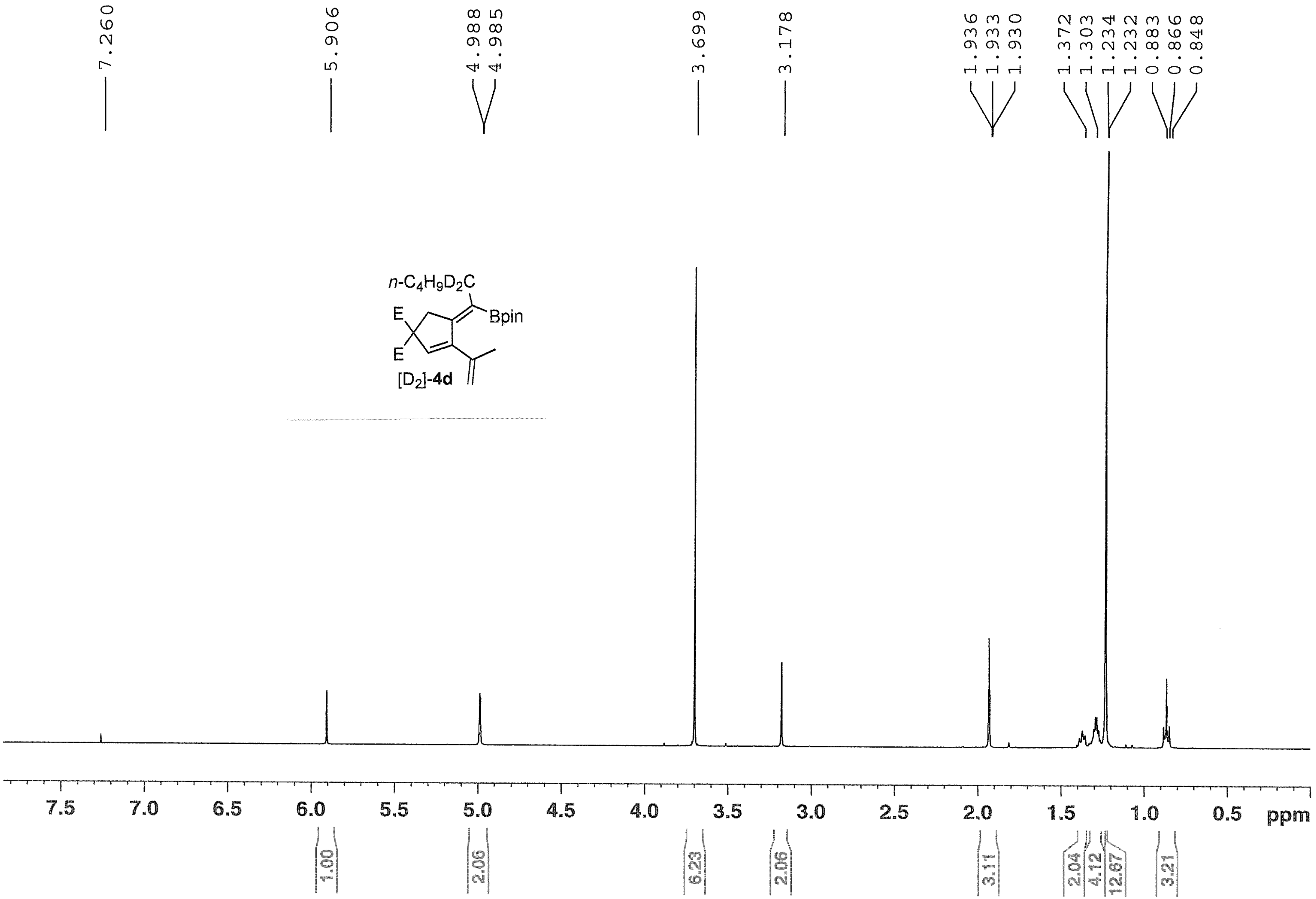
7.260
5.906
4.988
4.985
3.699
3.178
1.936
1.933
1.930
1.372
1.303
1.234
1.232
0.883
0.866
0.848
n-C4H9D2C
E
E
Bpin
[D2]-4d
7.5
7.0
6.5
6.0
5.5
5.0
4.5
4.0
3.5
3.0
2.5
2.0
1.5
1.0
0.5
ppm
1.00
2.06
6.23
2.06
3.11
2.04
4.12
12.67
3.21

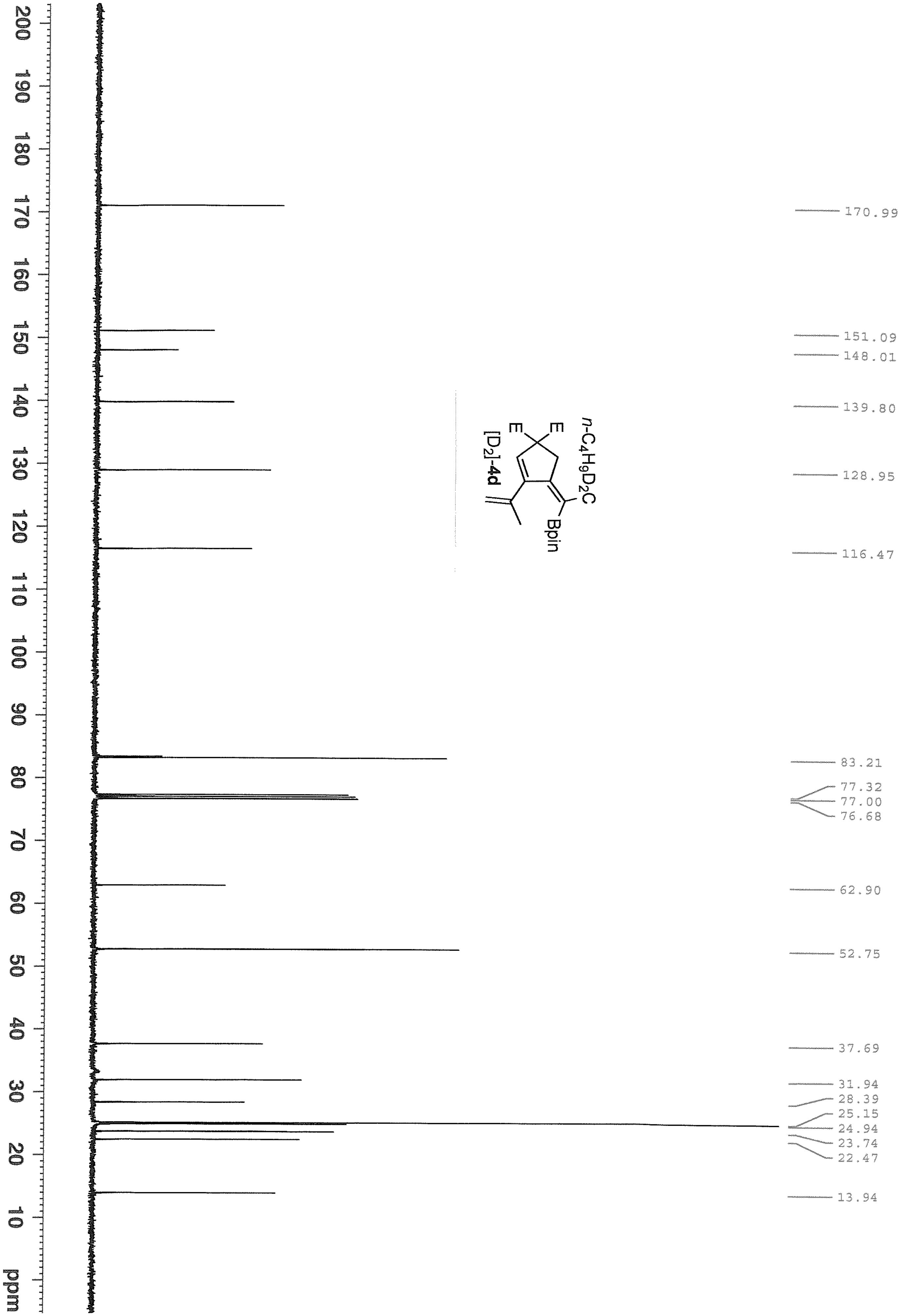

n-C4H9D2C
E
E
Bpin
[D2]-4d
170.99
151.09
148.01
139.80
128.95
116.47
83.21
77.32
77.00
76.68
62.90
52.75
37.69
31.94
28.39
25.15
24.94
23.74
22.47
13.94
200
190
180
170
160
150
140
130
120
110
100
90
80
70
60
50
40
30
20
10
ppm

Mass Spectrum SmartFormula Report

Analysis Info

Analysis Name	E:\Data2\Youqian\dyq-4-182000001.d	Acquisition Date	2012-12-07 18:28:51
Method	Tune_low_pos.m	Operator	Carin Larsson
Sample Name	dyq-4-182	Instrument / Ser#	micrOTOF 125
Comment			

Acquisition Parameter

Source Type	ESI	Ion Polarity	Positive	Set Nebulizer	0.4 Bar
Focus	Not active			Set Dry Heater	180 °C
Scan Begin	50 m/z	Set Capillary	4000 V	Set Dry Gas	4.0 l/min
Scan End	1000 m/z	Set End Plate Offset	-500 V	Set Divert Valve	Source

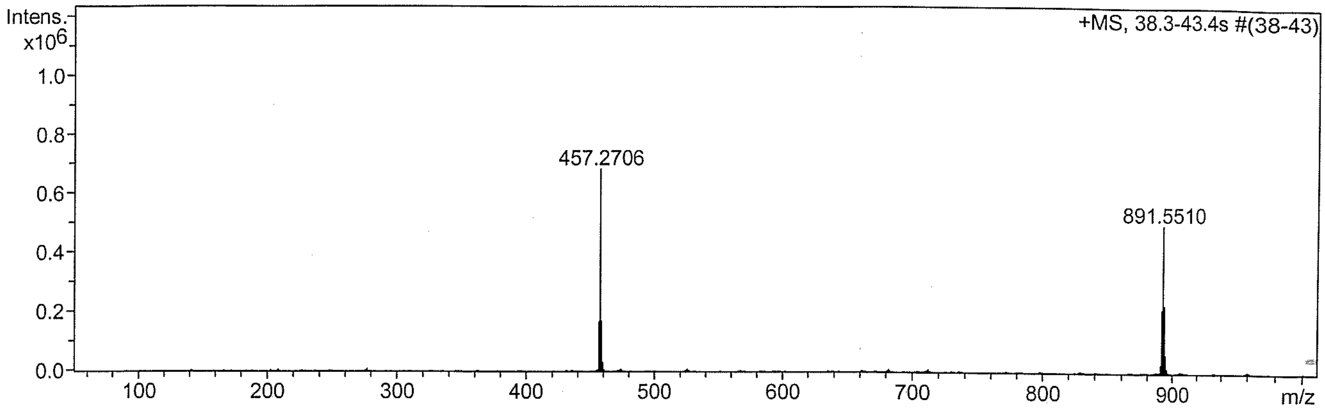

Formula	Meas. m/z	m/z	err [ppm]	Mean err [ppm]
C 24 H 35 B D 2 Na O 6	457.2706	457.2705	-0.2	0.0

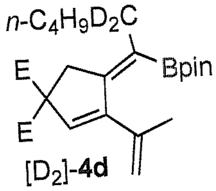

$[D_2]$-**4d**

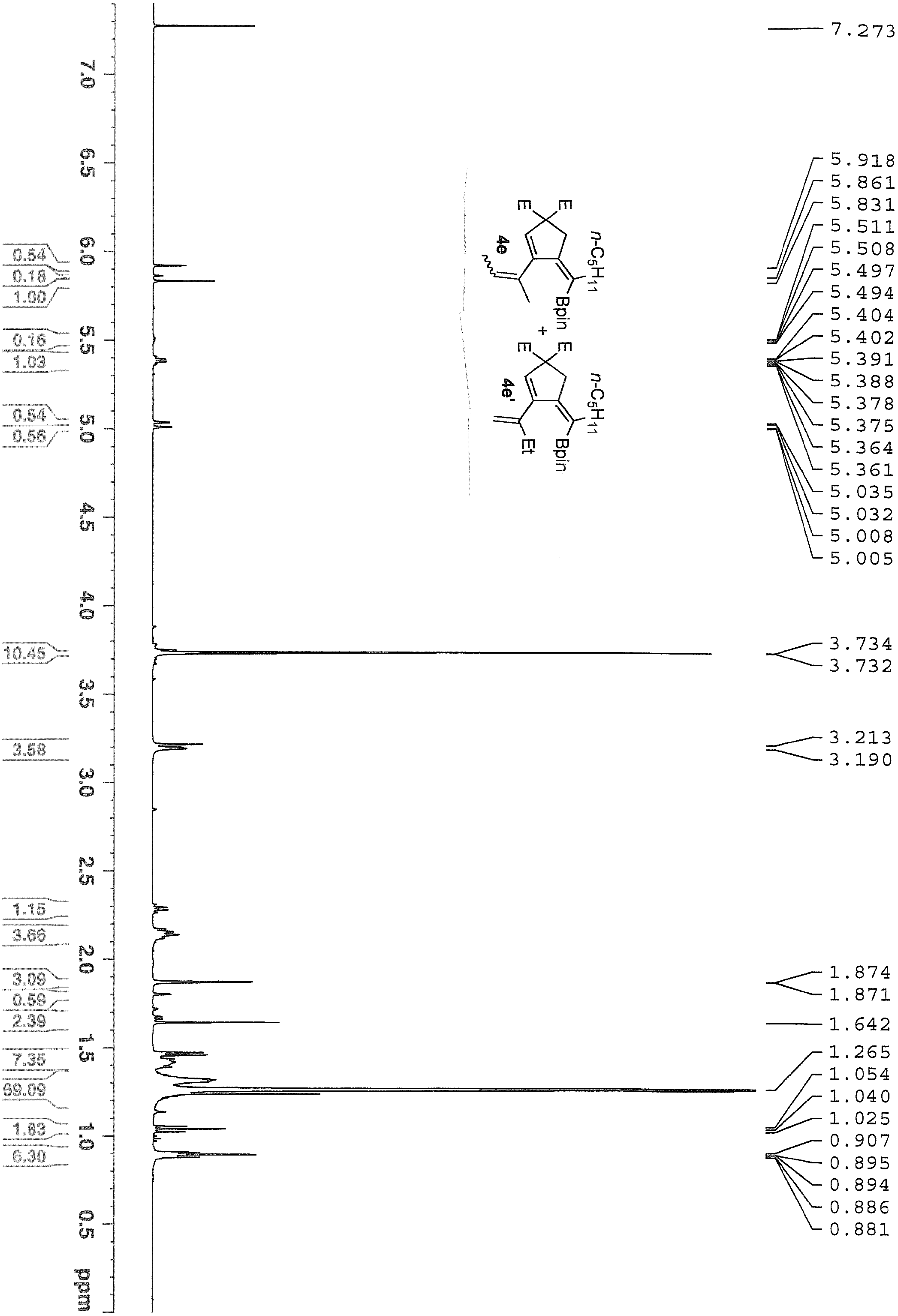

7.273
5.918
5.861
5.831
5.511
5.508
5.497
5.494
5.404
5.402
5.391
5.388
5.378
5.375
5.364
5.361
5.035
5.032
5.008
5.005
3.734
3.732
3.213
3.190
1.874
1.871
1.642
1.265
1.054
1.040
1.025
0.907
0.895
0.894
0.886
0.881
0.54
0.18
1.00
0.16
1.03
0.54
0.56
10.45
3.58
1.15
3.66
3.09
0.59
2.39
7.35
69.09
1.83
6.30
7.0
6.5
6.0
5.5
5.0
4.5
4.0
3.5
3.0
2.5
2.0
1.5
1.0
0.5
ppm
4e
4e'
E
n-C5H11
Bpin
Et
+

Mass Spectrum SmartFormula Report

Analysis Info

Analysis Name	E:\Data2\Youqian\dyq-4-163-1000001.d
Method	Tune_wide_pos.m
Sample Name	dyq-4-163-1
Comment	
Acquisition Date	2012-11-17 14:45:16
Operator	Carin Larsson
Instrument / Ser#	micrOTOF 125

Acquisition Parameter

Source Type	ESI	Ion Polarity	Positive	Set Nebulizer	0.3 Bar
Focus	Not active			Set Dry Heater	180 °C
Scan Begin	50 m/z	Set Capillary	4500 V	Set Dry Gas	4.0 l/min
Scan End	3000 m/z	Set End Plate Offset	-500 V	Set Divert Valve	Source

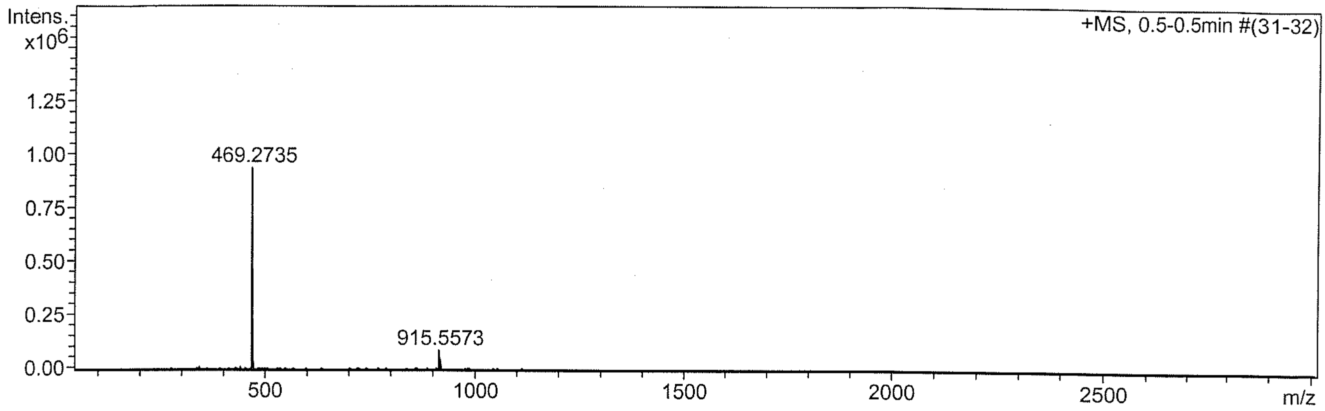

Formula	Meas. m/z	m/z	err [ppm]	Mean err [ppm]
C 25 H 39 B Na O 6	469.2735	469.2736	0.3	0.4

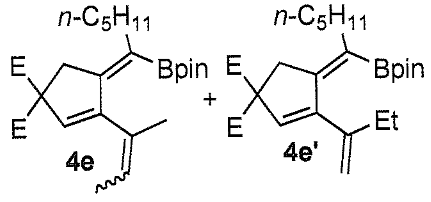

4e + **4e'**

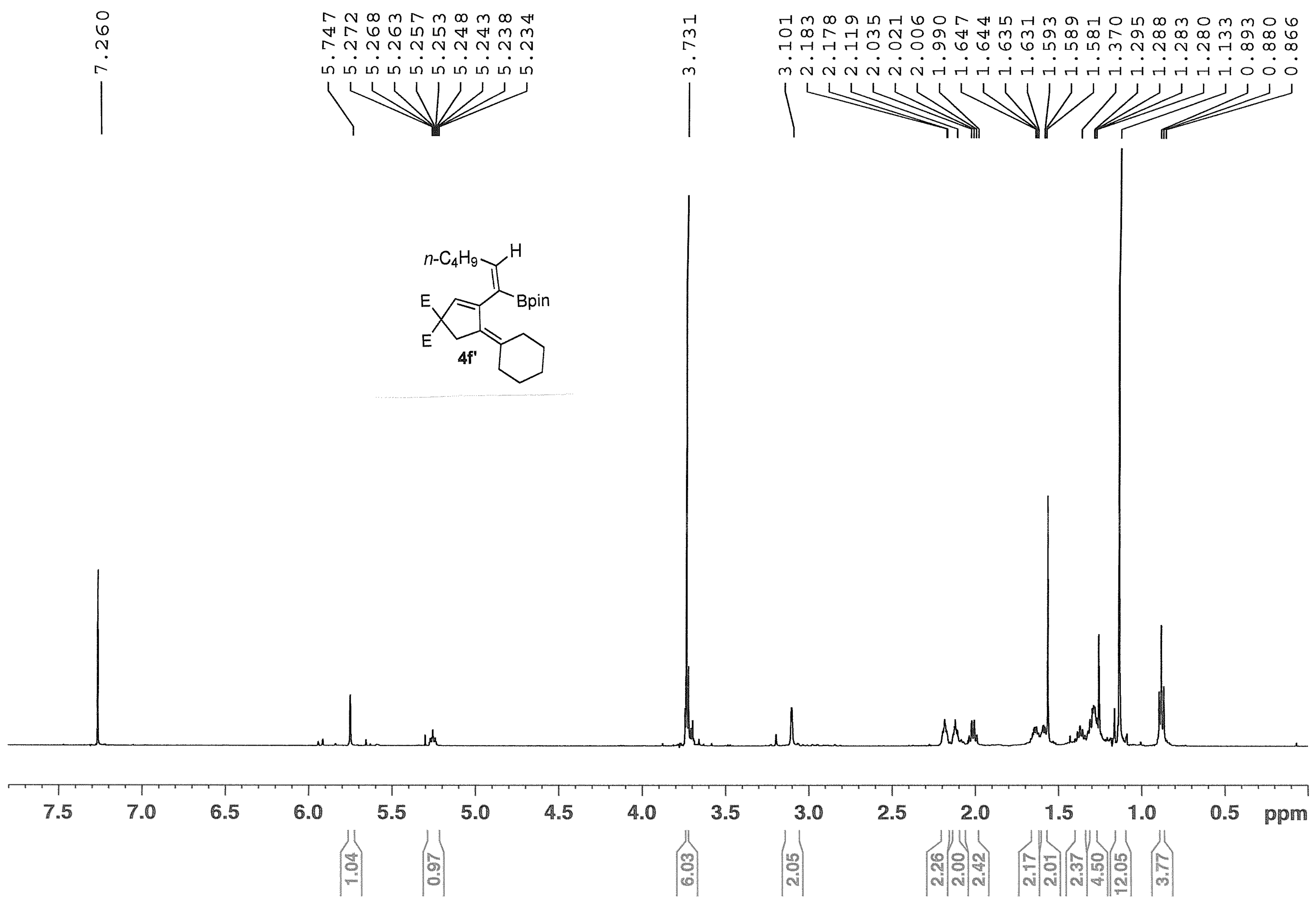
7.260
5.747
5.272
5.268
5.263
5.257
5.253
5.248
5.243
5.238
5.234
3.731
3.101
2.183
2.178
2.119
2.035
2.021
2.006
1.990
1.647
1.644
1.635
1.631
1.593
1.589
1.581
1.370
1.295
1.288
1.283
1.280
1.133
0.893
0.880
0.866
n-C4H9
H
Bpin
E
E
4f'
7.5
7.0
6.5
6.0
5.5
5.0
4.5
4.0
3.5
3.0
2.5
2.0
1.5
1.0
0.5
ppm
1.04
0.97
6.03
2.05
2.26
2.00
2.42
2.17
2.01
2.37
4.50
12.05
3.77

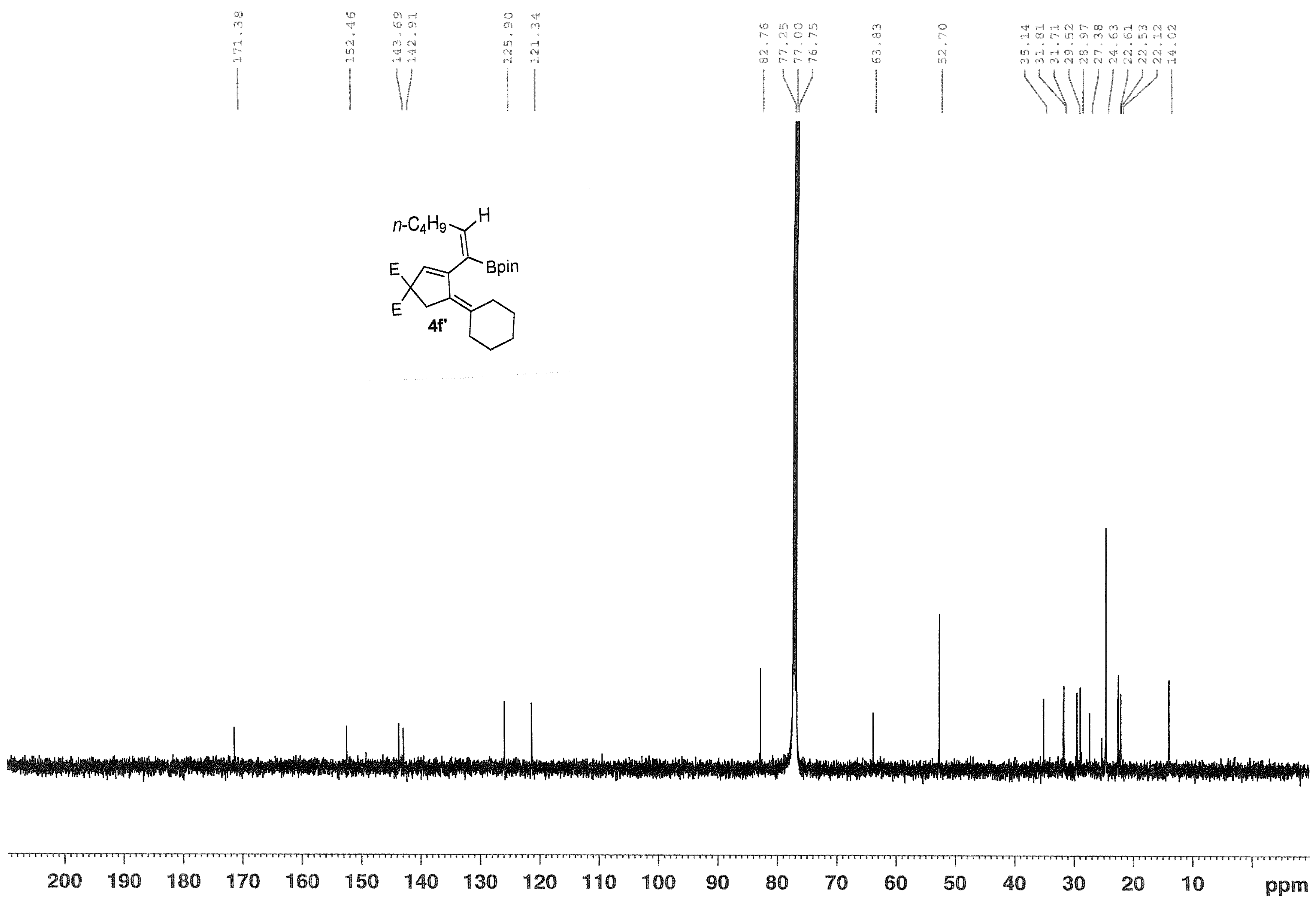
171.38
152.46
143.69
142.91
125.90
121.34
82.76
77.25
77.00
76.75
63.83
52.70
35.14
31.81
31.71
29.52
28.97
27.38
24.63
22.61
22.53
22.12
14.02
n-C4H9
H
Bpin
E
E
4f'
200
190
180
170
160
150
140
130
120
110
100
90
80
70
60
50
40
30
20
10
ppm

Mass Spectrum SmartFormula Report

Analysis Info

Analysis Name	E:\Data2\Youqian\dyq-4-162-1000001.d	Acquisition Date	2012-11-16 14:00:49
Method	Tune_wide_pos.m	Operator	Carin Larsson
Sample Name	dyq-4-162-1	Instrument / Ser#	micrOTOF 125
Comment			

Acquisition Parameter

Source Type	ESI	Ion Polarity	Positive	Set Nebulizer	0.3 Bar
Focus	Not active			Set Dry Heater	180 °C
Scan Begin	50 m/z	Set Capillary	4500 V	Set Dry Gas	4.0 l/min
Scan End	3000 m/z	Set End Plate Offset	-500 V	Set Divert Valve	Source

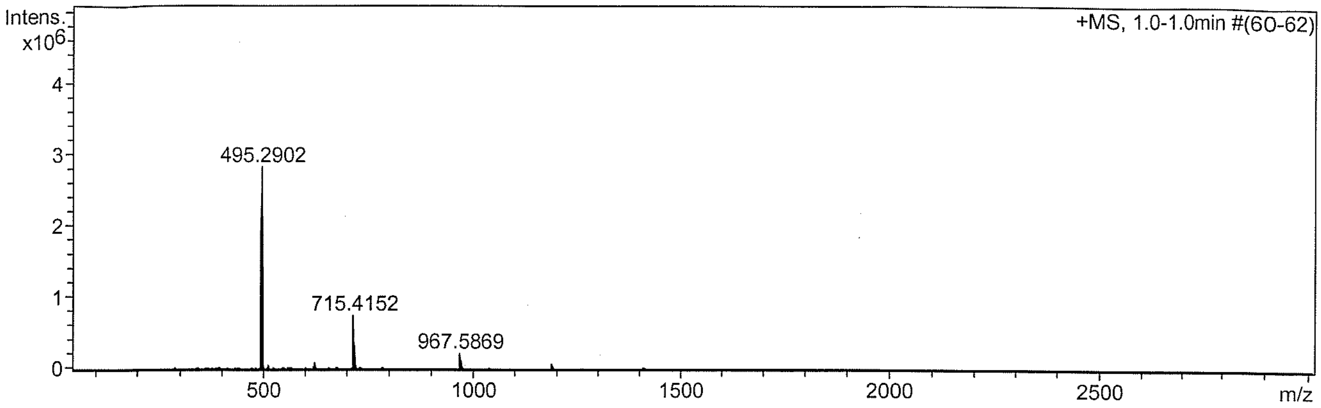

Formula	Meas. m/z	m/z	err [ppm]	Mean err [ppm]
C 27 H 41 B Na O 6	495.2902	495.2893	-1.9	-2.1

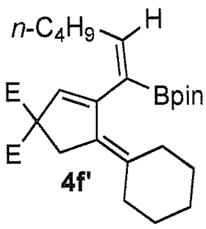

4f'

dyq-4-162-1noe 16 1 C:\Bruker\TOP?

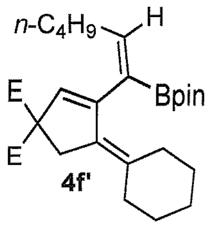

4f'

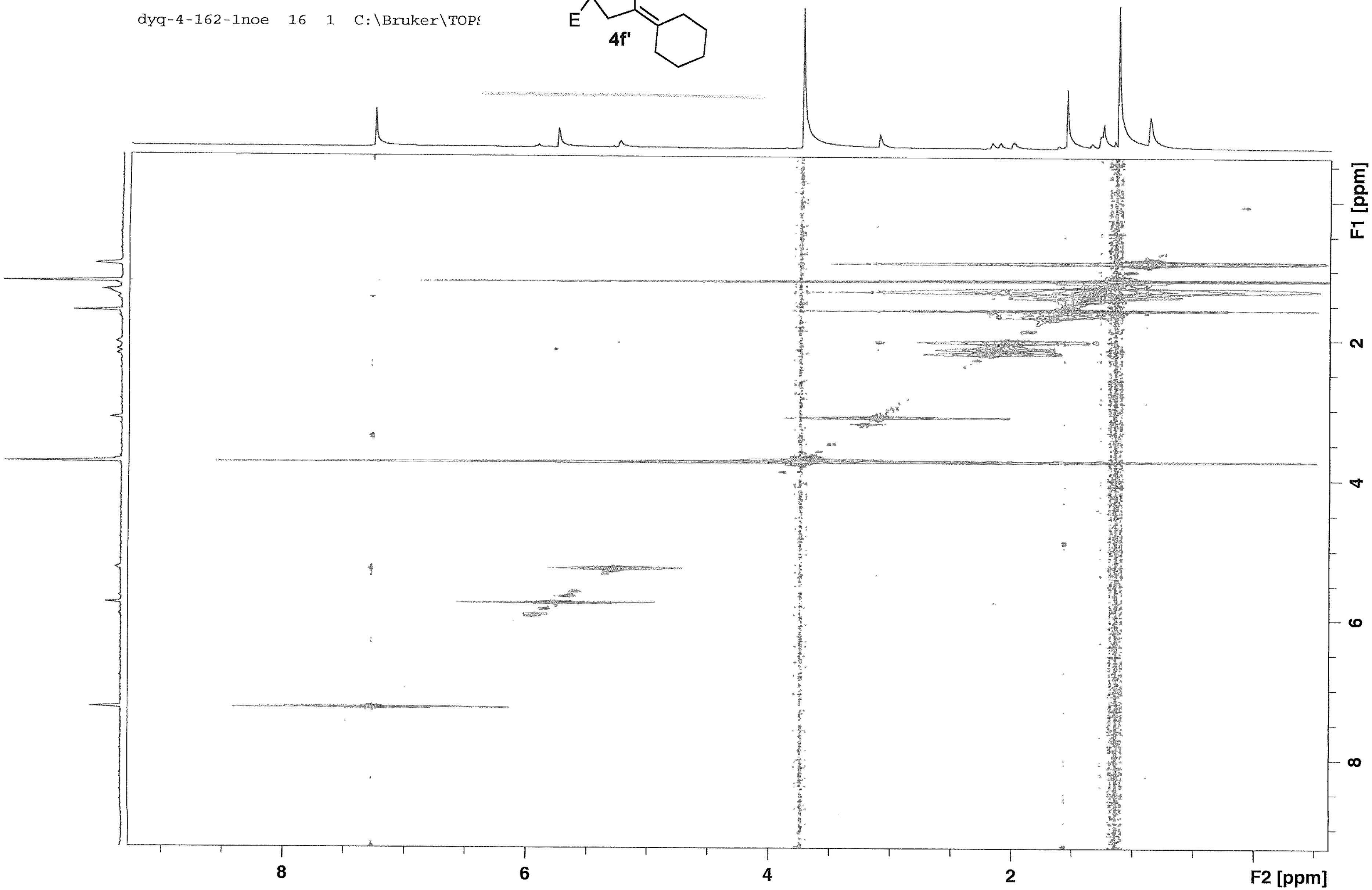

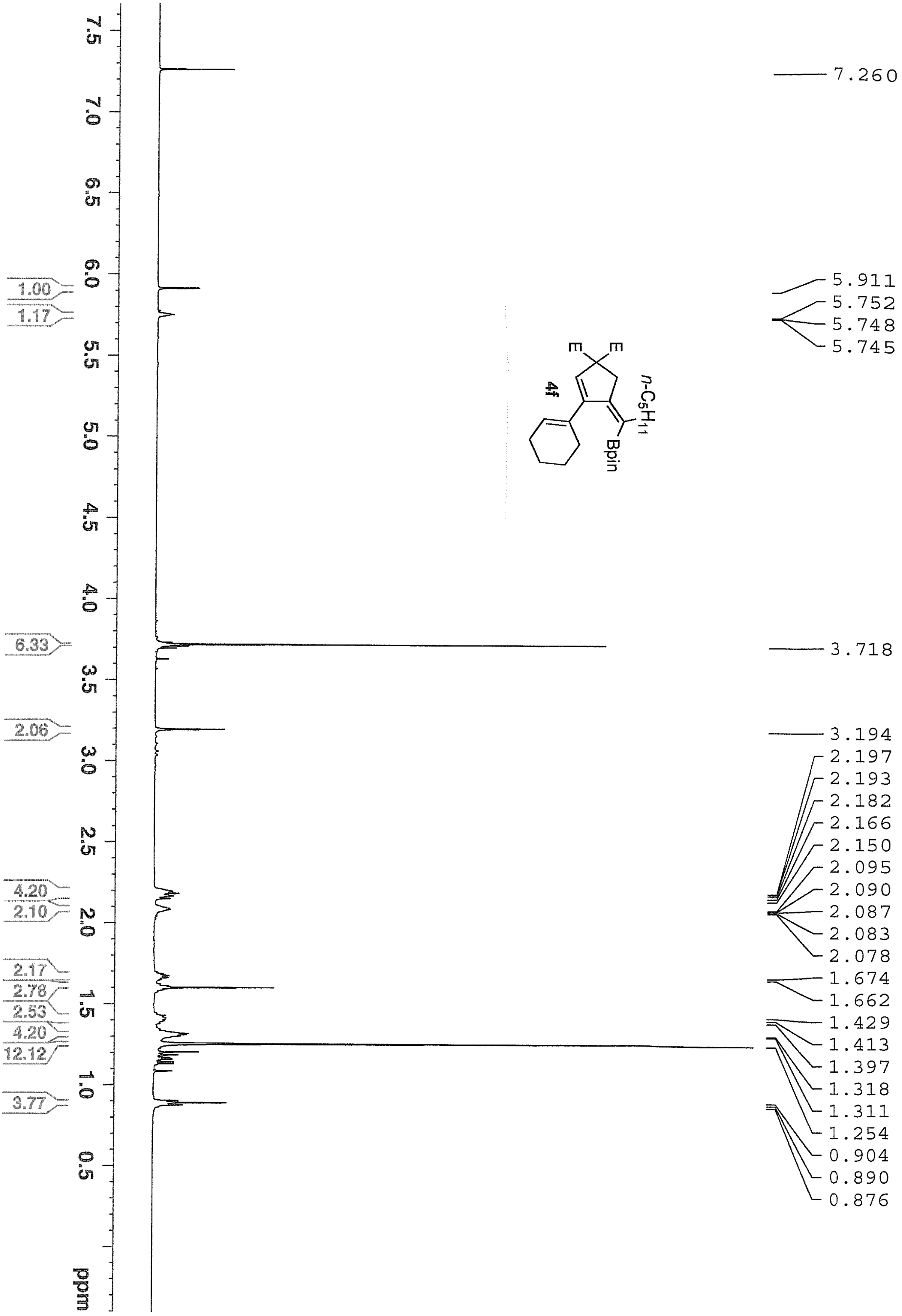
7.260
5.911
5.752
5.748
5.745
3.718
3.194
2.197
2.193
2.182
2.166
2.150
2.095
2.090
2.087
2.083
2.078
1.674
1.662
1.429
1.413
1.397
1.318
1.311
1.254
0.904
0.890
0.876
1.00
1.17
6.33
2.06
4.20
2.10
2.17
2.78
2.53
4.20
12.12
3.77
E
E
n-C5H11
Bpin
4f
ppm

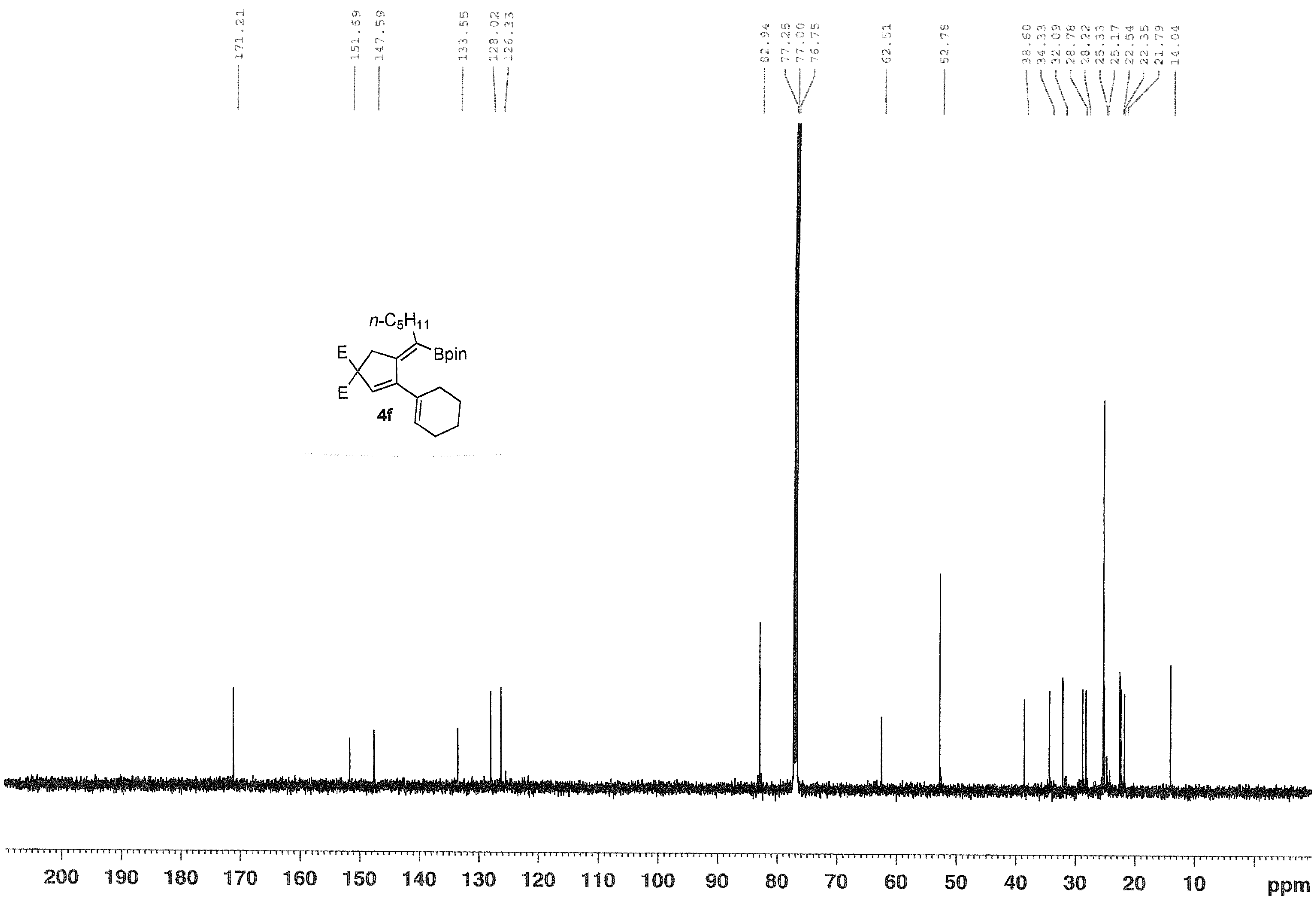
171.21
151.69
147.59
133.55
128.02
126.33
82.94
77.25
77.00
76.75
62.51
52.78
38.60
34.33
32.09
28.78
28.22
25.33
25.17
22.54
22.35
21.79
14.04
n-C5H11
E
E
Bpin
4f
200
190
180
170
160
150
140
130
120
110
100
90
80
70
60
50
40
30
20
10
ppm

Mass Spectrum SmartFormula Report

Analysis Info

Analysis Name	E:\Data2\Youqian\dyq-4-162-2000001.d	Acquisition Date	2012-11-16 14:06:58
Method	Tune_wide_pos.m	Operator	Carin Larsson
Sample Name	dyq-4-162-2	Instrument / Ser#	micrOTOF 125
Comment			

Acquisition Parameter

Source Type	ESI	Ion Polarity	Positive	Set Nebulizer	0.3 Bar
Focus	Not active			Set Dry Heater	180 °C
Scan Begin	50 m/z	Set Capillary	4500 V	Set Dry Gas	4.0 l/min
Scan End	3000 m/z	Set End Plate Offset	-500 V	Set Divert Valve	Source

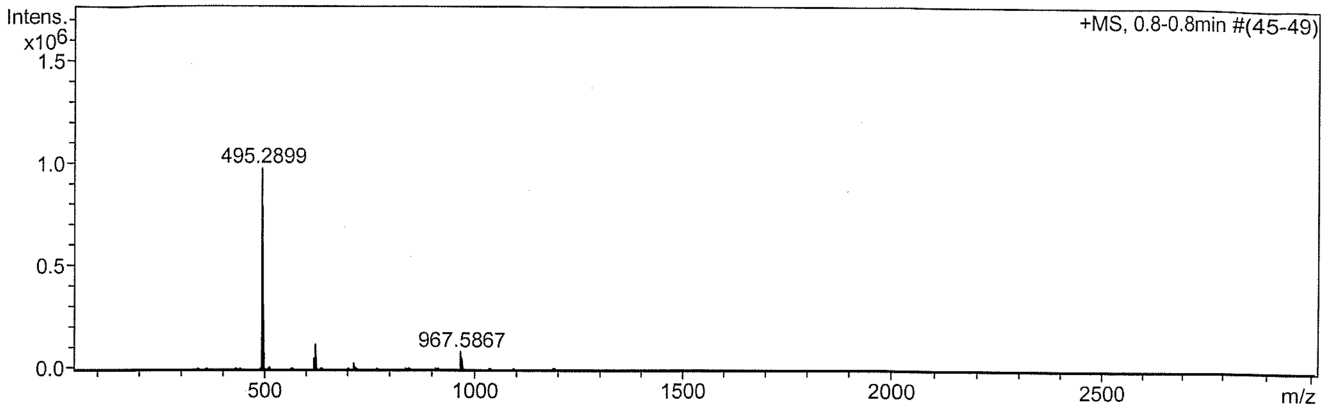

Formula	Meas. m/z	m/z	err [ppm]	Mean err [ppm]
C 27 H 41 B Na O 6	495.2899	495.2893	-1.2	-3.0

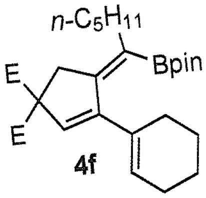

4f

dyq-4-162-2noe 16 1 C:\Bruker\TOPSPIN

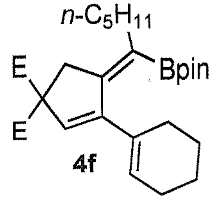

4f

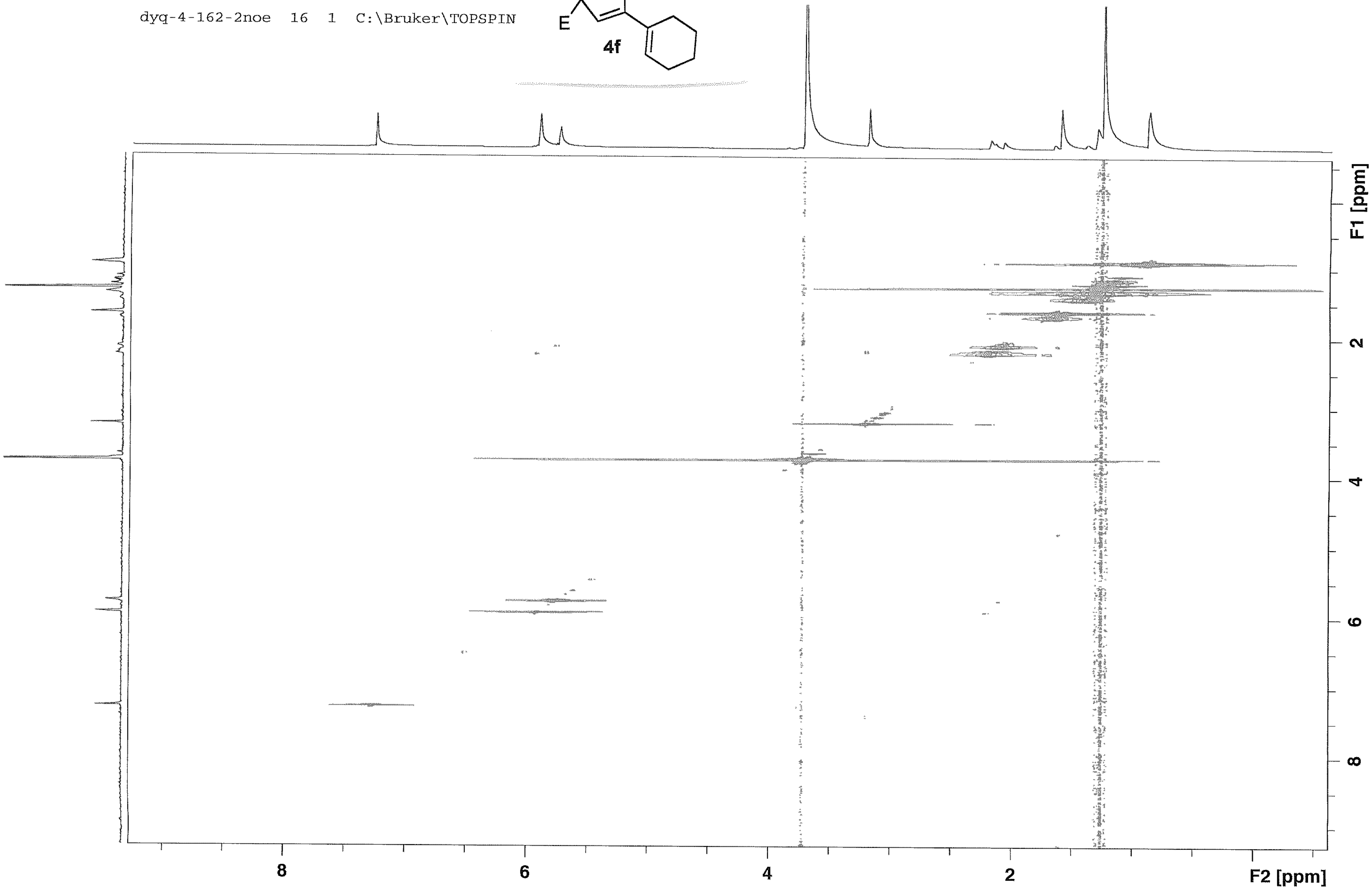

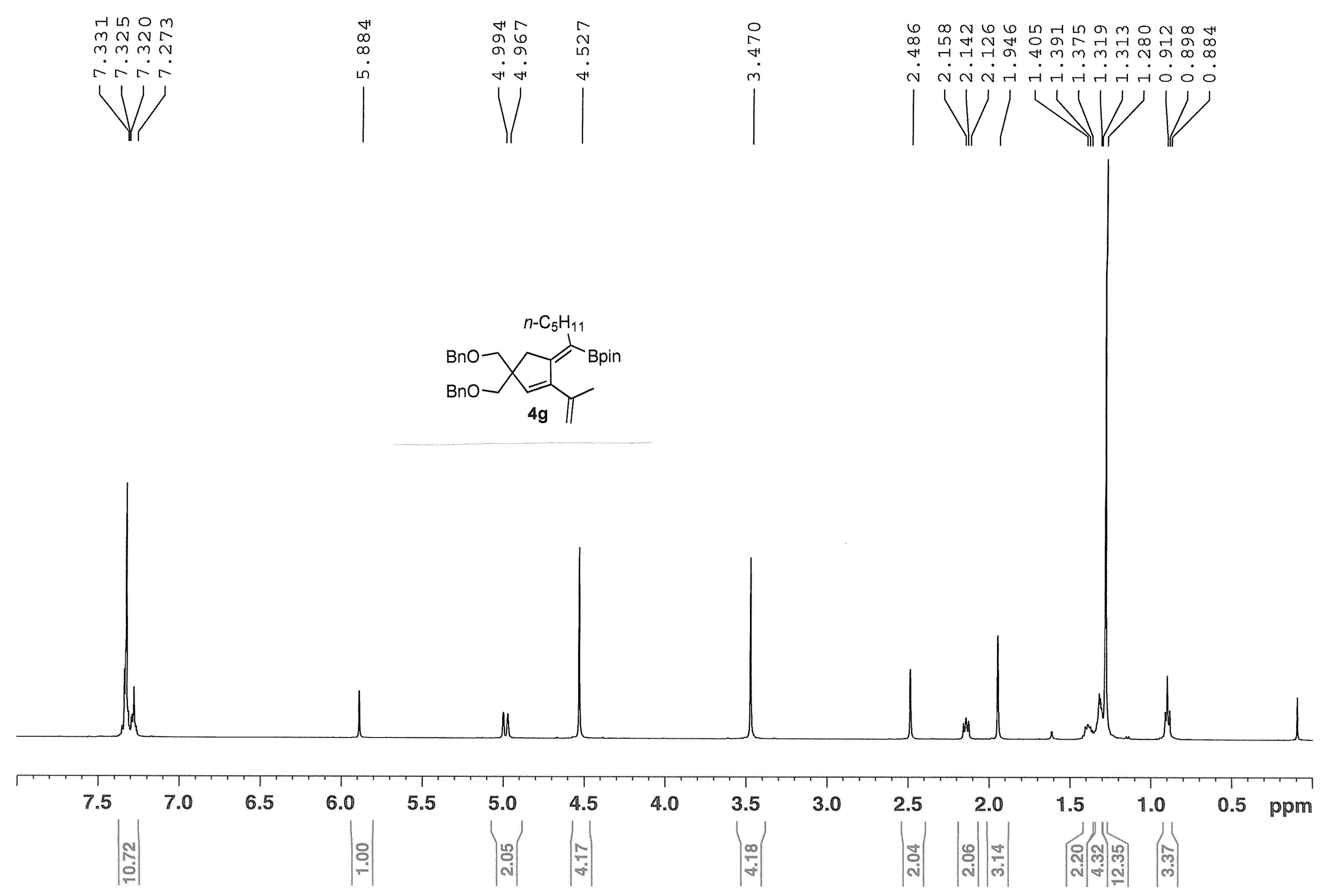

7.331
7.325
7.320
7.273
5.884
4.994
4.967
4.527
3.470
2.486
2.158
2.142
2.126
1.946
1.405
1.391
1.375
1.319
1.313
1.280
0.912
0.898
0.884
n-C5H11
BnO
BnO
Bpin
4g
7.5
7.0
6.5
6.0
5.5
5.0
4.5
4.0
3.5
3.0
2.5
2.0
1.5
1.0
0.5
ppm
10.72
1.00
2.05
4.17
4.18
2.04
2.06
3.14
2.20
4.32
12.35
3.37

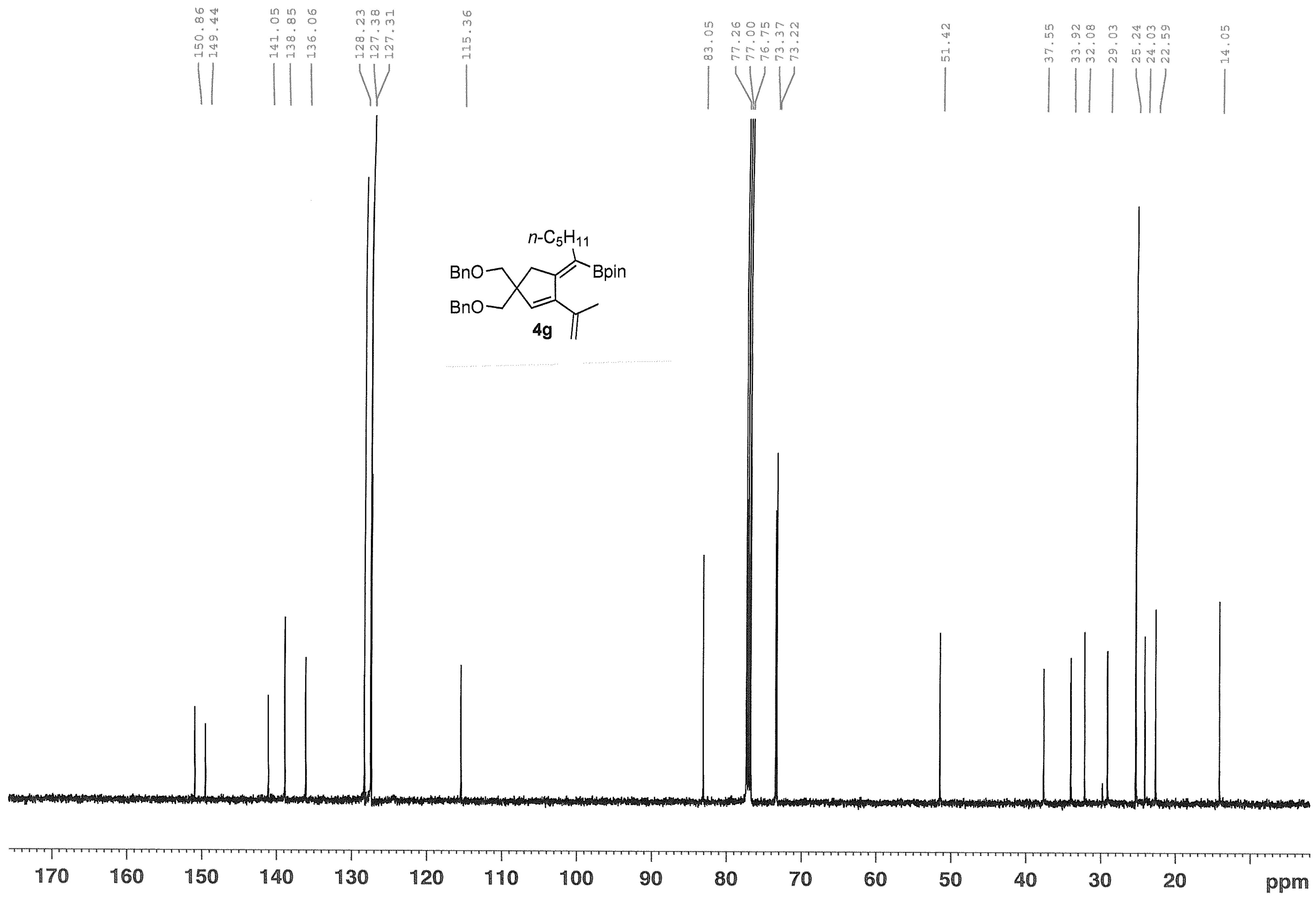
150.86
149.44
141.05
138.85
136.06
128.23
127.38
127.31
115.36
83.05
77.26
77.00
76.75
73.37
73.22
51.42
37.55
33.92
32.08
29.03
25.24
24.03
22.59
14.05
n-C5H11
Bpin
BnO
BnO
4g
170
160
150
140
130
120
110
100
90
80
70
60
50
40
30
20
ppm

Mass Spectrum SmartFormula Report

Analysis Info

Analysis Name	E:\Data2\Youqian\Teb-c-59000001.d	Acquisition Date	2012-12-13 15:42:26
Method	Tune_low_pos.m	Operator	Carin Larsson
Sample Name	Teb-c-59	Instrument / Ser#	micrOTOF 125
Comment			

Acquisition Parameter

Source Type	ESI	Ion Polarity	Positive	Set Nebulizer	0.4 Bar
Focus	Not active			Set Dry Heater	180 °C
Scan Begin	50 m/z	Set Capillary	4000 V	Set Dry Gas	4.0 l/min
Scan End	1000 m/z	Set End Plate Offset	-500 V	Set Divert Valve	Source

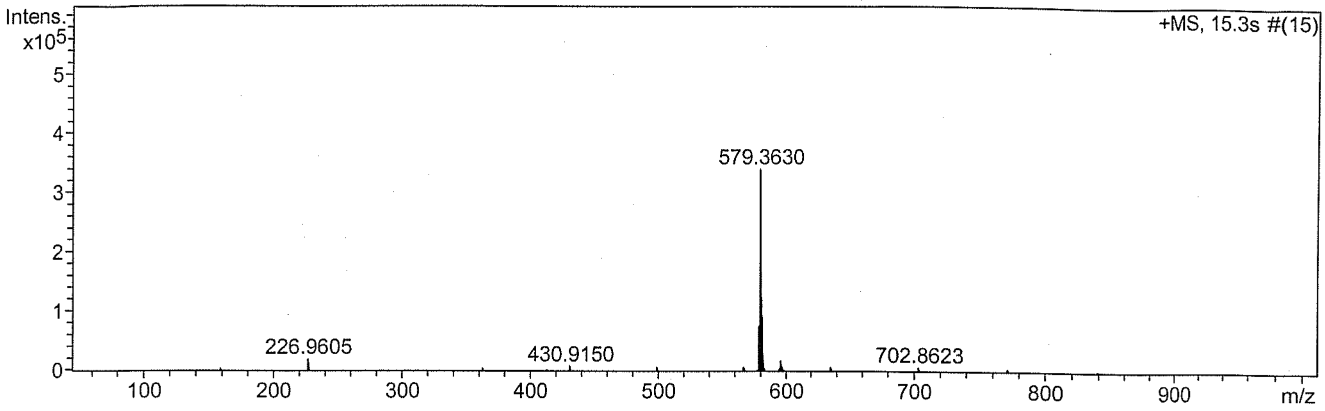

Formula	Meas. m/z	m/z	err [ppm]	Mean err [ppm]
C 36 H 49 B Na O 4	579.3630	579.3622	-1.4	-1.0

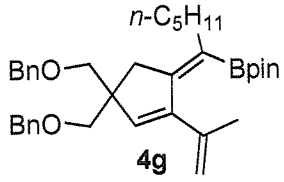

4g

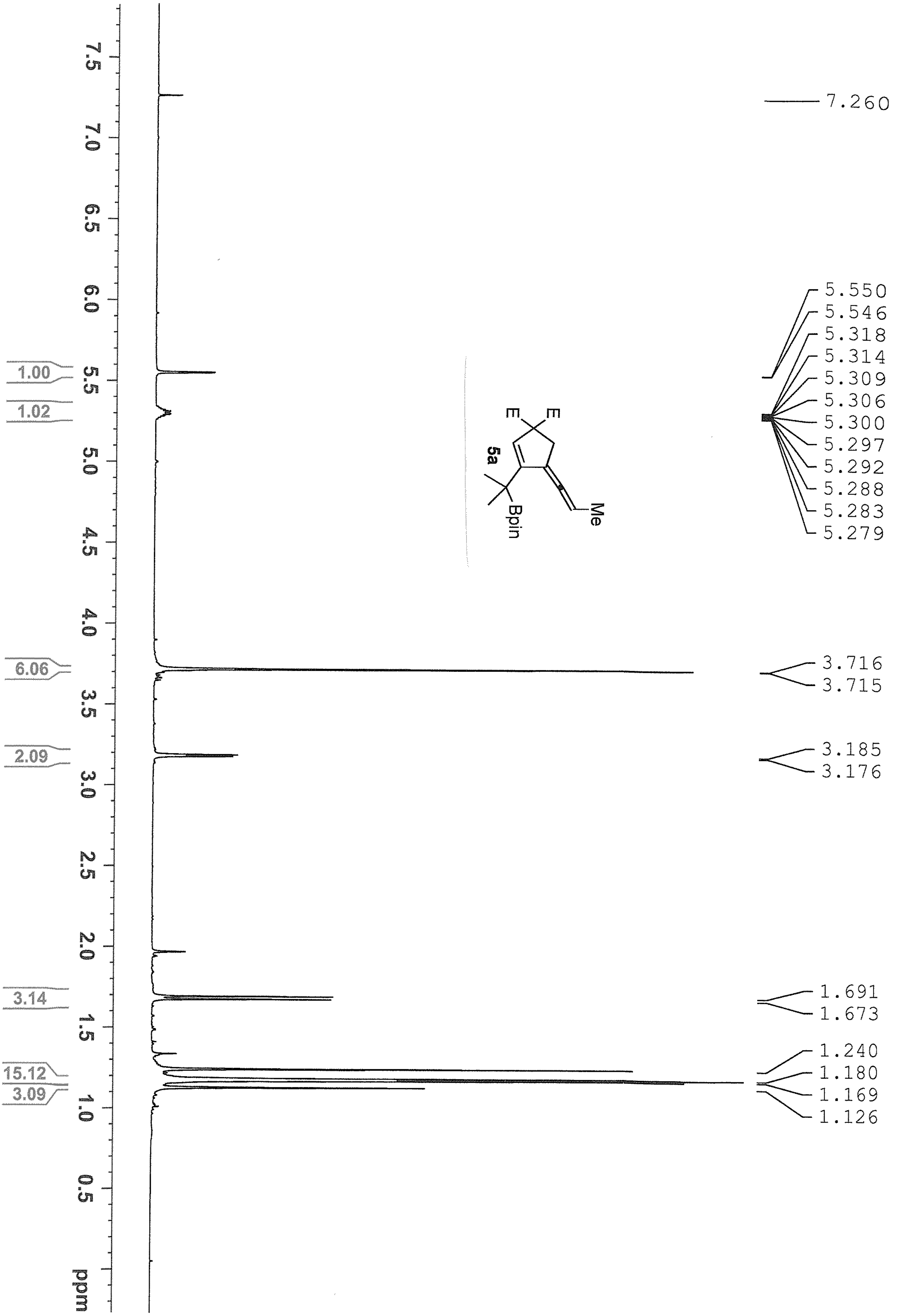
7.260
5.550
5.546
5.318
5.314
5.309
5.306
5.300
5.297
5.292
5.288
5.283
5.279
3.716
3.715
3.185
3.176
1.691
1.673
1.240
1.180
1.169
1.126
1.00
1.02
6.06
2.09
3.14
15.12
3.09
7.5
7.0
6.5
6.0
5.5
5.0
4.5
4.0
3.5
3.0
2.5
2.0
1.5
1.0
0.5
ppm
E
E
5a
Bpin
Me

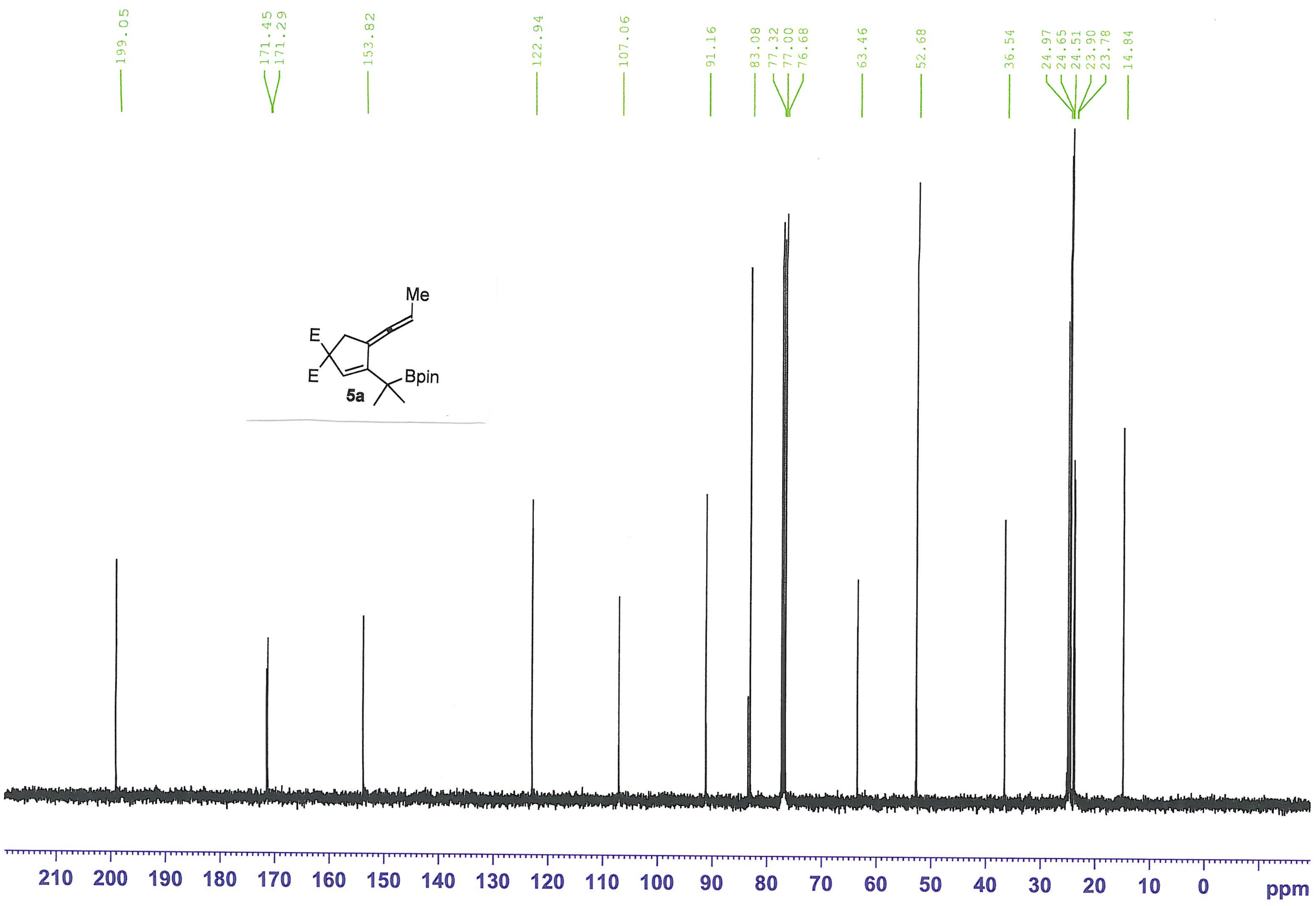
199.05
171.45
171.29
153.82
122.94
107.06
91.16
83.08
77.32
77.00
76.68
63.46
52.68
36.54
24.97
24.65
24.51
23.90
23.78
14.84
Me
E
E
Bpin
5a
210
200
190
180
170
160
150
140
130
120
110
100
90
80
70
60
50
40
30
20
10
0
ppm

Mass Spectrum SmartFormula Report

Analysis Info

Analysis Name	H:\Data2\Youqian\dyq-4-59000001.d	Acquisition Date	2012-08-15 14:35:56
Method	tune_wide_dirk.m	Operator	pia
Sample Name		Instrument / Ser#	micrOTOF 125
Comment			

Acquisition Parameter

Source Type	ESI	Ion Polarity	Positive	Set Nebulizer	0.4 Bar
Focus	Not active			Set Dry Heater	180 °C
Scan Begin	50 m/z	Set Capillary	4500 V	Set Dry Gas	4.0 l/min
Scan End	3000 m/z	Set End Plate Offset	-500 V	Set Divert Valve	Source

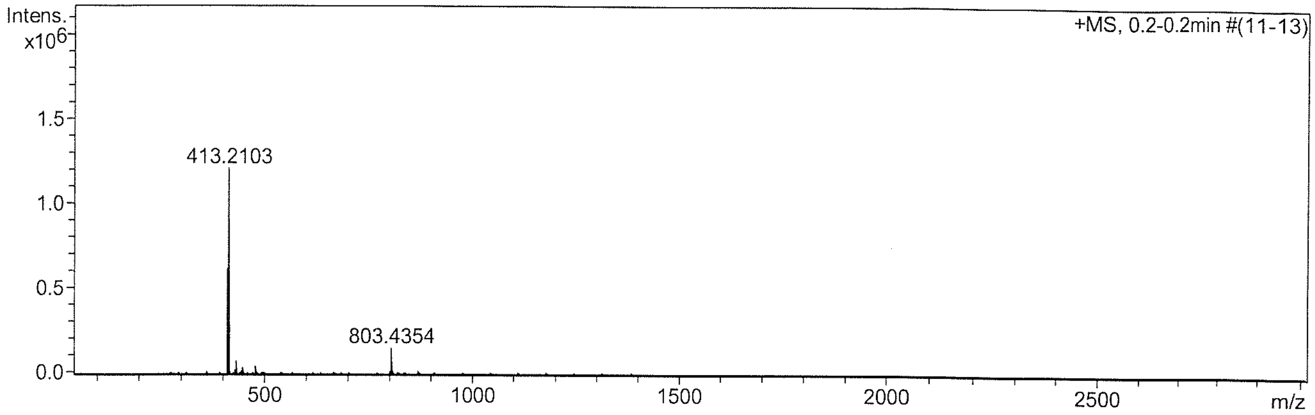

Formula	Meas. m/z	m/z	err [ppm]	Mean err [ppm]
	413.2103			
C 21 H 31 B Na O 6		413.2106	0.7	4.4

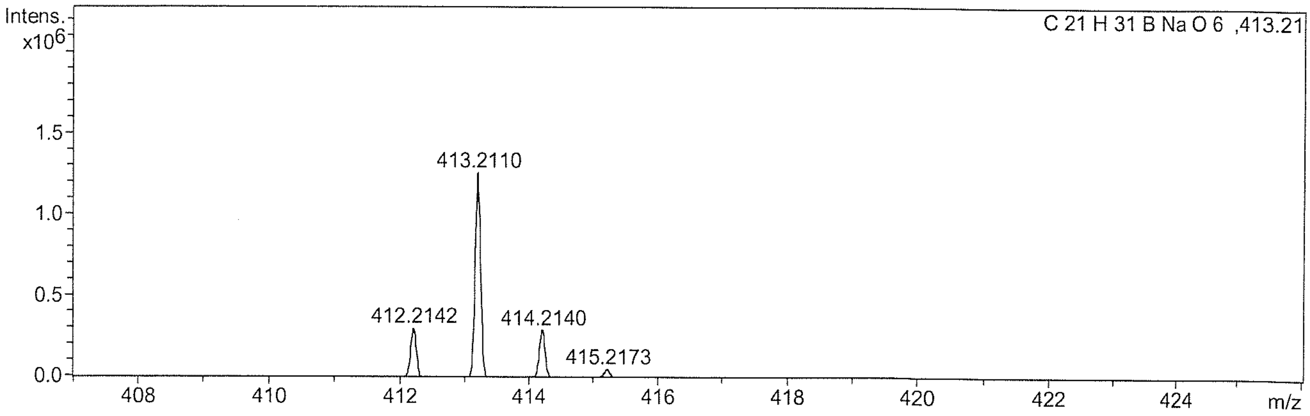

Formula	Meas. m/z	m/z	err [ppm]	Mean err [ppm]

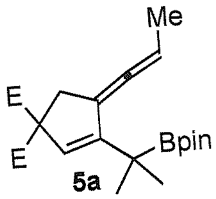

5a

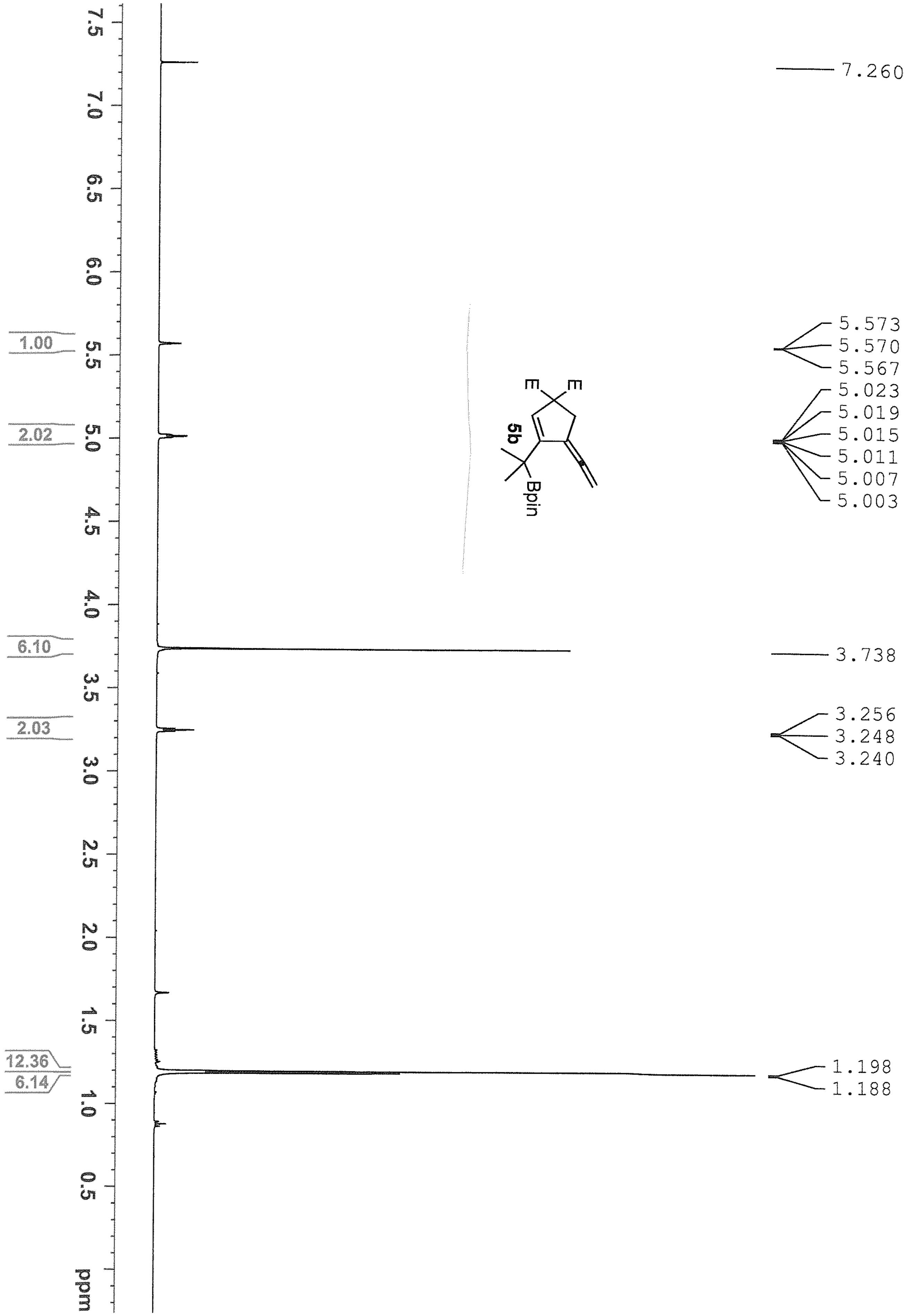

7.260
5.573
5.570
5.567
5.023
5.019
5.015
5.011
5.007
5.003
3.738
3.256
3.248
3.240
1.198
1.188
1.00
2.02
6.10
2.03
12.36
6.14
7.5
7.0
6.5
6.0
5.5
5.0
4.5
4.0
3.5
3.0
2.5
2.0
1.5
1.0
0.5
ppm
5b
E
E
Bpin

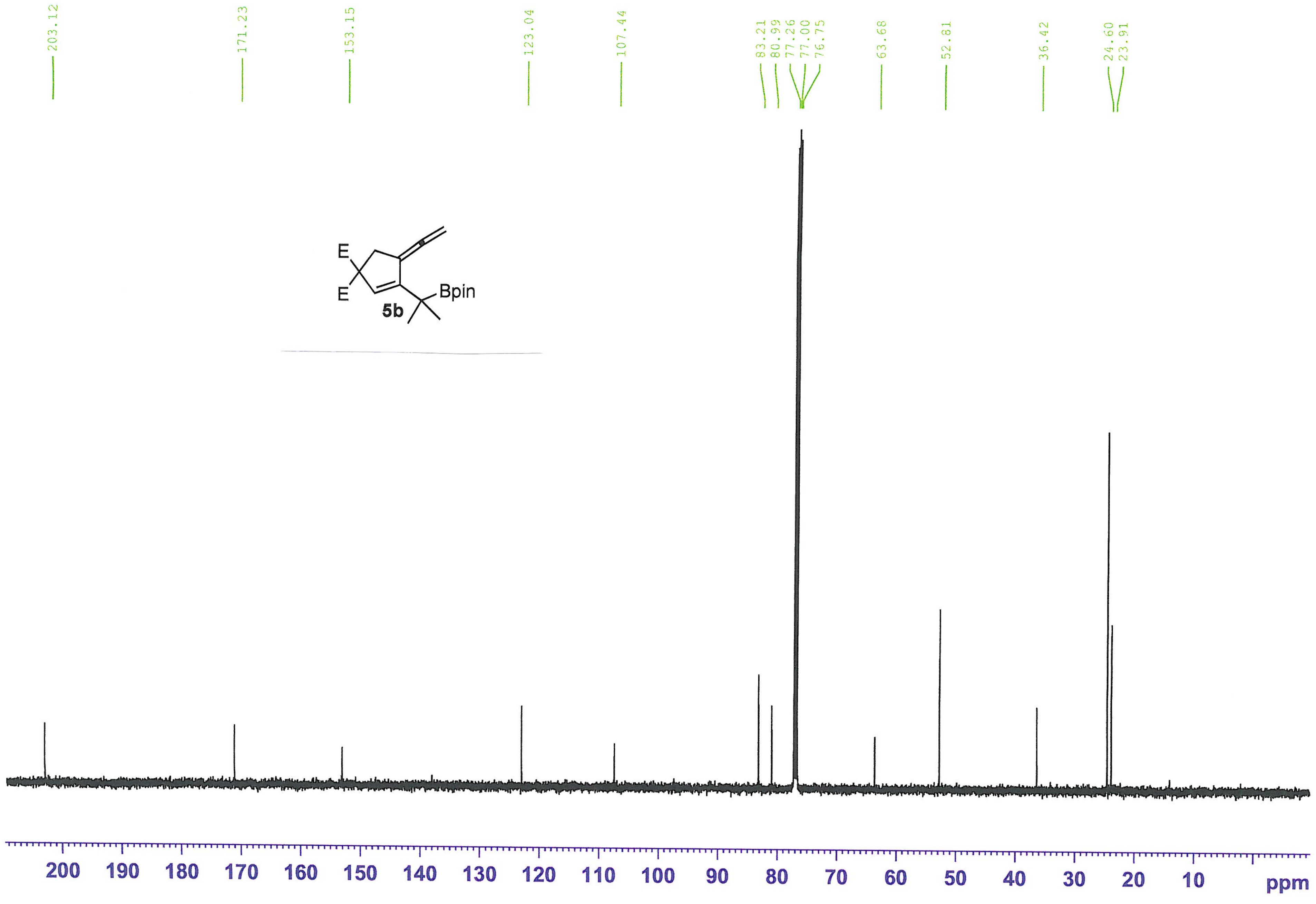

203.12
171.23
153.15
123.04
107.44
83.21
80.99
77.26
77.00
76.75
63.68
52.81
36.42
24.60
23.91
E
E
Bpin
5b
200 190 180 170 160 150 140 130 120 110 100 90 80 70 60 50 40 30 20 10
ppm

Mass Spectrum SmartFormula Report

Analysis Info

Analysis Name	H:\Data2\Youqian\dyq-3-33-2000001.d	Acquisition Date	2011-12-07 11:36:40
Method	tune_wide_dirk.m	Operator	pia
Sample Name	dyq-3-33-2	Instrument / Ser#	micrOTOF 125
Comment			

Acquisition Parameter

Source Type	ESI	Ion Polarity	Positive	Set Nebulizer	0.4 Bar
Focus	Not active			Set Dry Heater	180 °C
Scan Begin	50 m/z	Set Capillary	4500 V	Set Dry Gas	4.0 l/min
Scan End	3000 m/z	Set End Plate Offset	-500 V	Set Divert Valve	Source

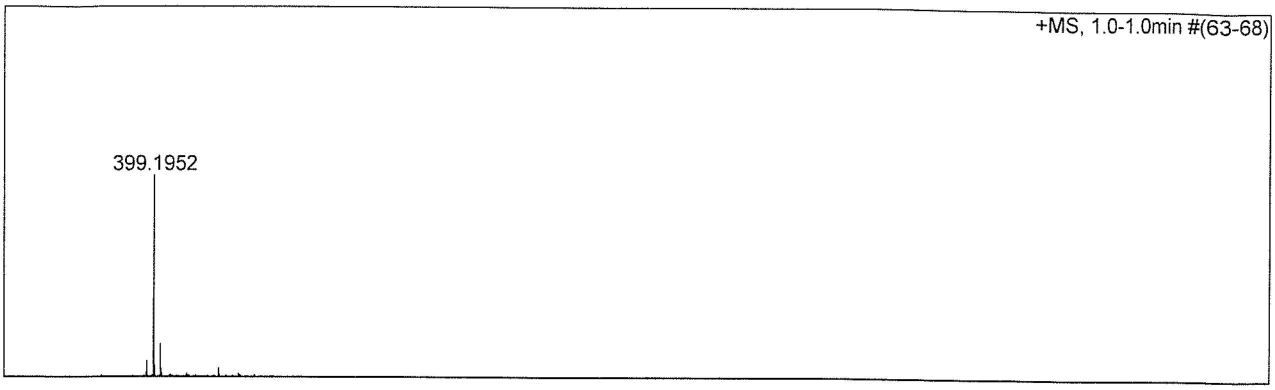

Formula	Meas. m/z	m/z	err [ppm]	Mean err [ppm]
	399.1952			
C 20 H 29 B Na O 6		399.1949	-0.6	4.7

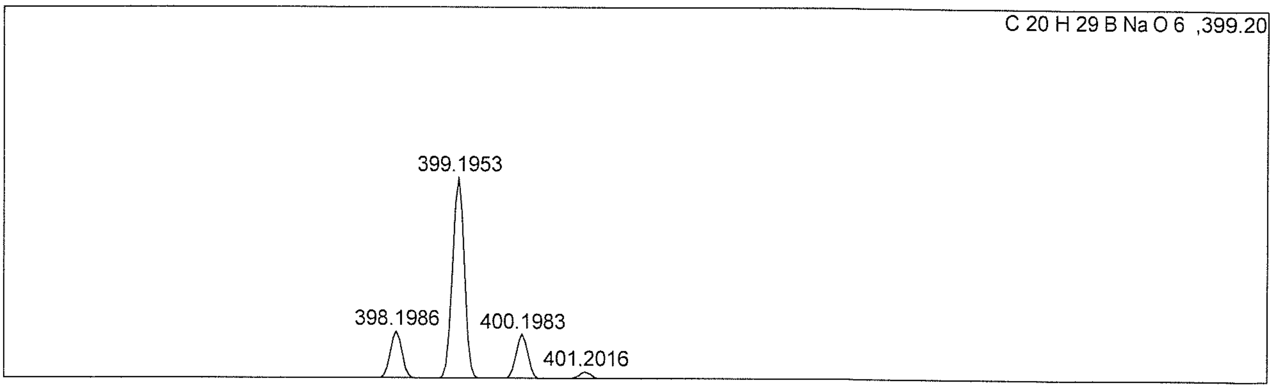

Formula	Meas. m/z	m/z	err [ppm]	Mean err [ppm]

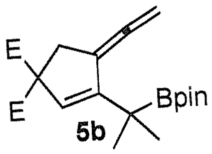

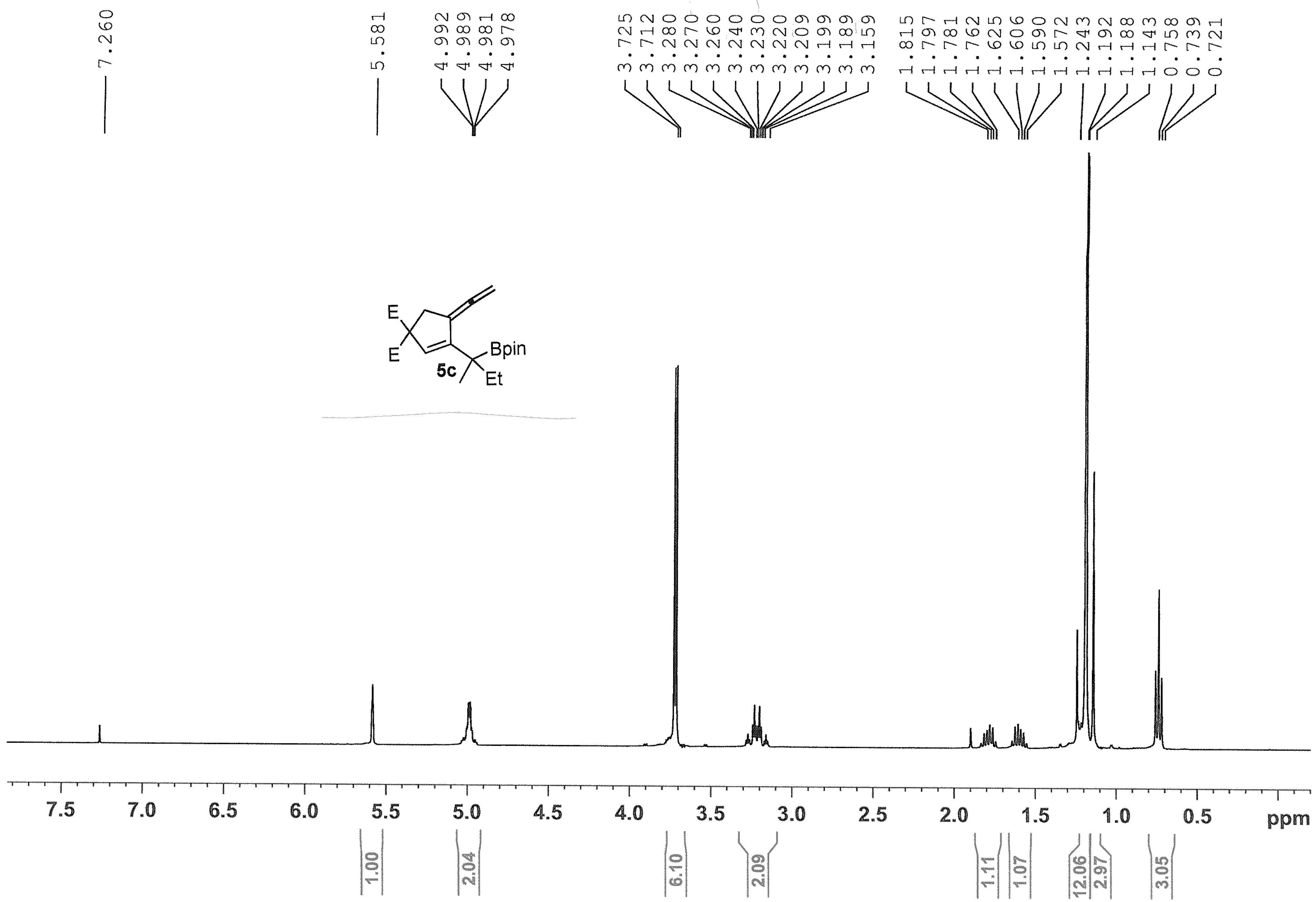
7.260
5.581
4.992
4.989
4.981
4.978
3.725
3.712
3.280
3.270
3.260
3.240
3.230
3.220
3.209
3.199
3.189
3.159
1.815
1.797
1.781
1.762
1.625
1.606
1.590
1.572
1.243
1.192
1.188
1.143
0.758
0.739
0.721
E
E
Bpin
5c
Et
7.5
7.0
6.5
6.0
5.5
5.0
4.5
4.0
3.5
3.0
2.5
2.0
1.5
1.0
0.5
ppm
1.00
2.04
6.10
2.09
1.11
1.07
12.06
2.97
3.05

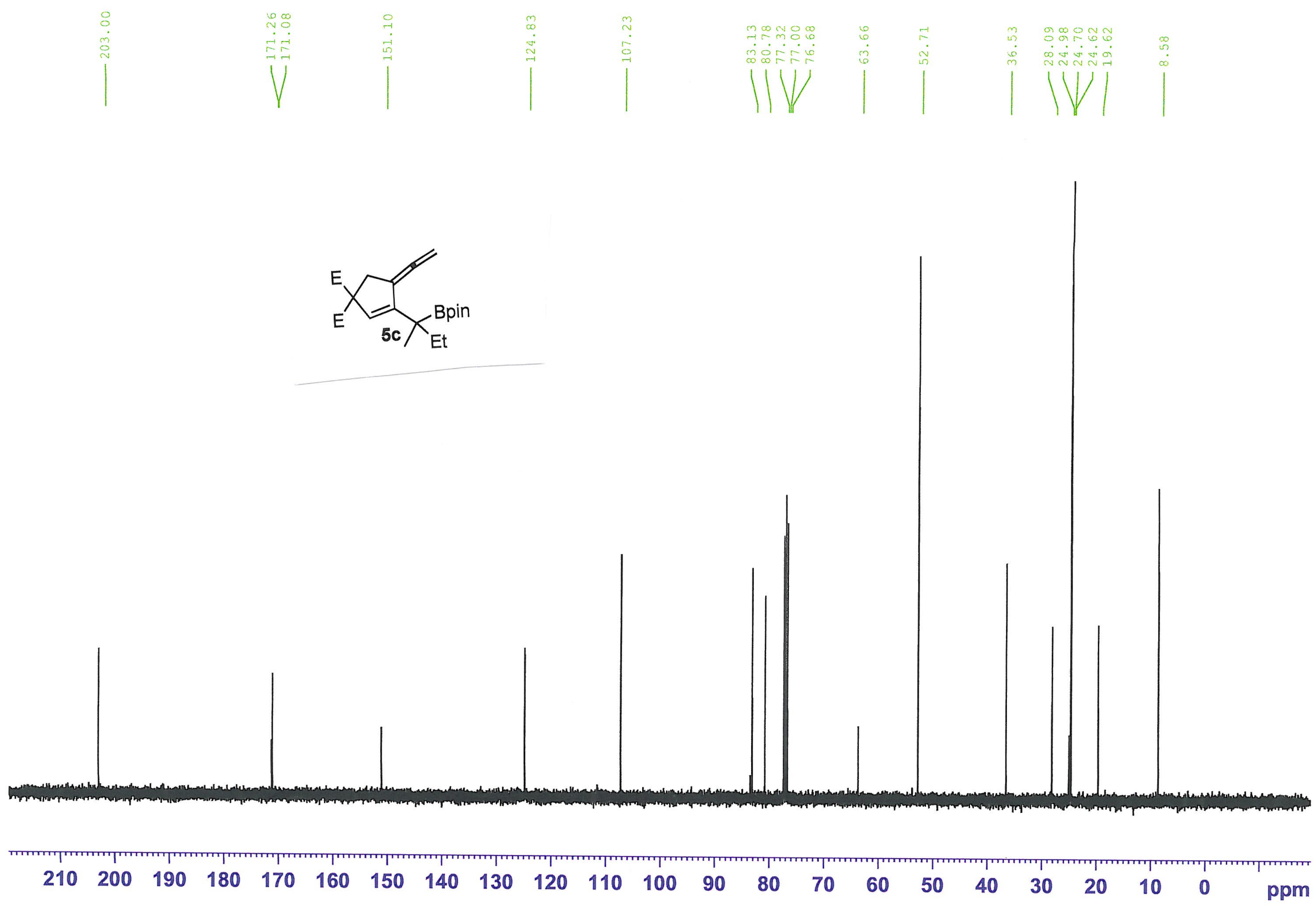
203.00
171.26
171.08
151.10
124.83
107.23
83.13
80.78
77.32
77.00
76.68
63.66
52.71
36.53
28.09
24.98
24.70
24.62
19.62
8.58
E
E
Bpin
5c
Et
210
200
190
180
170
160
150
140
130
120
110
100
90
80
70
60
50
40
30
20
10
0
ppm

Mass Spectrum SmartFormula Report

Analysis Info

Analysis Name	H:\Data2\Youqian\dyq-3-179000001.d	Acquisition Date	2012-04-30 10:37:06
Method	tune_wide_dirk.m	Operator	pia
Sample Name	dyq-3-179	Instrument / Ser#	micrOTOF 125
Comment			

Acquisition Parameter

Source Type	ESI	Ion Polarity	Positive	Set Nebulizer	0.4 Bar
Focus	Not active			Set Dry Heater	180 °C
Scan Begin	50 m/z	Set Capillary	4500 V	Set Dry Gas	4.0 l/min
Scan End	3000 m/z	Set End Plate Offset	-500 V	Set Divert Valve	Source

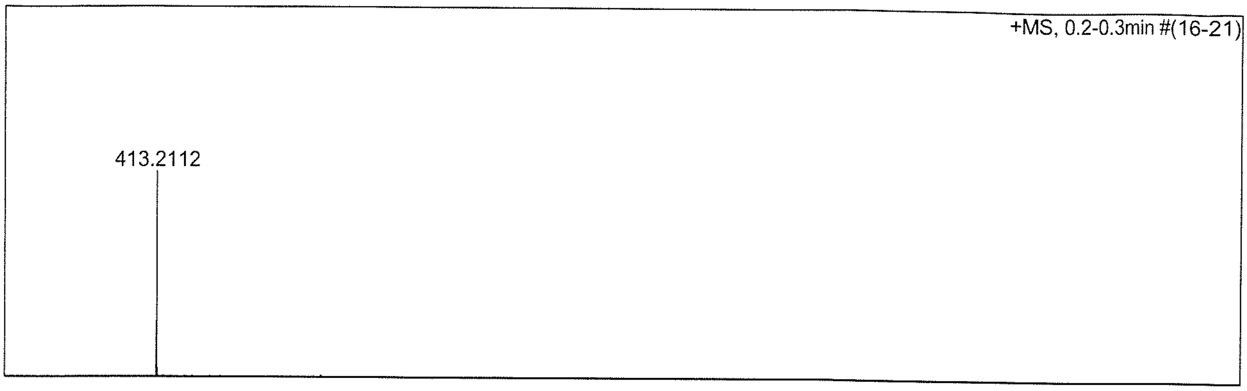

Formula	Meas. m/z	m/z	err [ppm]	Mean err [ppm]
	413.2112			
C 21 H 31 B Na O 6		413.2106	-1.4	1.4

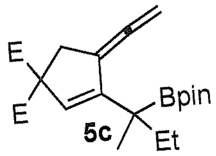

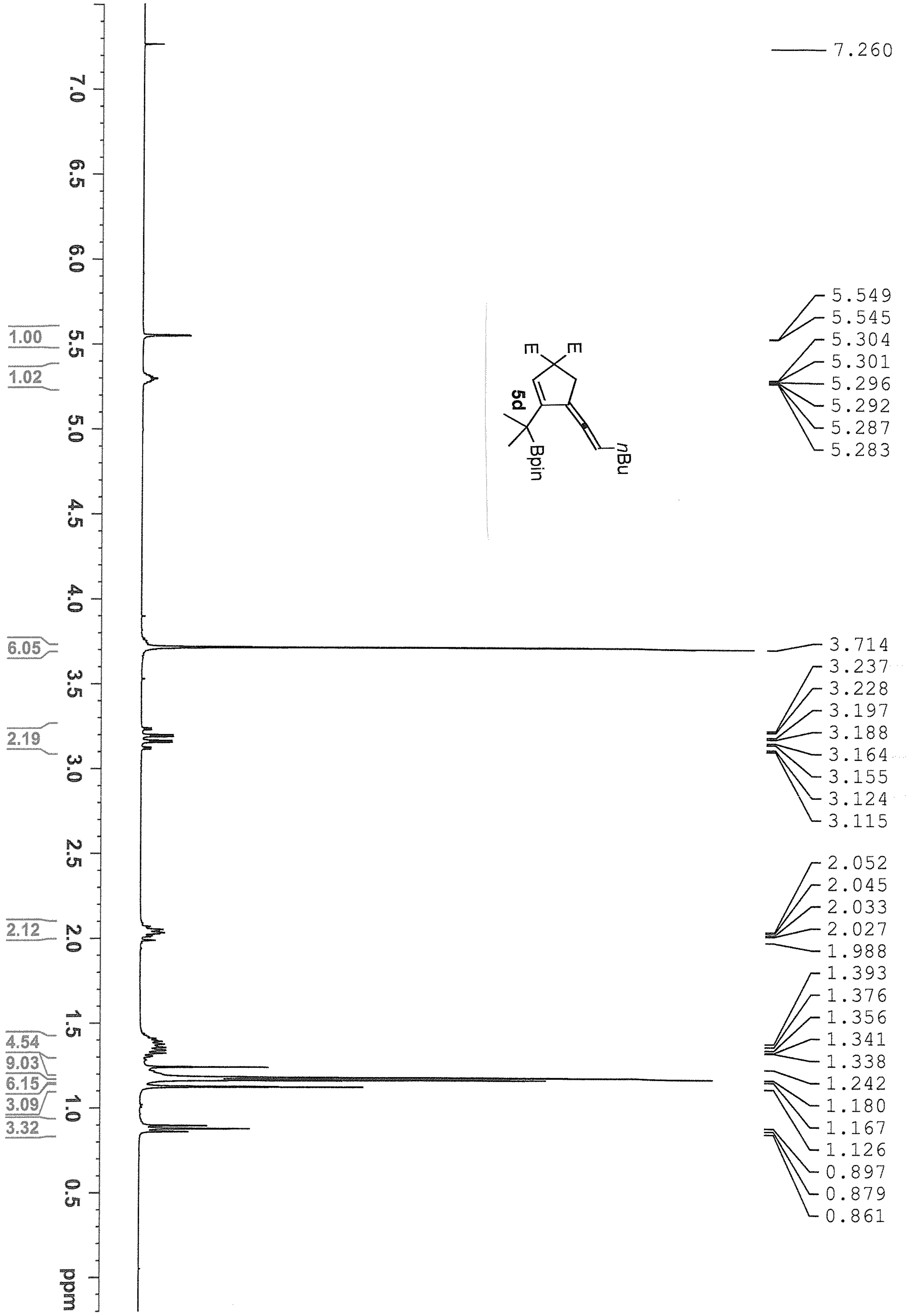

7.260
5.549
5.545
5.304
5.301
5.296
5.292
5.287
5.283
3.714
3.237
3.228
3.197
3.188
3.164
3.155
3.124
3.115
2.052
2.045
2.033
2.027
1.988
1.393
1.376
1.356
1.341
1.338
1.242
1.180
1.167
1.126
0.897
0.879
0.861
1.00
1.02
6.05
2.19
2.12
4.54
9.03
6.15
3.09
3.32
7.0
6.5
6.0
5.5
5.0
4.5
4.0
3.5
3.0
2.5
2.0
1.5
1.0
0.5
ppm
E
E
5d
Bpin
nBu

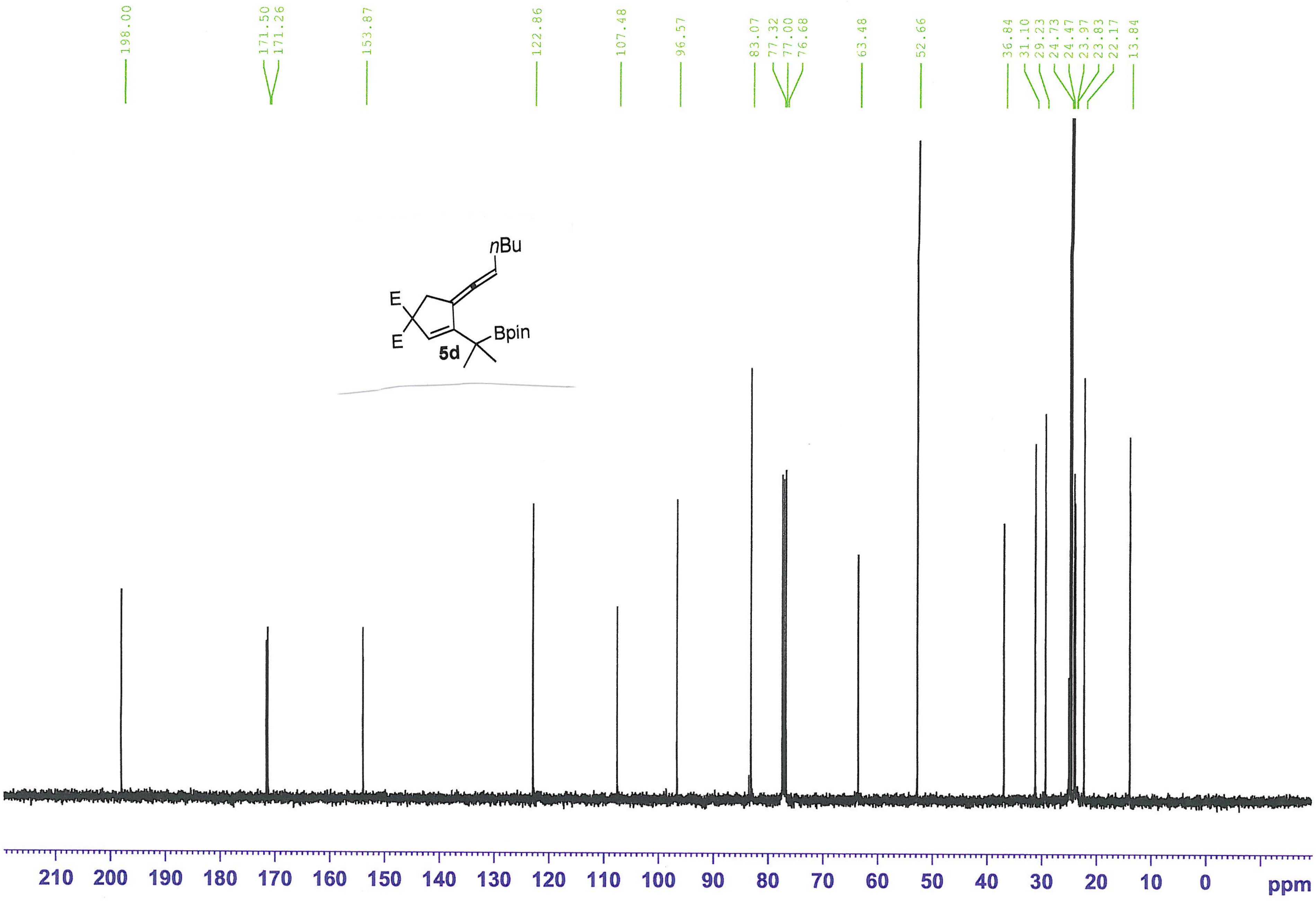
198.00
171.50
171.26
153.87
122.86
107.48
96.57
83.07
77.32
77.00
76.68
63.48
52.66
36.84
31.10
29.23
24.73
24.47
23.97
23.83
22.17
13.84
nBu
E
E
Bpin
5d
210
200
190
180
170
160
150
140
130
120
110
100
90
80
70
60
50
40
30
20
10
0
ppm

Mass Spectrum SmartFormula Report

Analysis Info

Analysis Name	H:\Data2\Youqian\dyq-3-167000002.d	Acquisition Date	2012-04-26 18:08:48
Method	tune_wide_dirk.m	Operator	pia
Sample Name	dyq-3-167	Instrument / Ser#	micrOTOF 125
Comment			

Acquisition Parameter

Source Type	ESI	Ion Polarity	Positive	Set Nebulizer	0.4 Bar
Focus	Not active			Set Dry Heater	180 °C
Scan Begin	50 m/z	Set Capillary	4500 V	Set Dry Gas	4.0 l/min
Scan End	3000 m/z	Set End Plate Offset	-500 V	Set Divert Valve	Source

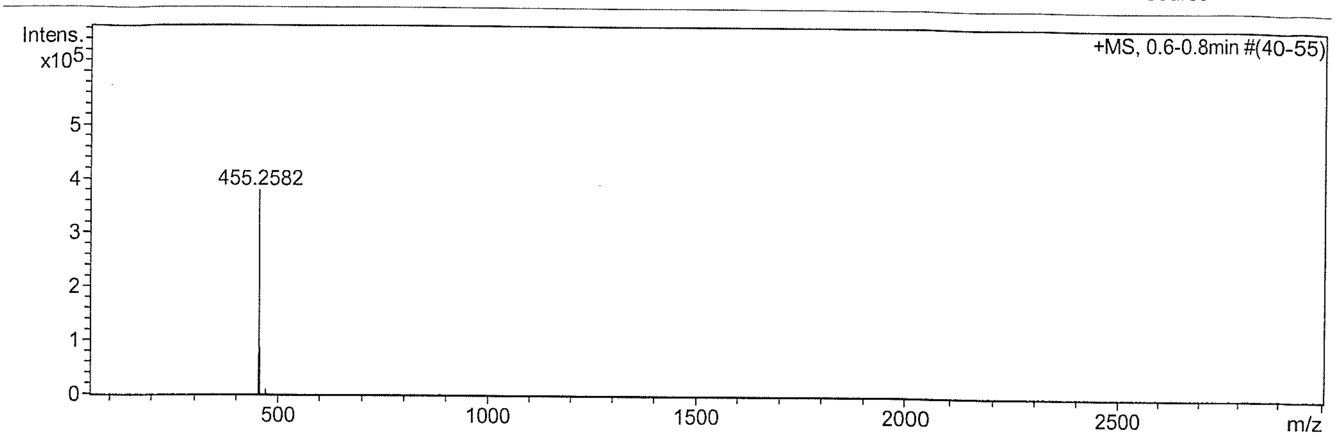

Formula	Meas. m/z	m/z	err [ppm]	Mean err [ppm]
	455.2582			
C 24 H 37 B Na O 6		455.2575	-1.5	-0.2
	456.2605			
C 24 H 37 B Na O 6		455.2575	1.5	-0.2

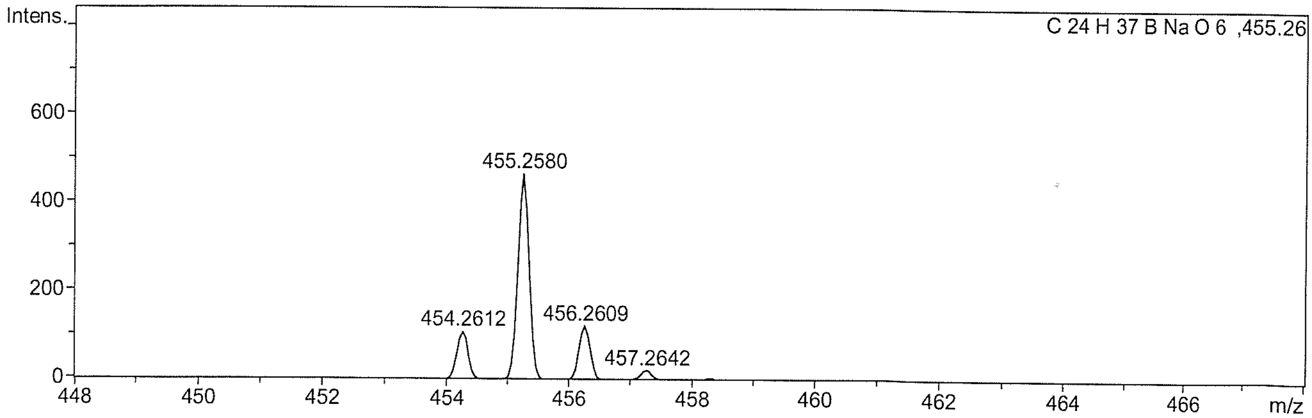

Formula	Meas. m/z	m/z	err [ppm]	Mean err [ppm]

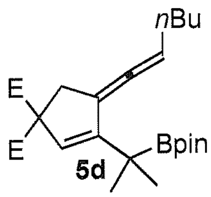

5d

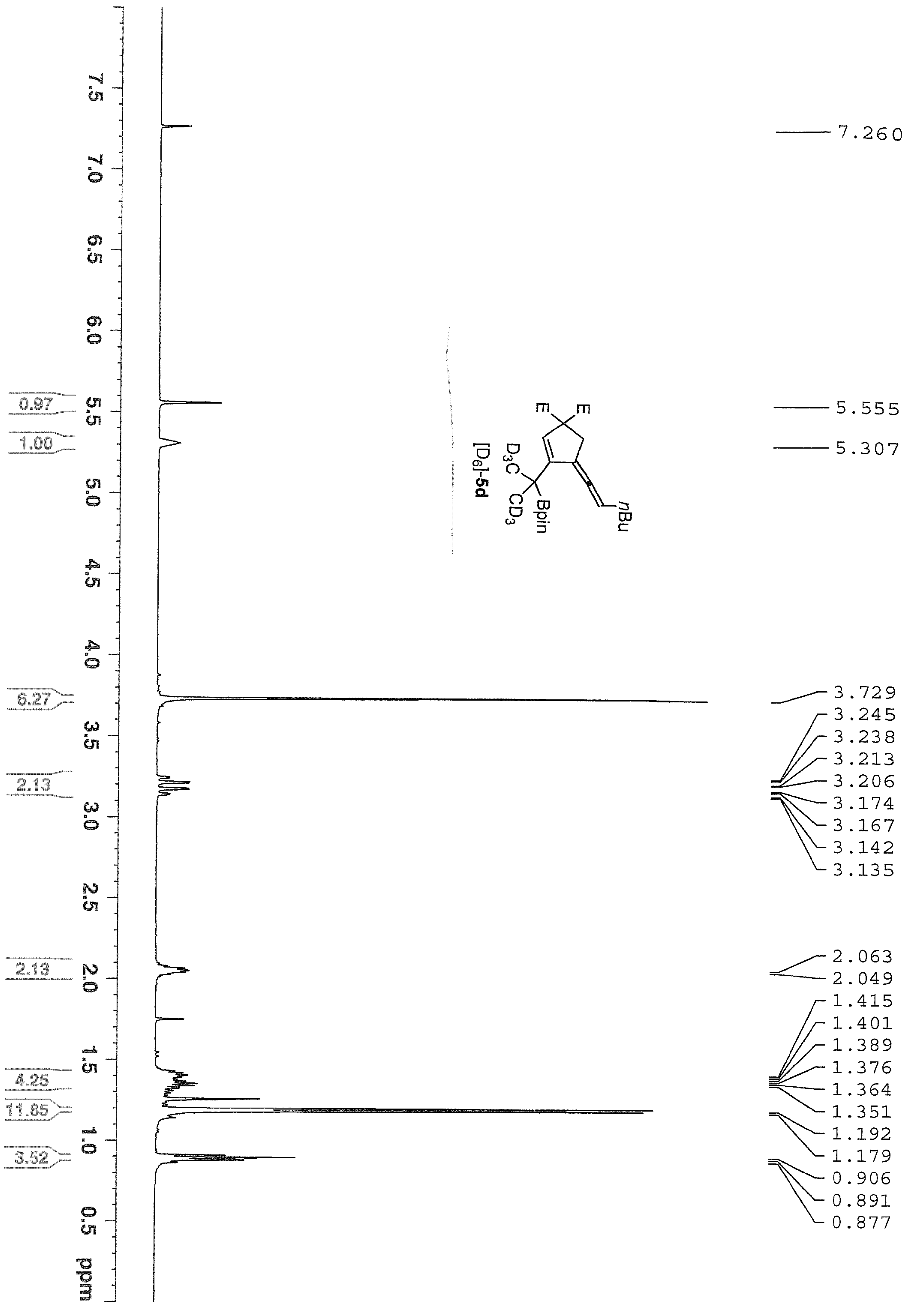

[D6]-5d
7.260
5.555
5.307
3.729
3.245
3.238
3.213
3.206
3.174
3.167
3.142
3.135
2.063
2.049
1.415
1.401
1.389
1.376
1.364
1.351
1.192
1.179
0.906
0.891
0.877
0.97
1.00
6.27
2.13
2.13
4.25
11.85
3.52
ppm

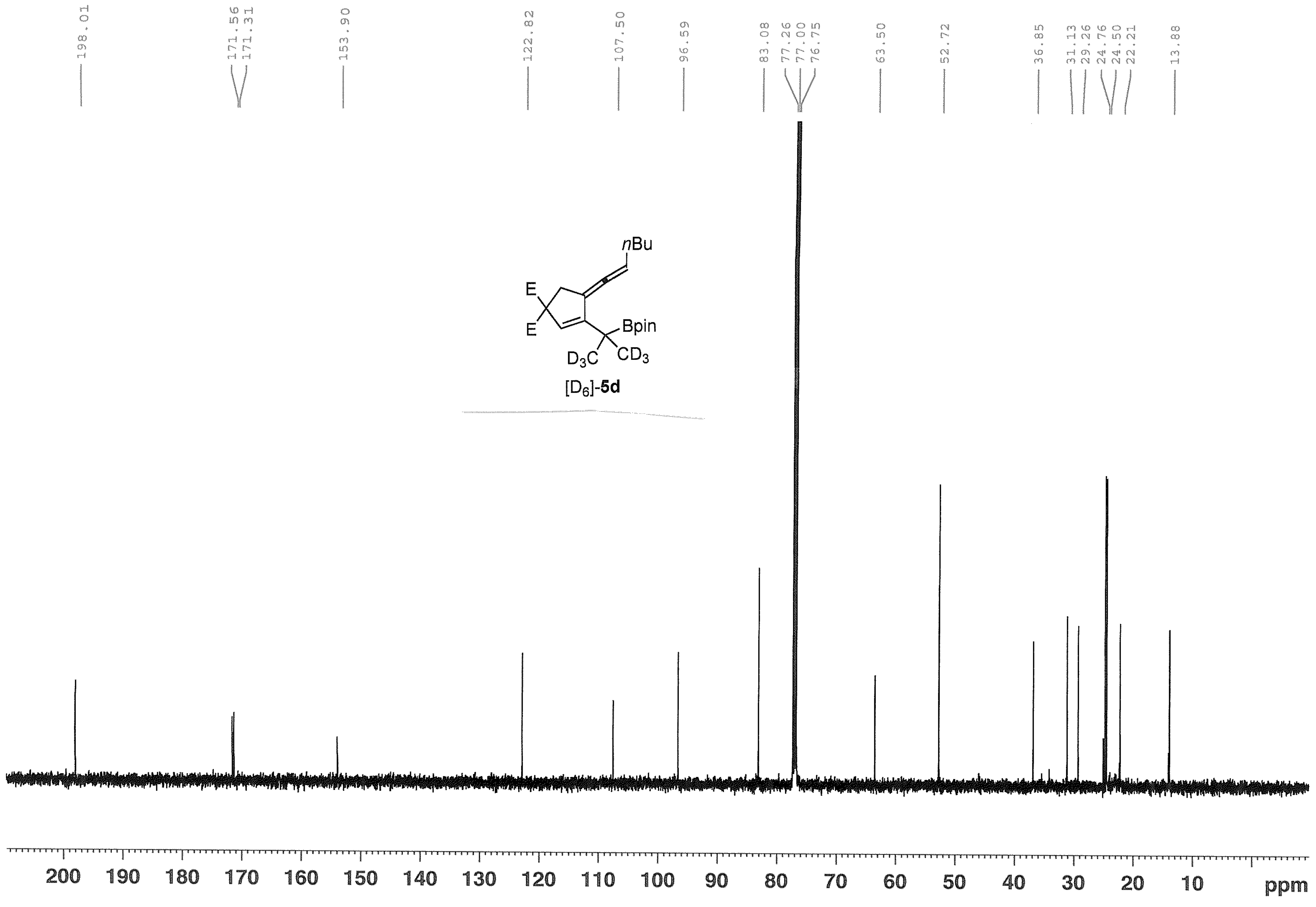
198.01
171.56
171.31
153.90
122.82
107.50
96.59
83.08
77.26
77.00
76.75
63.50
52.72
36.85
31.13
29.26
24.76
24.50
22.21
13.88
nBu
E
E
Bpin
D_3C
CD_3
[D_6]-**5d**
200
190
180
170
160
150
140
130
120
110
100
90
80
70
60
50
40
30
20
10
ppm

Mass Spectrum SmartFormula Report

Analysis Info

Analysis Name	E:\Data2\Youqian\dyq-4-185000001.d	Acquisition Date	2012-12-07 18:34:02
Method	Tune_low_pos.m	Operator	Carin Larsson
Sample Name	dyq-4-185	Instrument / Ser#	micrOTOF 125
Comment			

Acquisition Parameter

Source Type	ESI	Ion Polarity	Positive	Set Nebulizer	0.4 Bar
Focus	Not active			Set Dry Heater	180 °C
Scan Begin	50 m/z	Set Capillary	4000 V	Set Dry Gas	4.0 l/min
Scan End	1000 m/z	Set End Plate Offset	-500 V	Set Divert Valve	Source

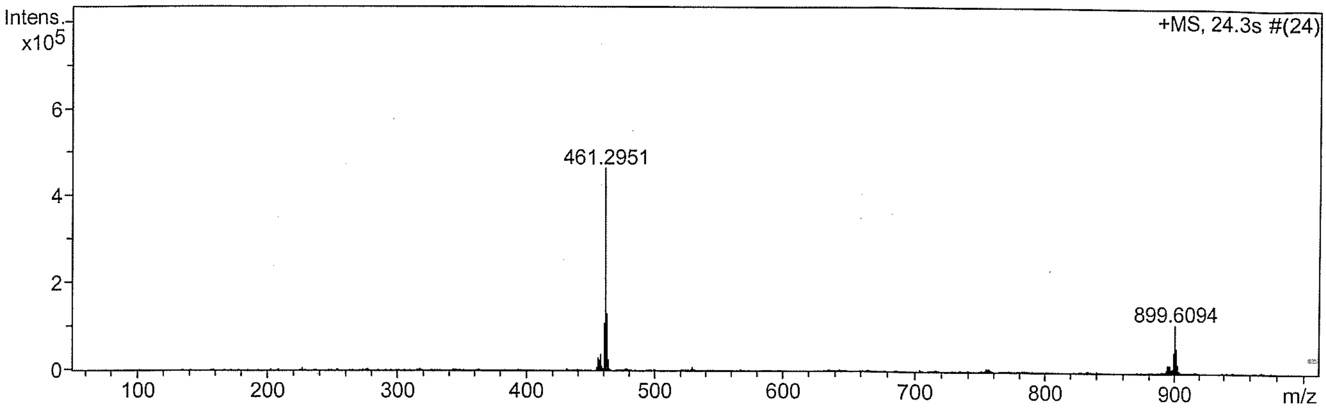

Formula	Meas. m/z	m/z	err [ppm]	Mean err [ppm]
C 24 H 31 B D 6 Na O 6	461.2951	461.2956	0.2	1.7

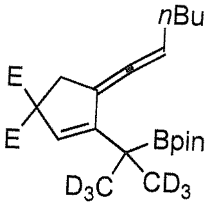

[D6]-**5d**

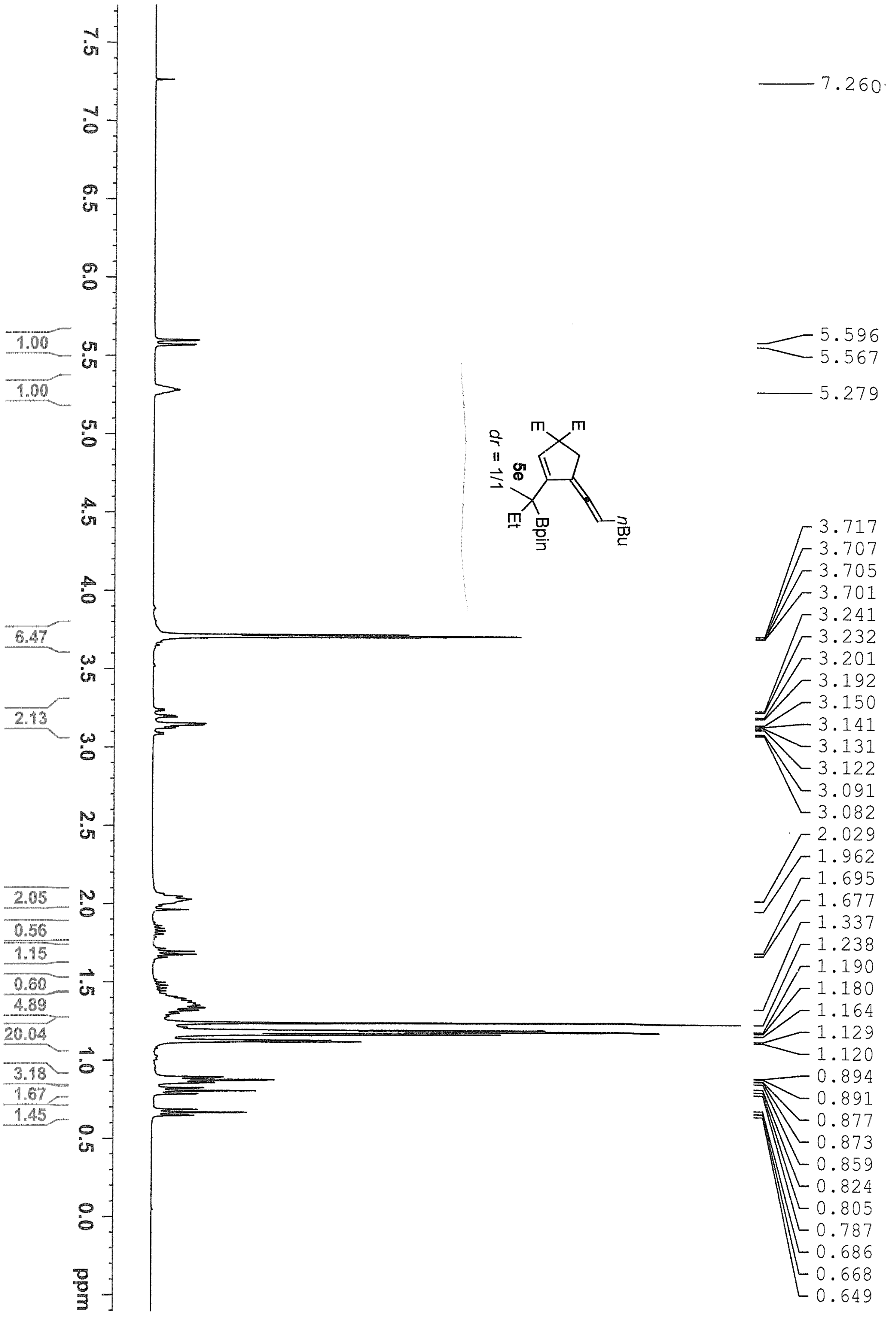
E
E
nBu
5e
Bpin
Et
dr = 1/1
7.260
5.596
5.567
5.279
3.717
3.707
3.705
3.701
3.241
3.232
3.201
3.192
3.150
3.141
3.131
3.122
3.091
3.082
2.029
1.962
1.695
1.677
1.337
1.238
1.190
1.180
1.164
1.129
1.120
0.894
0.891
0.877
0.873
0.859
0.824
0.805
0.787
0.686
0.668
0.649
1.00
1.00
6.47
2.13
2.05
0.56
1.15
0.60
4.89
20.04
3.18
1.67
1.45
7.5
7.0
6.5
6.0
5.5
5.0
4.5
4.0
3.5
3.0
2.5
2.0
1.5
1.0
0.5
0.0
ppm

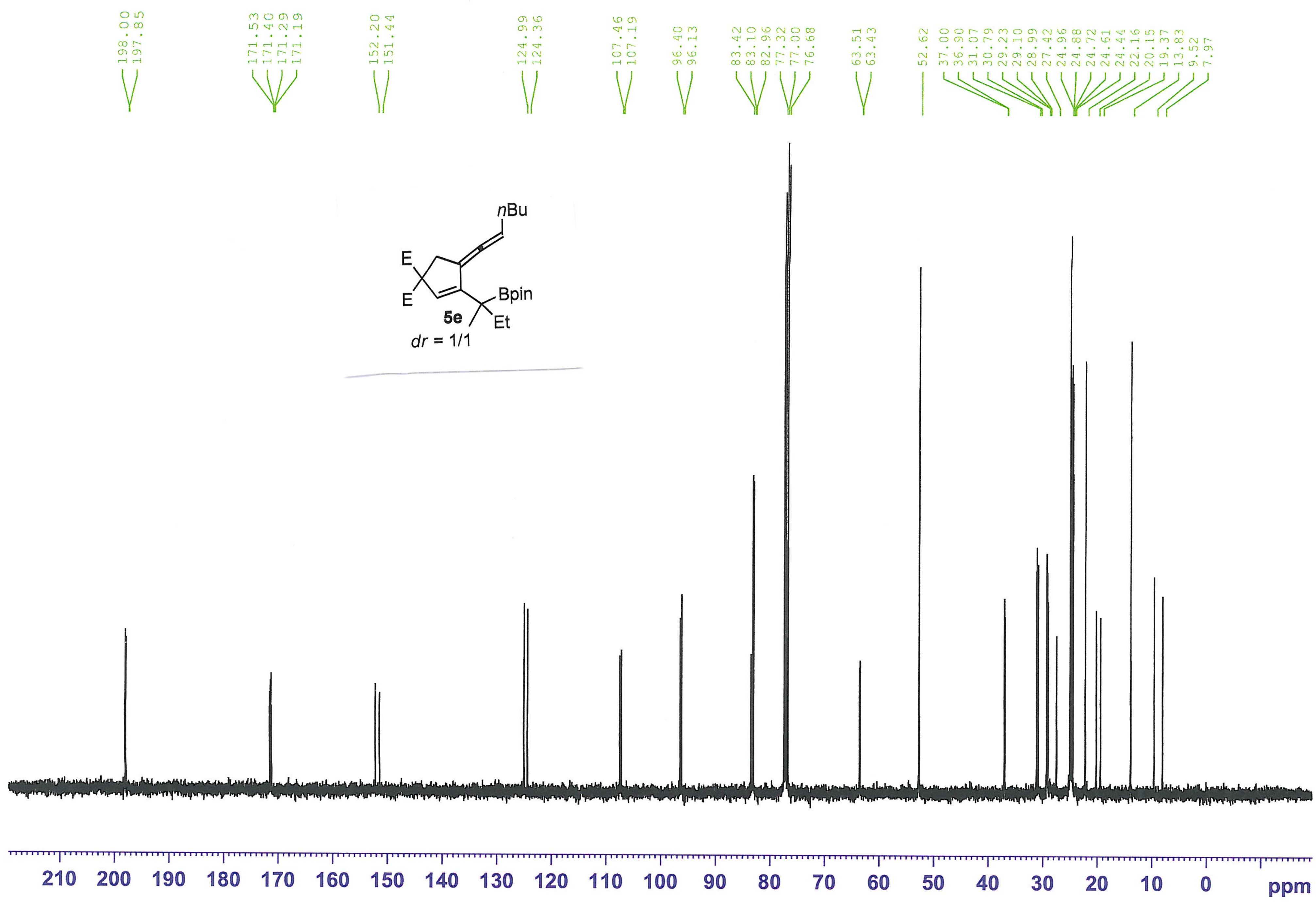

198.00
197.85
171.53
171.40
171.29
171.19
152.20
151.44
124.99
124.36
107.46
107.19
96.40
96.13
83.42
83.10
82.96
77.32
77.00
76.68
63.51
63.43
52.62
37.00
36.90
31.07
30.79
29.23
29.10
28.99
27.42
24.96
24.88
24.72
24.61
24.44
22.16
20.15
19.37
13.83
9.52
7.97
nBu
E
E
Bpin
Et
5e
dr = 1/1
210 200 190 180 170 160 150 140 130 120 110 100 90 80 70 60 50 40 30 20 10 0 ppm

Mass Spectrum SmartFormula Report

Analysis Info

Analysis Name	H:\Data2\Youqian\dyq-4-10000002.d	Acquisition Date	2012-05-28 17:22:21
Method	tune_wide_dirk.m	Operator	pia
Sample Name	dyq-4-10	Instrument / Ser#	micrOTOF 125
Comment			

Acquisition Parameter

Source Type	ESI	Ion Polarity	Positive	Set Nebulizer	0.4 Bar
Focus	Not active			Set Dry Heater	180 °C
Scan Begin	50 m/z	Set Capillary	4500 V	Set Dry Gas	4.0 l/min
Scan End	3000 m/z	Set End Plate Offset	-500 V	Set Divert Valve	Source

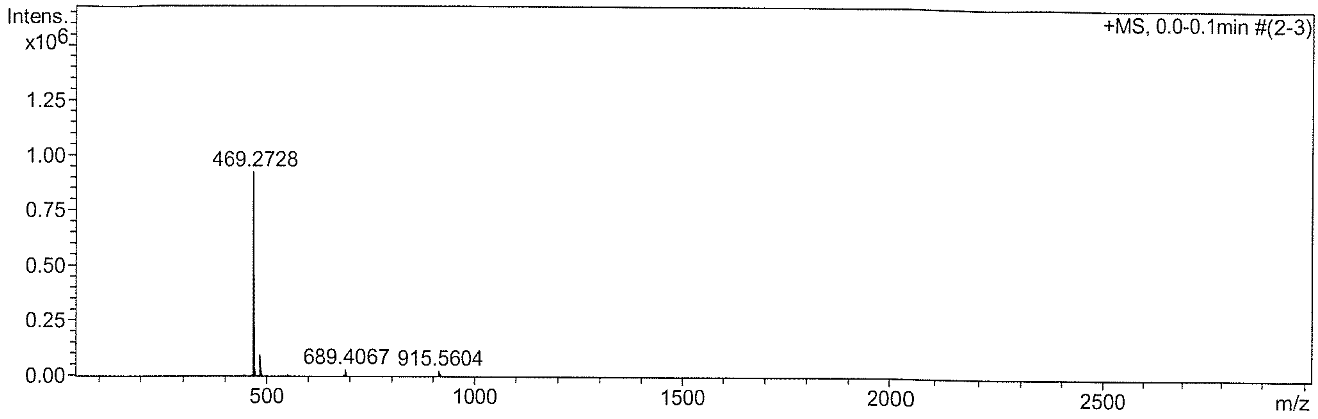

Formula	Meas. m/z	m/z	err [ppm]	Mean err [ppm]
	469.2728			
C 25 H 39 B Na O 6		469.2732	0.9	1.7

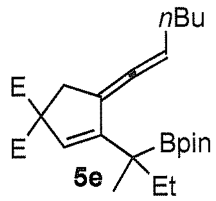

5e

dr = 1/1

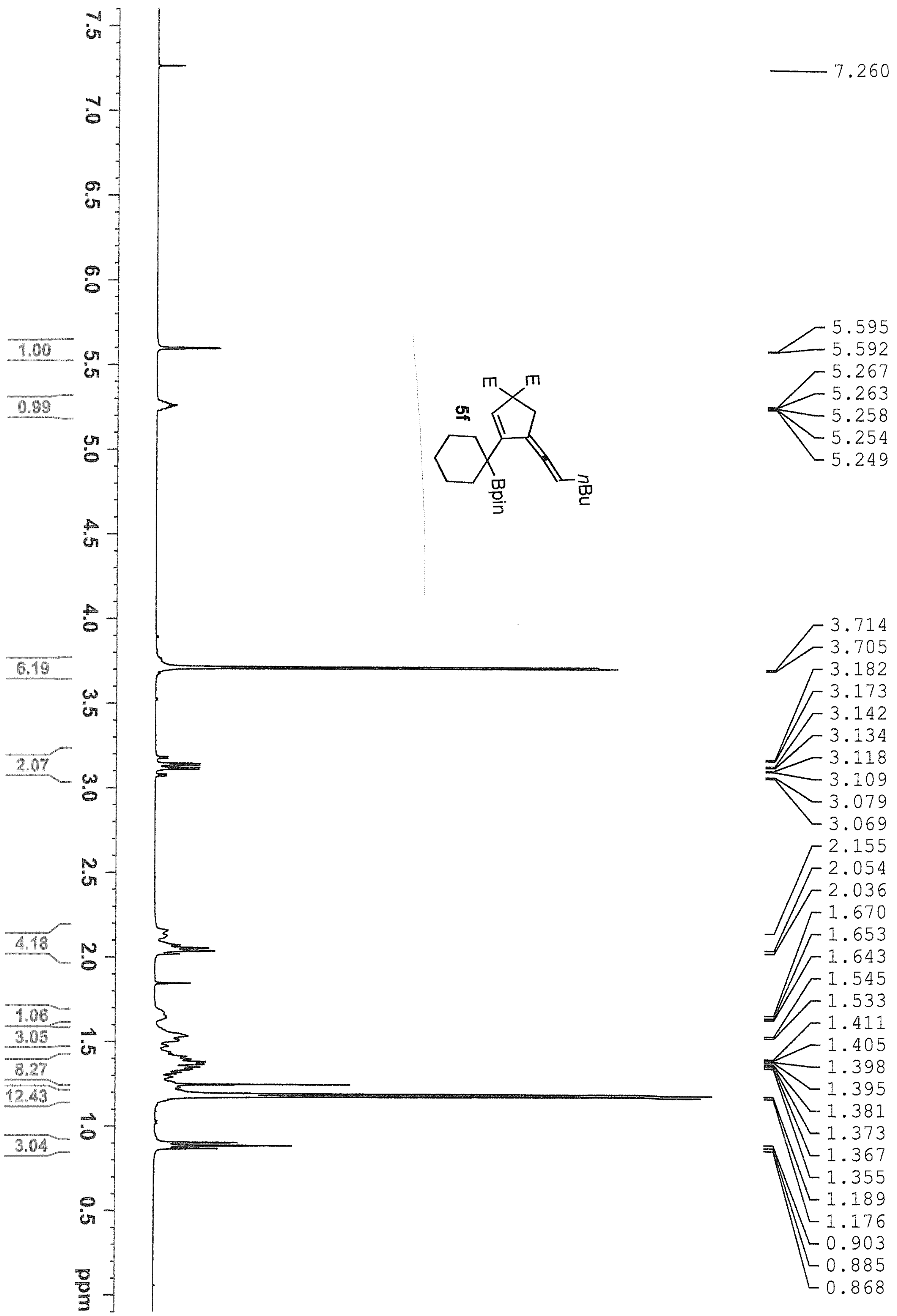

7.260
5.595
5.592
5.267
5.263
5.258
5.254
5.249
3.714
3.705
3.182
3.173
3.142
3.134
3.118
3.109
3.079
3.069
2.155
2.054
2.036
1.670
1.653
1.643
1.545
1.533
1.411
1.405
1.398
1.395
1.381
1.373
1.367
1.355
1.189
1.176
0.903
0.885
0.868
1.00
0.99
6.19
2.07
4.18
1.06
3.05
8.27
12.43
3.04
7.5
7.0
6.5
6.0
5.5
5.0
4.5
4.0
3.5
3.0
2.5
2.0
1.5
1.0
0.5
ppm
5f
E
E
nBu
Bpin

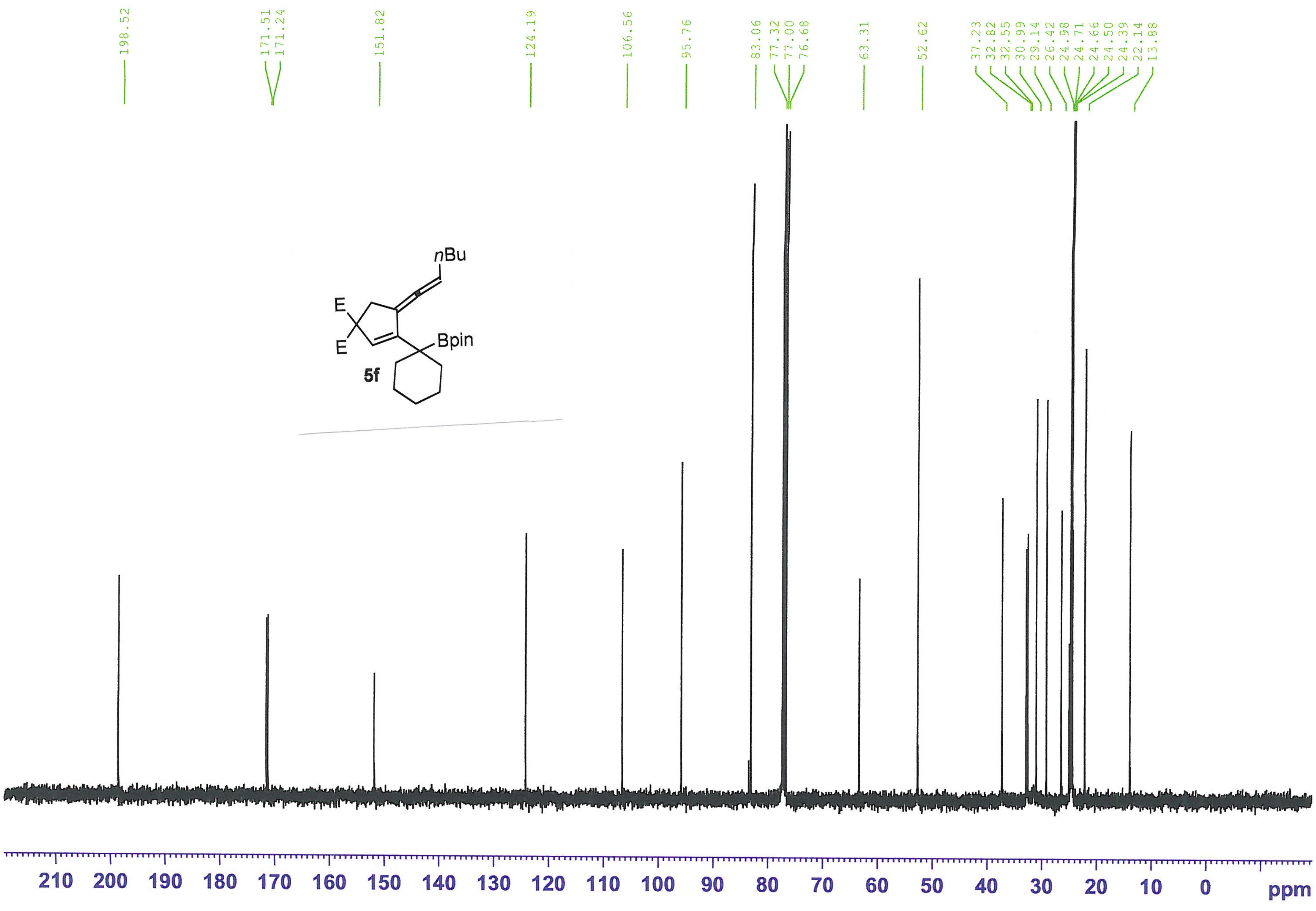
198.52
171.51
171.24
151.82
124.19
106.56
95.76
83.06
77.32
77.00
76.68
63.31
52.62
37.23
32.82
32.55
30.99
29.14
26.42
24.98
24.71
24.66
24.50
24.39
22.14
13.88
nBu
E
E
Bpin
5f
210 200 190 180 170 160 150 140 130 120 110 100 90 80 70 60 50 40 30 20 10 0 ppm

Mass Spectrum SmartFormula Report

Analysis Info

Analysis Name	H:\Data2\Youqian\dyq-3-171000001.d	Acquisition Date	2012-04-27 18:34:57
Method	tune_wide_dirk.m	Operator	pia
Sample Name	dyq-3-171	Instrument / Ser#	micrOTOF 125
Comment			

Acquisition Parameter

Source Type	ESI	Ion Polarity	Positive	Set Nebulizer	0.4 Bar
Focus	Not active			Set Dry Heater	180 °C
Scan Begin	50 m/z	Set Capillary	4500 V	Set Dry Gas	4.0 l/min
Scan End	3000 m/z	Set End Plate Offset	-500 V	Set Divert Valve	Source

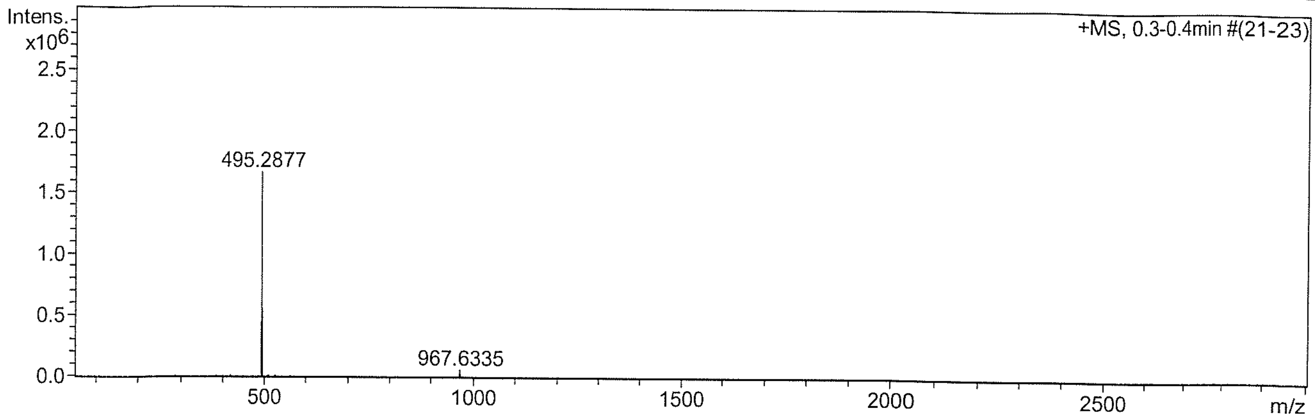

Formula	Meas. m/z	m/z	err [ppm]	Mean err [ppm]
	495.2877			
C 27 H 41 B Na O 6		495.2888	2.2	4.7

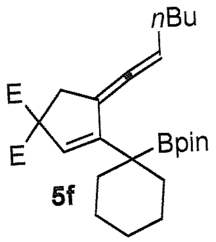

5f

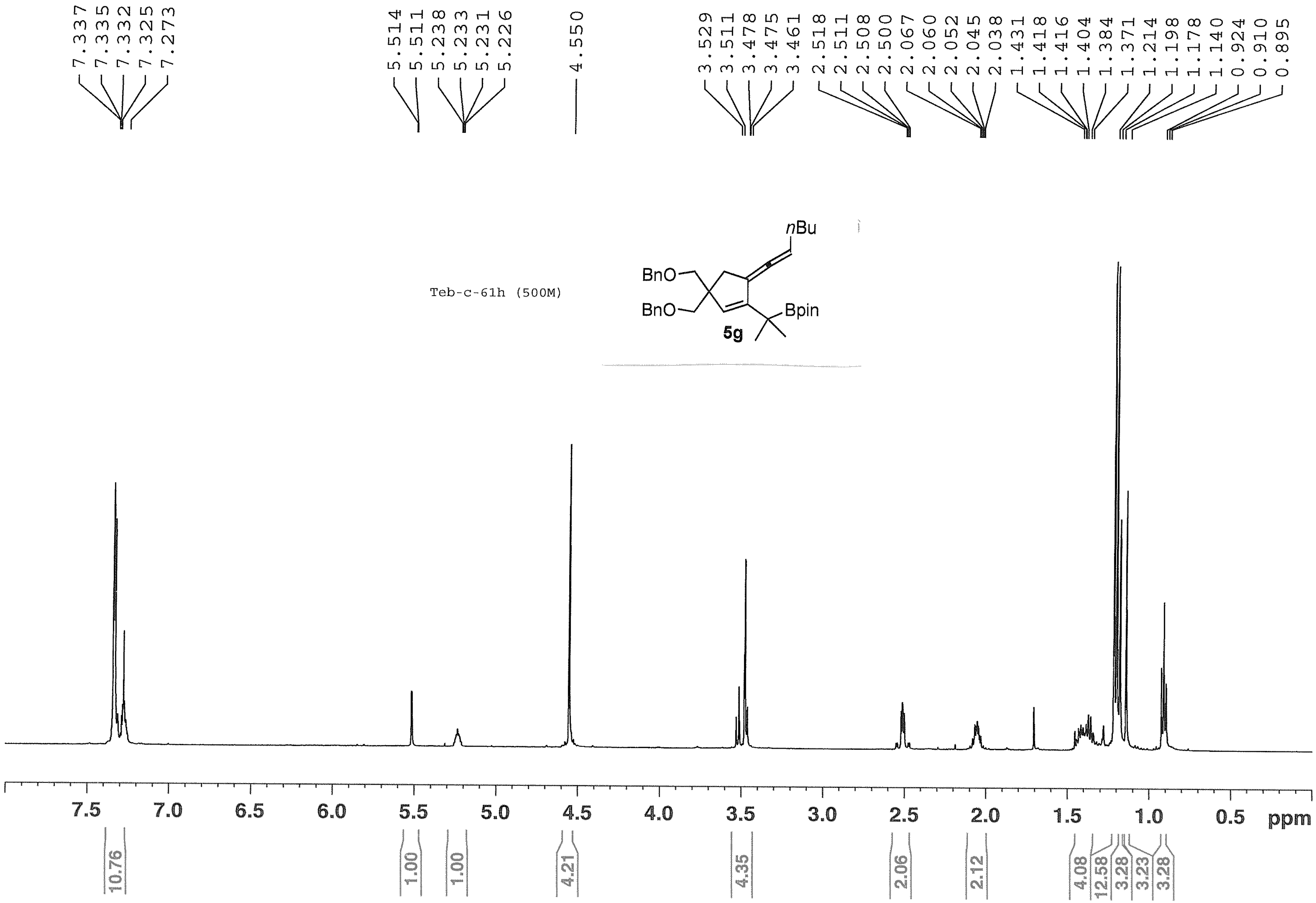
7.337
7.335
7.332
7.325
7.273
5.514
5.511
5.238
5.233
5.231
5.226
4.550
3.529
3.511
3.478
3.475
3.461
2.518
2.511
2.508
2.500
2.067
2.060
2.052
2.045
2.038
1.431
1.418
1.416
1.404
1.384
1.371
1.214
1.198
1.178
1.140
0.924
0.910
0.895
Teb-c-61h (500M)
nBu
BnO
BnO
Bpin
5g
7.5
7.0
6.5
6.0
5.5
5.0
4.5
4.0
3.5
3.0
2.5
2.0
1.5
1.0
0.5
ppm
10.76
1.00
1.00
4.21
4.35
2.06
2.12
4.08
12.58
3.28
3.23
3.28

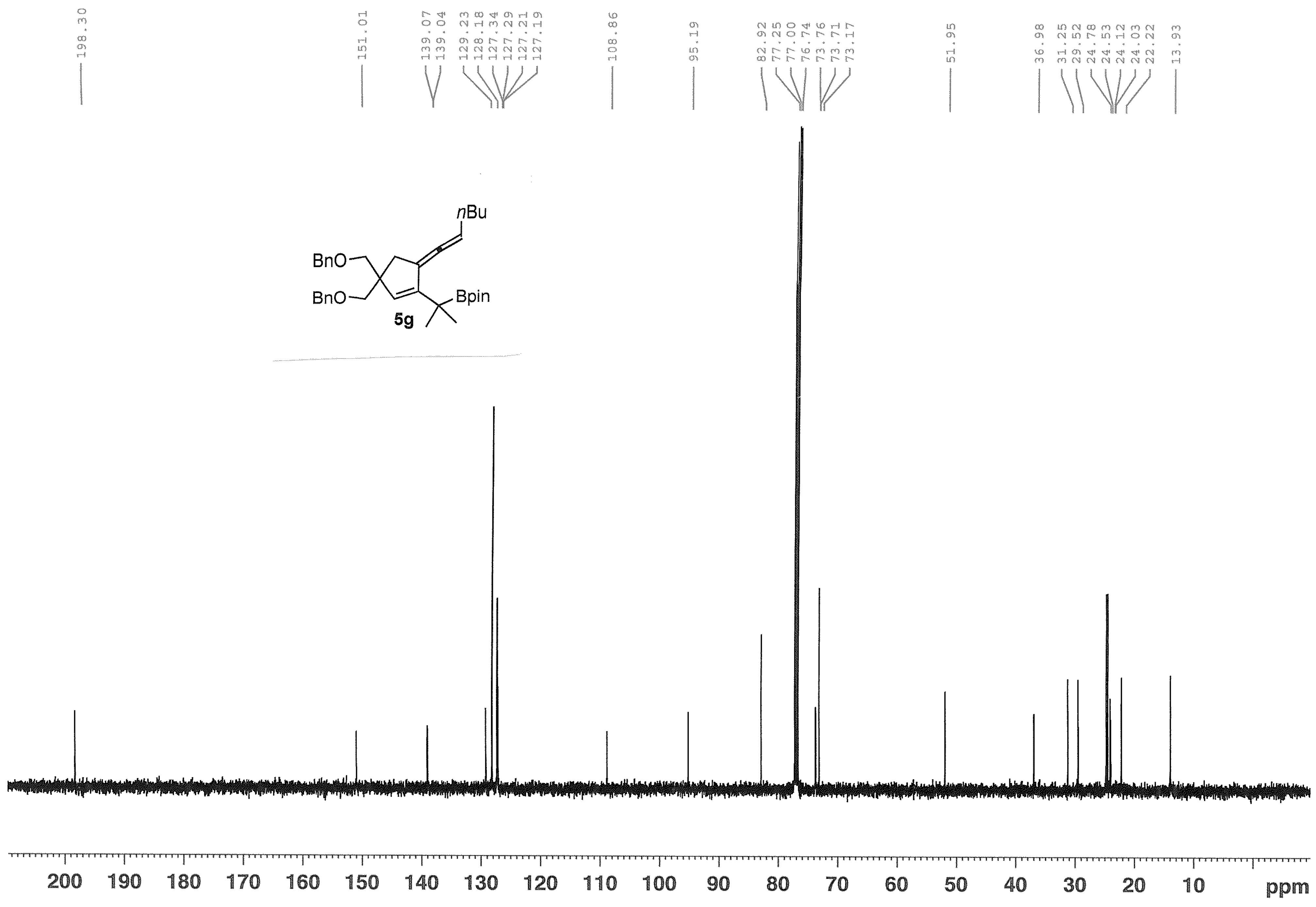
198.30
151.01
139.07
139.04
129.23
128.18
127.34
127.29
127.21
127.19
108.86
95.19
82.92
77.25
77.00
76.74
73.76
73.71
73.17
51.95
36.98
31.25
29.52
24.78
24.53
24.12
24.03
22.22
13.93
nBu
BnO
BnO
Bpin
5g
200
190
180
170
160
150
140
130
120
110
100
90
80
70
60
50
40
30
20
10
ppm

Mass Spectrum SmartFormula Report

Analysis Info

Analysis Name	E:\Data2\Youqian\Teb-c-61000001.d	Acquisition Date	2012-12-13 15:47:51
Method	Tune_low_pos.m	Operator	Carin Larsson
Sample Name	Teb-c-61	Instrument / Ser#	micrOTOF 125
Comment			

Acquisition Parameter

Source Type	ESI	Ion Polarity	Positive	Set Nebulizer	0.4 Bar
Focus	Not active			Set Dry Heater	180 °C
Scan Begin	50 m/z	Set Capillary	4000 V	Set Dry Gas	4.0 l/min
Scan End	1000 m/z	Set End Plate Offset	-500 V	Set Divert Valve	Source

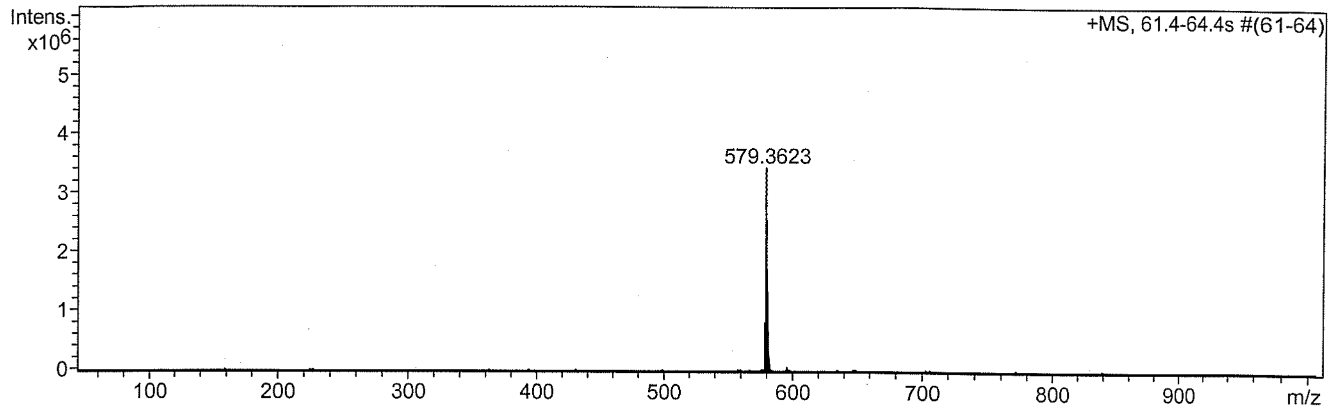

Formula	Meas. m/z	m/z	err [ppm]	Mean err [ppm]
C 36 H 49 B Na O 4	579.3623	579.3622	-0.1	0.3

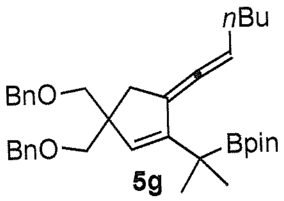

5g

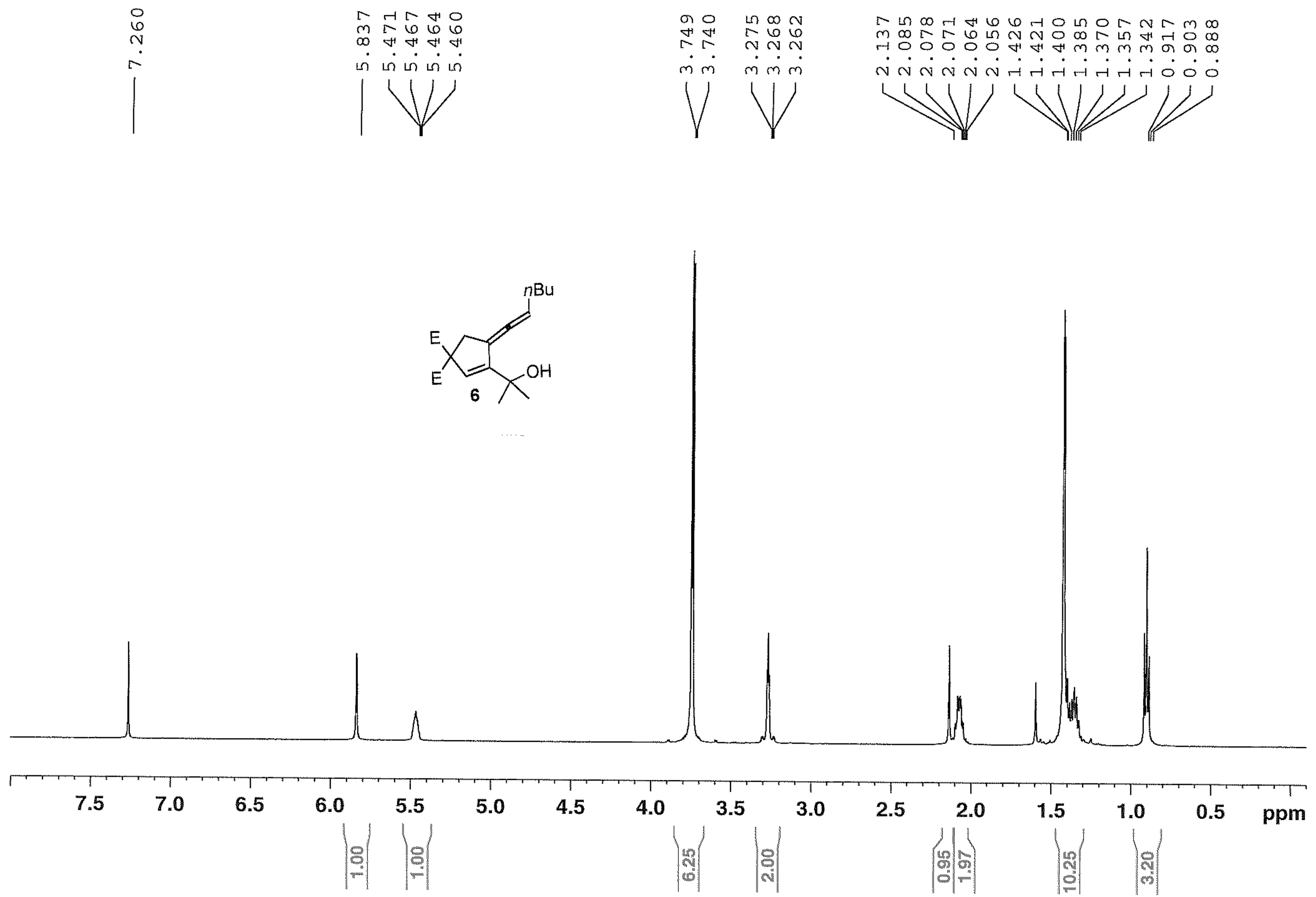

7.260
5.837
5.471
5.467
5.464
5.460
3.749
3.740
3.275
3.268
3.262
2.137
2.085
2.078
2.071
2.064
2.056
1.426
1.421
1.400
1.385
1.370
1.357
1.342
0.917
0.903
0.888
nBu
E
E
OH
6
7.5
7.0
6.5
6.0
5.5
5.0
4.5
4.0
3.5
3.0
2.5
2.0
1.5
1.0
0.5
ppm
1.00
1.00
6.25
2.00
0.95
1.97
10.25
3.20
